# Synthesis of Chalcone‐Sulfonamide Hybrids as Antibacterial, Antioxidant, and Anti‐Inflammatory Agents With Comprehensive In Silico and ADMET Profiling

**DOI:** 10.1002/open.70292

**Published:** 2026-08-27

**Authors:** Mlis Belete, Endale Mulugeta, Daniel Rentsch, Muhdin Aliye, Kebede Shenkutie, Yadessa Melaku

**Affiliations:** ^1^ Department of Applied Chemistry College of Applied Natural Science Adama Science and Technology University Adama Ethiopia; ^2^ Department of Chemistry College of Natural Sciences Kyungpook National University Taegu South Korea; ^3^ Laboratory for Functional Polymers Empa, Swiss Federal Laboratories for Materials Science and Technology Dübendorf Switzerland

**Keywords:** antibacterial activity, anti‐inflammatory activity, antioxidant activity, chalcone‐sulfonamide hybrids, Claisen–Schmidt condensation, molecular docking

## Abstract

Chalcone‐sulfonamide hybrids (**12a–h**) were synthesized via Claisen–Schmidt condensation in good yields (64%–83%) and confirmed by NMR spectroscopy. The compounds exhibited moderate to good antibacterial activity against Gram‐negative (*Escherichia coli*, *Pseudomonas aeruginosa*) and Gram‐positive (*Staphylococcus aureus*, *Streptococcus pyogenes*) bacteria, with inhibition zones of up to 14.7 mm at 10 mg/mL, lower than that of sulfamethoxazole but comparable to that of related hybrids. DPPH antioxidant evaluation revealed IC_50_ values of 5.2–11.8 μg/mL, with compounds **12e** and **12c** exhibiting the highest activity. Additionally, compound **12e** had the strongest protein denaturation inhibition (IC_50_ = 29.89  µg/mL), while **12g** and **12h** showed superior anti‐proteinase activity (IC_50_ = 115.6 and 112.2 µg/mL), outperforming diclofenac sodium. Molecular docking against DHPS (1AJ0, 1AD4), myeloperoxidase (1DNU), COX‐2 (5IKR), and topoisomerase *IIα* (4FM9) showed strong binding affinities (−8.3 to −10.3 kcal/mol), supported by key hydrogen–bonding and hydrophobic interactions. SwissADME and ProTox *II* analyses indicate favorable drug‐like properties, including Lipinski compliance, high GI absorption, no BBB penetration, and low predicted toxicity (LD_50_ > 6000 mg/kg). The hybrids presented here represent promising multifunctional leads with antibacterial, antioxidant, anti‐inflammatory, and drug‐like properties.

## Graphical abstract

Eight chalcone‐sulfonamide hybrids (**12a–h**) are synthesized via Claisen–Schmidt condensation and evaluated for antibacterial, antioxidant, and anti‐inflammatory activity. The compounds show potent radical scavenging, strong protein‐denaturation inhibition surpassing diclofenac, and favorable docking against DHPS, myeloperoxidase, COX‐2, and topoisomerase IIα. Computational ADMET profiling reveals drug‐like, low‐toxicity properties, positioning these hybrids as promising multifunctional leads against drug‐resistant infections.

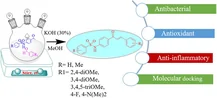

## 1 Introduction

**FIGURE 1:**
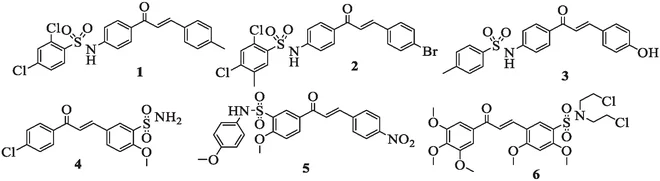
Chemical structure of reported bioactive chalcone‐sulfonamide hybrids, **1–6.**

Chalcone‐sulfonamide hybrids (Figure 1) have emerged as promising multifunctional agents against antimicrobial resistance (AMR), which claims ~1.27 million lives annually and could reach 10 million by 2050 [1]. The rapid spread of multidrug‐resistant pathogens, combined with the slow pace of new antibiotic development, has created an urgent need for novel therapeutic strategies. Unlike conventional single‐target drugs, these hybrids integrate two pharmacophores, sulfonamides and chalcones (**7**), within a single molecular framework, enhancing antimicrobial potency [2, 3] and corresponding biological activities such as antioxidant [4], Antimalarial [5], Anticancer [4, 6, 7], antileishmanial [8], and anti‐tubulin [9] activities.

Sulfonamides, defined by the sulfonyl amide functional group (─SO_2_NH─), were among the earliest synthetic antibacterial agents familiarized into clinical practice [10]. Although resistance has limited their traditional use, structural modifications have returned and expanded their antimicrobial potential [11]. Hybrid sulfonamide derivatives demonstrate efficacy against Gram‐positive and Gram‐negative bacteria, including resistant strains, through improved target binding and alternative mechanisms of action [12]. Beyond antibacterial activity, sulfonamides also exhibit antioxidant and anti‐inflammatory properties, including activation of Nrf2‐mediated antioxidant responses [13] and selective inhibition of cyclooxygenase‐2 (COX‐2) [10, 14].

**FIGURE 2:**
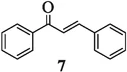
Chemical structure of chalcone (**7**).

Figure 2, Chalcones (**7**), a class of *α*,*β*‐unsaturated ketones composed of two aromatic rings linked by a conjugated enone system, are likewise recognized for their diverse biological activities [15]. Numerous chalcone derivatives have shown potent antimicrobial activity against resistant strains, sometimes better than conventional antibiotics [16]. In addition, chalcones display strong antioxidant and anti‐inflammatory effects, mediated through free radical scavenging [17], cyclooxygenase (COX) inhibition [18], and suppression of NF‐κB signaling [19]. Their structural flexibility allows for optimization of potency, safety, and multifunctionality, making them attractive scaffolds for drug development.

The hybridization of sulfonamide and chalcone pharmacophores represents a rational strategy to address the multifaceted nature of drug‐resistant infections. Excessive reactive oxygen species (ROS) and chronic inflammation drive infection persistence by causing tissue damage and enabling bacterial survival strategies like biofilm formation [20]. By combining antibacterial potency with antioxidant and anti‐inflammatory effects, chalcone‐sulfonamide hybrids may simultaneously suppress microbial growth, mitigate oxidative stress, and modulate inflammatory responses.

Studies confirm that these hybrids display improved biological activities, supporting their potential as next‐generation therapeutic candidates [6, 21, 22]. Their multifunctional design may reduce reliance on combination therapies, minimize drug–drug interactions, and provide broader clinical utility. In light of the urgent global need for agents capable of targeting resistant pathogens while protecting host tissues, the systematic design and evaluation of chalcone‐sulfonamide derivatives represent a convincing and innovative approach to drug development. In this study, we synthesized eight chalcone‐sulfonamide hybrids via Claisen–Schmidt condensation and comprehensively evaluated their antibacterial potency against Gram‐positive/negative strains, antioxidant capacity through DPPH assays, anti‐inflammatory potential, molecular docking interactions with selected protein targets, and Absorption, Distribution, Metabolism, Excretion, and Toxicity (ADMET) profiles.

## 2 Results and Discussion

### 2.1 Chemistry

**SCHEME 1:**
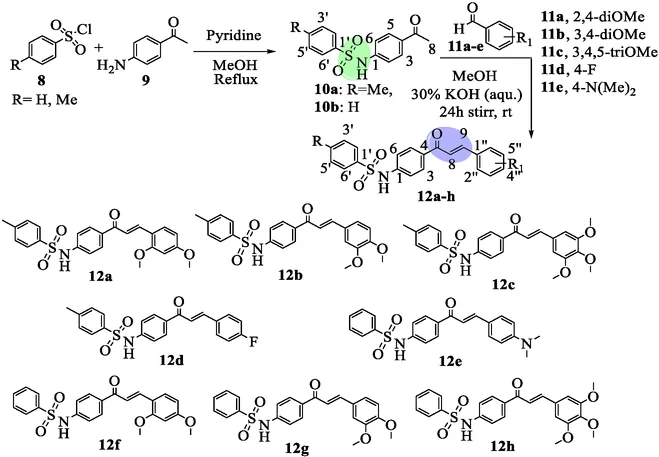
Synthesis of chalcone‐sulfonamide hybrids **12a–h.**

The chalcone‐sulfonamide hybrids (**12a–h**) were synthesized via the reaction sequences outlined in Scheme 1. These target compounds were prepared through a straightforward two‐step route involving sulfonylation followed by Claisen–Schmidt condensation [23, 24]. Initially, p‐aminoacetophenone (**9**) underwent sulfonylation with 4‐methylbenzenesulfonyl chloride/benzenesulfonyl (**8**) chloride in methanol in the presence of pyridine, affording *N*‐(4‐acetylphenyl)‐4‐methylbenzenesulfonamide (**10a**)/*N*‐(4‐acetylphenyl)benzenesulfonamide (**10b**). The observed melting point closely matched reported values in the literature [3]. The ^1^H NMR spectrum of compound **10a** displayed a distinct sulfonamide NH singlet at *δ* 10.79 ppm, aromatic proton signals between *δ* 7.20 and 7.82 ppm, and two methyl singlets at *δ* 2.44 ppm (acetyl CH_3_) and *δ* 2.30 ppm (p‐tolyl CH_3_). In the ^13^C NMR spectrum, the ketonic carbonyl carbon resonated at *δ* 196.4 ppm, while aromatic and methyl carbons appeared at their expected chemical shifts. DEPT experiments further supported the assigned structure.

In a second step, compounds **10a–b** were subjected to Claisen–Schmidt condensation with benzaldehyde derivatives (**11a–e**) in methanol using 30% KOH, yielding the desired sulfonamide‐chalcone derivatives **12a–h** in 73%–83% yield.

Successful synthesis of the sulfonamides and sulfonamide chalcones was confirmed using 1D and 2D ^1^H and ^13^C NMR spectroscopy. In most compounds, a slightly broadened ^1^H NMR signal for the sulfonamide NH proton (green area, Scheme 1) was observed at 10.8–10.9 ppm [25]. The resonances corresponding to the 4‐aminoacetophenone fragment (positions 1–6, Scheme 1) appeared at nearly identical ^1^H and ^13^C NMR chemical shifts across all derivatives, indicating that this part of the structure remained unaffected by the subsequent functionalization steps. Similarly, the signals of the benzene and of the 4‐methylbenzene subunits (positions 1′‐6′) were readily identified in all spectra based on their consistent chemical shift patterns. In compounds **10a** and **10b**, additional resonances associated with the acetyl substituent at position 4 were unambiguously assigned.

For compounds **12a–12h**, the connectivity between the acryloyl moiety (blue area, Scheme 1) at position 4 and position 1″ of the functionalized aldehydes was clearly established through long‐range ^1^H‐^13^C HMBC correlations. The 1H–1H coupling constants of 15.4–15.7 Hz measured for H 8 and H 9 are characteristic of an *E*‐configured *α*,*β*‐unsaturated system. The chemical shift ranges observed for C 8 (116–122 ppm), C 9 (138–145 ppm), and the carbonyl carbon (187–188 ppm) are in full agreement with reported values for sulfonamide chalcones [26]. Finally, the aromatic resonances of the various benzaldehyde substituents (positions 1″–6″) and of the attached functional groups were assigned using 2D 1H–1H DQF COSY, 1H–13C HSQC, and HMBC experiments. Taken together, the high isolated yields and spectroscopic assignments unequivocally confirm both the successful synthesis and the structural integrity of all sulfonamide‐chalcone hybrids. The complete set of 1D ^1^H, ^13^C, and DEPT NMR spectra is provided in Figures S1–S30.

### 2.2 Antibacterial Activity

The antibacterial activity of the synthesized chalcone‐sulfonamide derivatives (**12a–h**) was evaluated against two Gram‐negative strains, *Escherichia coli* and *Pseudomonas aeruginosa*, and two Gram‐positive strains, *Staphylococcus aureus* and *Streptococcus pyogenes*. The results were compared with the standard drug Sulfamethoxazole (**SMX**). Table 1 presents the results of the antibacterial activity evaluation of compounds **12a–h** and sulfamethoxazole as a positive control. The synthesized compounds showed clear concentration‐dependent antibacterial effects. At the highest tested concentration (10 mg/mL), zones of inhibition (ZOI) ranged from 6.3 to 14.7 mm. As the concentration decreased, activity progressively declined, and in some cases, no measurable inhibition was observed. Among the series, compound **12a** exhibited the strongest activity against *E. coli*, producing a ZOI of 14.7 ± 0.57 mm at 10 mg/mL. Compound **12b** demonstrated the best activity against *P. aeruginosa* (12 ± 1 mm). In the case of Gram‐positive bacteria, compounds **12e** and **12c** showed the highest inhibition against *S. aureus*, with ZOI values of 14.7 ± 0.57 and 14.3 ± 0.57 mm, respectively, at 10 mg/mL, suggesting that specific substituents significantly influence antibacterial potency.

**TABLE 1:** Antibacterial zone of inhibition for chalcone‐sulfonamide hybrids, **12a–h.**

Sample	Concentration, mg/mL	Zone of inhibition for synthesized compounds, mm			
		*E. coli*	*P. aeruginosa*	*S. aureus*	*S. pyogenes*
12a	10	14.7 ± 0.57	8.7 ± 0.26	12.7 ± 1.15	8 ± 0
	5	10.3 ± 0.57	6.8 ± 0.28	10 ± 1	6.7 ± 0.28
	2.5	7 ± 0	—	9.7 ± 0.57	—
	1.25	—	—	7.7 ± 0.57	—
12b	10	14 ± 1	12 ± 1	9 ± 0	12.3 ± 0.57
	5	11.7 ± 0.57	10 ± 1	7.1 ± 0.28	9.3 ± 0.57
	2.5	9.7 ± 0.57	9.3 ± 0.57	—	8 ± 1
	1.25	8.7 ± 0.57	8 ± 0	—	7 ± 1
12c	10	10.7 ± 0.25	9.7 ± 0.57	14.3 ± 0.57	12.1 ± 0.11
	5	10.3 ± 0.57	7.7 ± 0.57	14 ± 0	12 ± 0.5
	2.5	8 ± 0.6	7.3 ± 0.57	12.8 ± 0.28	9.3 ± 0.28
	1.25	—	6.3 ± 0.57	7.8 ± 0.28	6.3 ± 0.57
12d	10	11 ± 1	11 ± 1	9.1 ± 0.28	11.3 ± 0.57
	5	9 ± 0	9.3 ± 0.57	7 ± 0	9.7 ± 0.57
	2.5	7.7 ± 0.57	8.3 ± 0.57	—	8.7 ± 0.57
	1.25	—	—	—	8 ± 0
12e	10	10.1 ± 0.30	9.3 ± 0.57	14.7 ± 0.57	12.3 ± 0.57
	5	9.7 ± 0.57	8.3 ± 0.57	13.7 ± 0.57	11.7 ± 0.57
	2.5	8.3 ± 0.57	8.3 ± 0.57	12.3 ± 0.57	10 ± 0
	1.25	6.7 ± 0.57	6.7 ± 0.57	9.7 ± 0.28	8.3 ± 0.28
12f	10	13.3 ± 0.57	9.7 ± 0.57	12 ± 0.2	13.7 ± 0.57
	5	11.3 ± 0.57	8.3 ± 0.57	10.7 ± 0.57	12.1 ± 0.76
	2.5	8.3 ± 0.57	6.7 ± 0.57	10.3 ± 0.28	11.3 ± 0.57
	1.25	6.7 ± 0.57	6.3 ± 0.57	8.3 ± 0.57	7 ± 0
12g	10	13.7 ± 0.57	11.7 ± 0.57	8.7 ± 0.57	11 ± 1
	5	11.3 ± 0.57	9.7 ± 0.57	6.5 ± 0.5	9 ± 1
	2.5	8.3 ± 0.57	8.7 ± 0.57	—	8 ± 1
	1.25	—	7.7 ± 0.57	—	7.3 ± 0.57
12h	10	10.7 ± 0.57	8.7 ± 0.57	14 ± 0	12.2 ± 0.46
	5	8.7 ± 0.57	8.3 ± 0.57	10.7 ± 0.57	11.7 ± 0.57
	2.5	8.7 ± 0.57	7 ± 0	9.3 ± 0.57	9.5 ± 0.5
	1.25	6.7 ± 0.57	—	6.7 ± 0.28	6.7 ± 0.28
SMX	30 µg/mL	24.7 ± 0. 85	24.5 ± 0.65	23.6 ± 0.63	24.5 ± 0.51

Abbreviations: — = no zone of inhibition, SMX = sulfamethoxazole.

Compounds bearing methoxy‐substituted chalcone rings (**12a–c** and **12f–h**) generally displayed improved activity, particularly against Gram‐negative strains. This enhancement may be attributed to increased lipophilicity, which likely facilitates better membrane penetration. For instance, the 2,4‐dimethoxyphenyl derivative **12a** retained measurable activity against *E. coli* down to 2.5 mg/mL (7 ± 0 mm), whereas the fluoro‐substituted compound **12d** lost activity more rapidly at lower concentrations. Variations in the sulfonamide moiety also influenced antibacterial response. The p‐tolyl derivatives (**12a–d**) often demonstrated superior performance compared to their phenyl counterparts (**12e–h**). This trend was evident against *E. coli*, where **12a** (14.7 mm) showed greater inhibition than **12f** (13.3 mm).

Although none of the tested compounds matched the activity of the reference drug **SMX** (23.6–24.7 mm), several derivatives, particularly **12a** and **12e**, exhibited comparable activity relative to previously reported chalcone derivatives [27]. These findings highlight their potential as promising lead compounds in the ongoing search for new antibacterial agents to combat rising antibiotic resistance.

#### 2.2.1 Minimum Inhibitory Concentration (MIC) of Compounds 12b and 12e

To further evaluate the antibacterial potency of the most active derivatives, compounds **12b** and **12e**, selected on the basis of their zone‐of‐inhibition activity against *E. coli* and *S. aureus*, respectively, were subjected to MIC determination by the broth microdilution method against all four tested bacterial strains. Compound **12b** exhibited the strongest inhibitory activity against the Gram‐negative strains, with MIC values of 0.25 mg/mL against *E. coli* and 0.5 mg/mL against *P. aeruginosa*, but showed markedly weaker activity against the Gram‐positive strains, with MIC values of 2 mg/mL against both *S. aureus* and *S. pyogenes*. In contrast, compound **12e** displayed the opposite selectivity pattern, showing pronounced activity against the Gram‐positive strains, particularly *S. aureus* (MIC = 0.125 mg/mL), followed by *S. pyogenes* (MIC = 0.5 mg/mL), while its activity against the Gram‐negative strains was comparatively modest, with an MIC of 1 mg/mL against both *E. coli* and *P. aeruginosa*. These MIC results are in good agreement with the trends observed in the zone‐of‐inhibition assay (Table 1), reinforcing **12b** as the more promising candidate against Gram‐negative pathogens and **12e** as the more promising candidate against Gram‐positive pathogens, and supporting the hypothesis that the substituent pattern on the sulfonamide/B‐ring governs strain‐selective antibacterial activity within this chalcone‐sulfonamide series.

### 2.3 Antioxidant Activity

**FIGURE 3:**
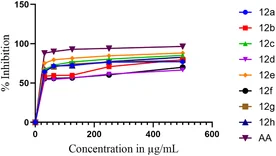
Percentage inhibition of the synthesized compounds (**12a–h**) and ascorbic acid at different concentrations.

The radical scavenging activities of synthesized compounds **12a–h** and the standard antioxidant ascorbic acid were assessed by using the DPPH method [28]. The IC_50_ values, representing the concentration required to scavenge 50% of the DPPH radicals, were determined for each compound, and the corresponding data are presented in Table 2 and Figure 3. All tested compounds demonstrated noticeable radical scavenging effects, with IC_50_ values ranging between 5.2 and 11.8 µg/mL. In comparison, the reference standard ascorbic acid (AA) exhibited stronger activity, with an IC_50_ value of 3.0 µg/mL. Furthermore, all synthesized compounds exhibited clear dose‐dependent antioxidant behavior, as the percentage of radical scavenging increased consistently with rising concentrations across the tested range (31.25–500 µg/mL).

**TABLE 2:** DPPH radical scavenging assay of synthesized chalcone‐sulfonamide hybrid **12a–h**.

Compounds	% Inhibition of synthesized compounds in µg/mL					IC_50_,
	500	250	125	62.5	31.25	µg/mL
12a	77.17 ± 0.19	76.59 ± 0.133	72.74 ± 0.22	71.41 ± 0.064	64.39 ± 0.074	6.5
12b	79.68 ± 0.037	70.84 ± 0.11	59.98 ± 0	59.64 ± 0.037	58.5 ± 0.064	11.8
12c	85.18 ± 0.037	80.39 ± 0.037	76.81 ± 0.064	72.83 ± 0.128	66.57 ± 0.037	8.9
12d	66.62 ± 0.037	61.31 ± 0.037	56.38 ± 0.064	54.63 ± 0.037	54.54 ± 0.037	6.7
12e	88.14 ± 0.037	84.64 ± 0.037	81.5 ± 0.074	79.55 ± 0	75.37 ± 0.037	5.2
12f	70 ± 0	60.47 ± 0.037	56.68 ± 0.037	56.06 ± 0	55.72 ± 0.037	6.7
12g	79.08 ± 0.098	76.81 ± 0.064	74.13 ± 0.074	70.56 ± 0.064	65.74 ± 0.037	6.7
12h	82.83 ± 0.037	77.34 ± 0.037	72.16 ± 0.037	71.84 ± 0.037	67.47 ± 0.037	6.6
AA	96.60 ± 0	94.05 ± 0.098	92.91 ± 0.074	90.06 ± 0.037	88.05 ± 0.064	3.0

Abbreviation: AA = ascorbic acid.

Among the series, compound **12e**, containing a *p*‐dimethylamino group, showed the highest percentage inhibition at 500 µg/mL (88.1%). This was closely followed by compound **12c**, which contains a 3,4,5‐trimethoxy groups 85.2% inhibition at a higher concentration. The superior performance of these derivatives can likely be attributed to the presence of strong electron‐donating groups [29]. The 3,4‐dimethoxy‐substituted derivatives (**12b** and **12g**) displayed moderate antioxidant activity. Notably, compound **12g** exhibited greater potency than **12b**, implying that subtle electronic variations arising from substitution on the sulfonamide phenyl ring can significantly impact antioxidant performance. Compounds **12a**, **12f**, **12g**, **12h**, and **12d** showed closely comparable IC_50_ values within the range of 6.5–6.7 µg/mL, better than related reports [4], suggesting similar antioxidant capacities despite structural differences. Although none of the synthesized compounds outperformed ascorbic acid, their moderate to strong radical scavenging activities underscore the potential of the chalcone‐sulfonamide hybrid framework as a valuable target for further development.

### 2.4 Anti‐Inflammatory Assay

The anti‐inflammatory activities of the synthesized compounds were evaluated using protein denaturation inhibition and anti‐proteinase assays. Protein denaturation is a well‐recognized mechanism associated with inflammatory processes, and compounds capable of preventing protein denaturation may exhibit anti‐inflammatory potential. The anti‐proteinase assay assesses the ability of compounds to inhibit proteolytic enzymes involved in tissue damage during inflammation [30]. These complementary in vitro methods provide a rapid and reliable approach to screen compounds for anti‐inflammatory activity. The results help to identify compounds capable of stabilizing proteins and inhibiting protease‐mediated inflammatory pathways.

#### 2.4.1 Anti‐Protein Denaturation Assay

**FIGURE 4:**
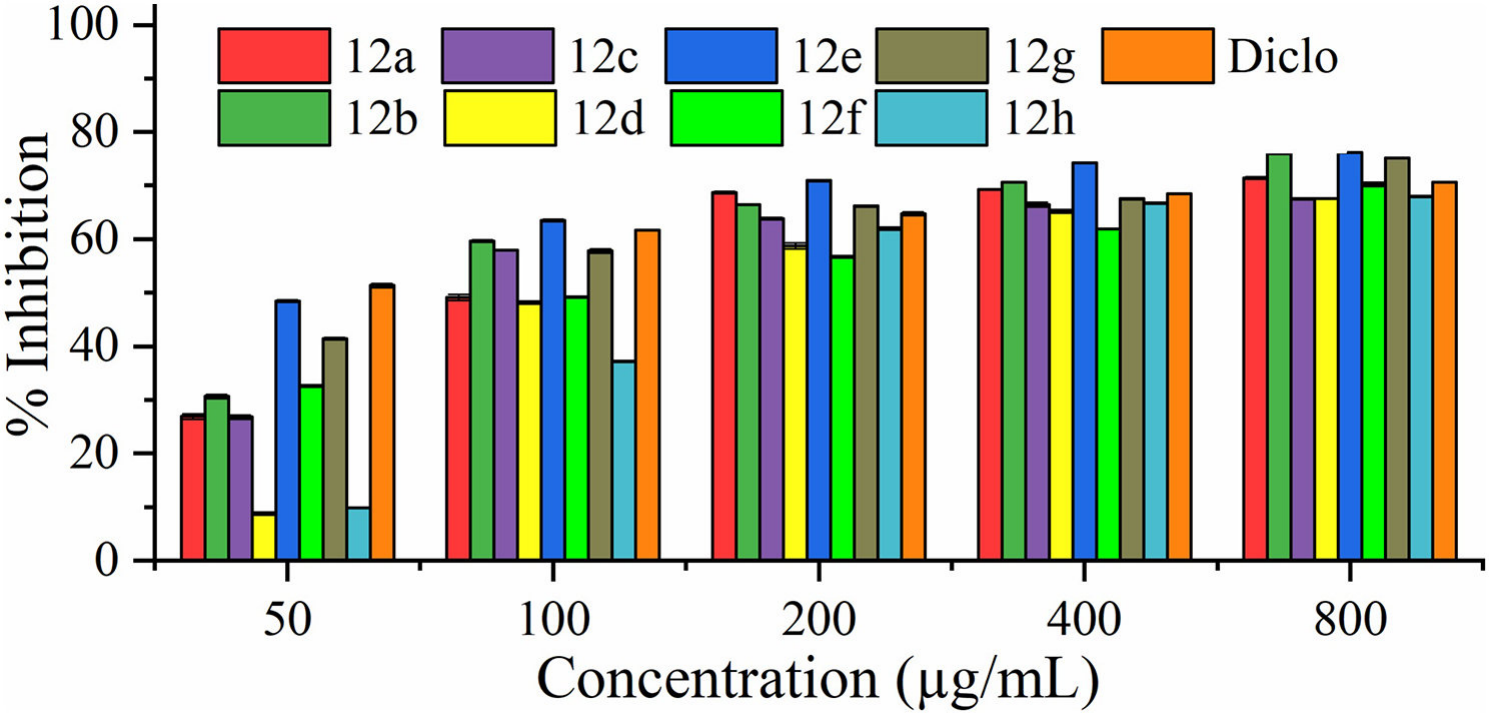
Bovine serum albumin (BSA) denaturation assay results of compounds **12a–12h**.

The synthesized chalcone‐sulfonamide derivatives (**12a–h**) were evaluated in vitro for their protein denaturation inhibitory potential, and the results in Figure 4 demonstrated a clear dose‐dependent trend across all tested concentrations. Several of the compounds demonstrated high inhibition percentages at the maximum concentration of 800 µg/mL; however, the reference drug Diclofenac sodium remained the most potent overall with the lowest IC_50_ of 19.36 µg/mL. Among the tested molecules, compound **12e** stood out as the most active member of the series, showing 76.17 ± 0.12% inhibition at 800 µg/mL and 48.44 ±  0.19% at 50 µg/mL, resulting in an IC_50_ of 29.89 µg/mL (Table 3). This strong performance is likely related to the *p*‐dimethylamino group present on the B‐ring together with the unsubstituted benzenesulfonamide fragment, where the electron‐donating nature of the dimethylamino substituent may enhance interactions with the biological target through pi–pi stacking or hydrogen bonding.

**TABLE 3:** IC_50_ values of synthesized compounds **12a–12h**.

Compounds	Bovine serum albumin (BSA) denaturation assay	Anti‐proteinase assays
12a	70.5	220.6
12b	58.0	367.0
12c	53.5	400.8
12d	103.3	257.1
12e	29.9	349.5
12f	57.8	191.2
12g	39.7	115.6
12h	124.1	112.2
Diclo	19.4	152.4

Abbreviation: Diclo = diclofenac sodium.

On the other hand, modifications to the sulfonamide ring appeared to influence activity, as seen when comparing **12e** to its counterpart **12g** (75.12 ± 0.07% at 800 µg/mL) (Figure 4), which features a *p*‐methyl group (tosyl group) and a higher IC_50_ of 39.67 µg/mL (Table 3). While the B‐ring substitution plays a significant role, the data indicate that the unsubstituted sulfonamide in **12e** provided superior potency over the methylated version in **12g**. In contrast, compounds with halogen substitution (**12d**) or the bulkier 3,4,5‐trimethoxy pattern (**12h**) displayed noticeably lower inhibition at reduced concentrations, reaching only 8.8 ± 0.26% and 9.83 ± 0.12%, respectively, at 50 µg/mL (Figure 4). These derivatives also exhibited the highest IC_50_ values in the study (103.3 and 124.1 µg/mL); Table 3, likely due to steric hindrance or less favorable electronic properties interfering with target binding. Taken together, these findings highlight compound **12e** as the most promising lead candidate, showing the strongest and most consistent inhibitory activity across the tested concentrations within the synthesized series.

#### 2.4.2 Anti‐Proteinase Assays

**FIGURE 5:**
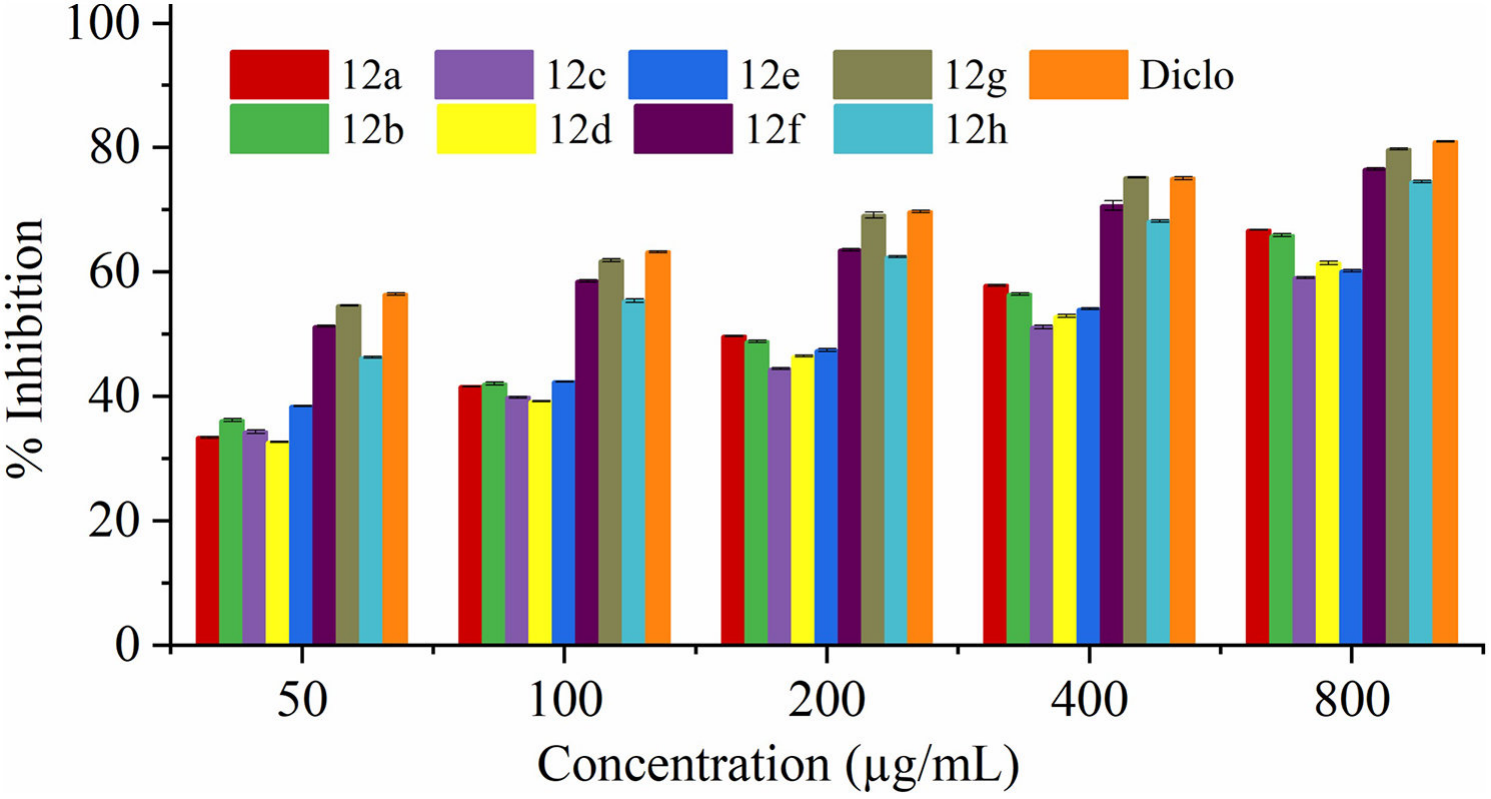
Anti‐proteinase assay results of synthesized compounds **12a–12h**.

The anti‐proteinase activity of the synthesized chalcone‐sulfonamide derivatives (**12a–h**) was evaluated using the trypsin‐casein assay. All compounds exhibited concentration‐dependent inhibition over the tested range (50–800 µg/mL), indicating their ability to suppress proteolytic enzyme activity associated with inflammatory processes (Figure 5).

Among the series, **12g** and **12h** displayed the strongest activity, with 79.75 ± 0.15% and 74.55 ± 0.20% inhibition at 800 µg/mL and the lowest IC_50_ values (115.6 and 112.2 µg/mL, respectively), surpassing the reference drug diclofenac sodium (IC_50_ = 152.4 µg/mL); Table 3. The enhanced activity of these derivatives may be attributed to the presence of electron‐donating methoxy substituents, particularly the 3,4‐dimethoxy and 3,4,5‐trimethoxy patterns, which likely facilitate strong interactions with the enzyme active site.

Compound **12f** also showed notable inhibition (76.56% at 800 µg/mL; IC_50_ = 191.2 µg/mL); Table 3 and Figure 5, whereas derivatives **12a–12e** exhibited comparatively moderate activity (59.12%–66.77% inhibition at 800 µg/mL) with higher IC_50_ values. These results suggest that methoxy substitution on the aromatic ring enhances anti‐proteinase activity, especially when combined with the benzenesulfonamide moiety, suggesting that these derivatives could be developed further to combat inflammatory diseases.

### 2.5 Molecular Docking Study

Molecular docking is a fundamental technique in structure‐based drug design that has been widely used in computational drug discovery [31]. It aims to predict the preferred binding orientation, conformation, and position of a small molecule within the active site of a macromolecular target by exploring possible ligand–receptor interaction modes. This approach enables the estimation of binding affinities and provides detailed insights into key intermolecular interactions, including hydrogen bonding, hydrophobic contacts, and electrostatic forces. Consequently, molecular docking serves as a rapid and cost‐effective tool for assessing ligand‐target complementarity and prioritizing promising compounds for further investigation.

#### 2.5.1 Against *E. coli* Dihydropteroate Synthase (DHPS, PDB ID: 1AJ0)

Dihydropteroate synthase (DHPS) is a key enzyme in the folate biosynthesis pathway of bacteria, catalyzing the condensation of para‐aminobenzoic acid (PABA) with dihydropterin pyrophosphate to form dihydropteroate, an essential precursor for nucleotide synthesis. Since mammalian cells lack this pathway, DHPS represents a selective and well‐validated target for antibacterial drug development [32]. Sulfonamide‐based drugs, such as sulfamethoxazole, act as structural analogs of PABA and competitively inhibit DHPS by occupying its active site [33]. Therefore, the incorporation of a sulfonamide pharmacophore into the designed chalcone derivatives provides a rational structural basis for targeting DHPS. In this study, the designed chalcone‐sulfonamide derivatives were evaluated for their ability to interact with the DHPS active site using molecular docking.

**FIGURE 6:**
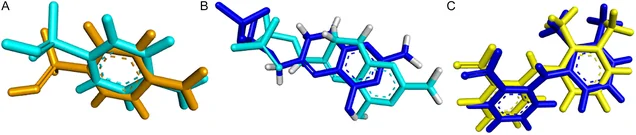
Validation of the molecular docking protocol by re‐docking co‐crystallized ligands into their respective protein structures. Superimposition of experimental (crystallographic) and re‐docked ligand poses is shown (A) for 1AJ0 (sulfanilamide, yellow; RMSD = 1.29 Å), (B) for 1AD4 (6‐hydroxymethyl‐7,8‐dihydropterin pyrophosphate, blue; RMSD = 1.82 Å), and (C) for 5KIR (mefenamic acid, blue; RMSD = 0.59 Å), demonstrating good to excellent agreement between predicted and native conformations.

In this study, the docking analysis for *E. coli* dihydropteroate synthase (DHPS, PDB ID: 1AJ0) was used to complement the in vitro antibacterial results and to provide a mechanistic explanation for the activity of the synthesized chalcone‐sulfonamide hybrids. Before evaluating the target compounds, the docking protocol was validated by re‐docking the co‐crystallized sulfanilamide ligand into the DHPS active site. The re‐docked pose (Figure 6A) showed good agreement with the crystallographic orientation, with an RMSD of 1.29 Å, which is well below the commonly accepted cutoff of 2.0 Å. This confirms that the docking settings were reliable for predicting ligand binding within the DHPS pocket [34, 35].

**FIGURE 7:**
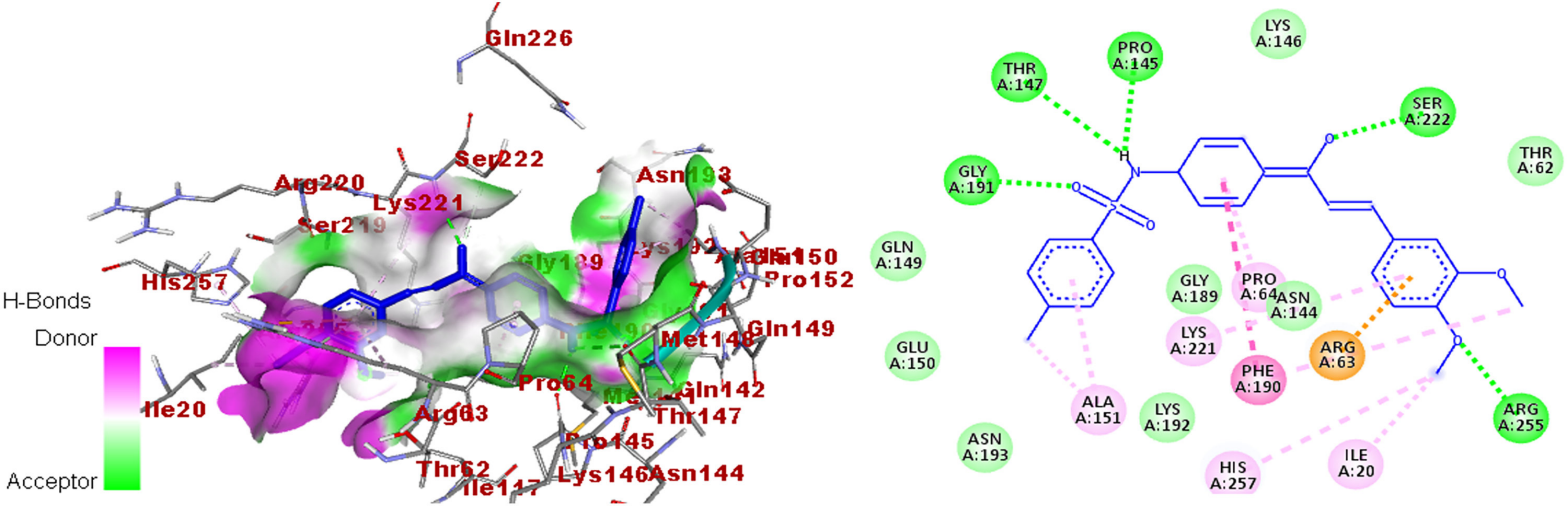
3D (left) and 2D (right) representations of the binding interactions of compound **12b** against *E. coli* DHPS (PDB ID: 1AJ0).

The binding mode analysis of sulfanilamide (Figure 7) reveals the critical role of hydrogen–bonding interactions involving Asn22, Thr62, Arg63, and Arg255 residues. In addition, hydrophobic interactions with Arg63, Arg255, His257, Lys221, Phe190, and Ile20 further stabilize the ligand‐enzyme complex. These findings are consistent with previous studies, which have emphasized the importance of these key residues in the active site of DHPS [36].

The validated protocol was then applied to the synthesized compounds **12a–h**. The docking scores against 1AJ0 ranged from −8.5 to −8.9 kcal/mol, indicating favorable binding for all derivatives and stronger affinity than the reference compound sulfamethoxazole (−7.1 kcal/mol) (Table 4). Among the series, compound **12b** exhibited the most favorable binding energy, in agreement with its moderate antibacterial performance in vitro against *E. coli* and *P. aeruginosa* at lower concentrations.

**TABLE 4:** The binding affinities (kcal/mol) for the best binding position docked to the target macromolecules (PDB ID: 1AJ0, 1AD4, 1DNU, 5IKR, and 4FM9) of compounds **12a–h**.

Targets	Binding affinities of synthesized compounds, kcal/mol								
	12a	12b	12c	12d	12e	12f	12g	12h	Standard
1AJ0	−8.8	−8.9	−8.7	−8.8	−8.5	−8.6	−8.7	−8.8	−7.1^a^
1AD4	−7.0	−6.9	−6.7	−7.5	−6.8	−7.0	−6.9	−6.3	−6.6^a^
1DNU	−9.5	−9.5	−9.3	−9.8	−9.6	−9.4	−9.6	−8.8	−6.1^b^
5IKR	−8.6	−8.6	−8.4	−8.9	−9.5	−8.5	−9.4	−9.1	−8.5^c^
4FM9	−8.9	−9.8	−9	−9.7	−9.8	−8.8	−9.3	−8.9	−9.7^d^

a Sulfamethoxazole (SMX).

b Ascorbic acid.

c Diclofenac.

d Etoposide.

All compounds exhibited hydrogen–bonding interactions with at least one key residue within the enzyme’s active site, while additional hydrophobic contacts contributed to overall complex stabilization depending on the ligand conformation (Table S1). A detailed analysis of the interaction profile of compound **12b**, identified as the most active derivative at lower concentration against *E. coli*, reveals extensive engagement with several critical residues. Specifically, hydrogen bonds were observed with Gly191, Ser222, Arg255, Pro145, Thr147, and Arg63. These interactions include the sulfonamide oxygen forming a hydrogen bond with the amine group of Gly191, the amide hydrogen interacting with Thr147 and Pro145, and the carbonyl and methoxy oxygen atoms forming hydrogen bonds with Ser222 and Arg255, respectively (Figure 7).

Beyond these polar interactions, Arg63 plays a dual stabilizing role. Its guanidinium NH_2_ group participates in a π–cation interaction with the ligand’s aromatic ring, while its side chain also forms a π‐donor hydrogen bond. This dual electrostatic and hydrogen‐bonding behavior is consistent with the established role of Arg63 in anchoring pABA‐mimetic ligands, including the co‐crystallized sulfanilamide, within the same binding pocket. Further hydrophobic stabilization is provided by Phe190, which engages in both π–π T‐shaped and π–alkyl interactions with the ligand’s aromatic system. Additional alkyl and π‐alkyl contacts from Ala151, Ile20, His257, Pro64, and Lys221 further reinforce the stability of the ligand‐enzyme complex.

**FIGURE 8:**
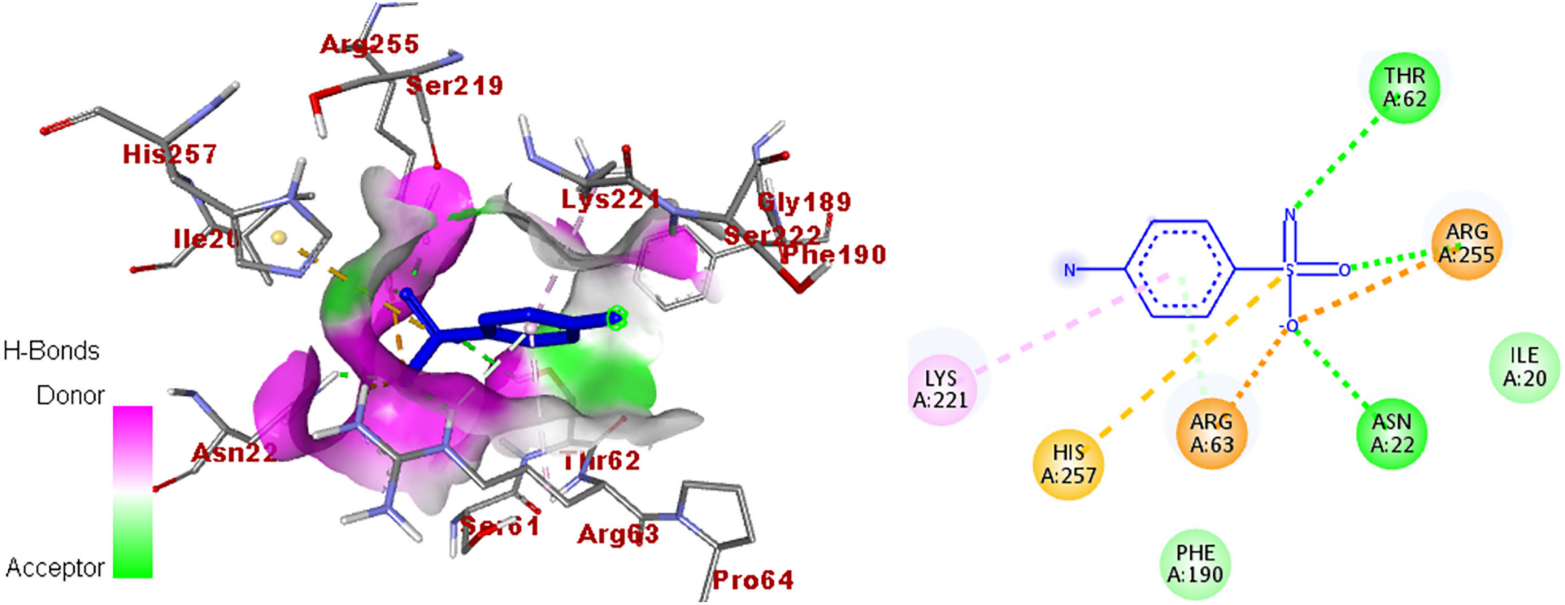
3D (left) and 2D (right) representations of the binding interactions of sulfanilamide against *E. coli* DHPS (PDB ID: 1AJ0).

**FIGURE 9:**
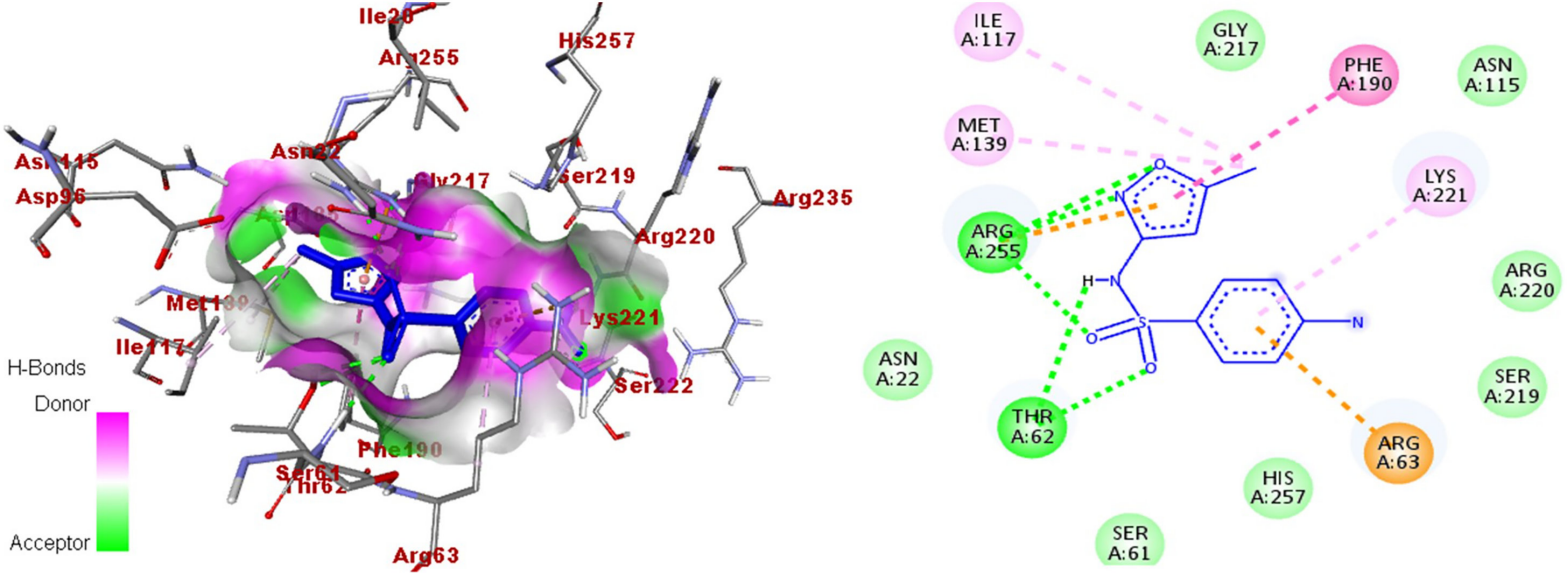
3D (left) and 2D (right) representations of the binding interactions of sulfanilamide against *E. coli* DHPS (PDB ID: 1AJ0).

Overall, the docking results suggest that the designed chalcone‐sulfonamide derivatives can effectively interact with key residues within the DHPS active site, supporting their potential as antibacterial candidates. These findings provide a rational basis for further structural optimization and biological evaluation. The binding affinities, hydrogen‐bonding patterns, and residue interactions of the synthesized compounds are summarized in Table S1. The 2D and 3D binding interactions of compound **12b,** sulfanilamide and **SMX** are illustrated in Figures 7, 8, 9, respectively, while the corresponding diagrams for the other compounds are provided in the Supporting Information. Hydrogen bonds are indicated by green dashed lines, hydrophobic interactions by pink dashed lines, and electrostatic interactions with key amino acid residues are shown as orange dashed lines (Figures S31–S39).

#### 2.5.2 Against *S. aureus* Dihydropteroate Synthase (DHPS, PDB ID: 1AD4)

Having established the structural basis for the synthesized hybrids’ activity against Gram‐negative *E. coli* DHPS, attention was next turned to the Gram‐positive target, *S. aureus* DHPS, to rationalize the differential antibacterial spectrum observed within the series, particularly the pronounced activity of compound **12e** against Gram‐positive strains. In this study, the docking analysis for *S. aureus* dihydropteroate synthase (DHPS, PDB ID: 1AD4) was used to complement the in vitro antibacterial results and to provide a mechanistic explanation for the activity of the synthesized chalcone‐sulfonamide hybrids. Before evaluating the target compounds, the docking protocol was validated by re‐docking the co‐crystallized 6‐hydroxymethyl‐7,8‐dihydropterin pyrophosphate ligand into the DHPS active site. The re‐docked pose (Figure 6B) showed good agreement with the crystallographic orientation, with an RMSD of 1.82 Å, well below the commonly accepted cutoff of 2.0 Å, confirming that the docking settings were reliable for predicting ligand binding within the DHPS pocket [34, 35].

**FIGURE 10:**
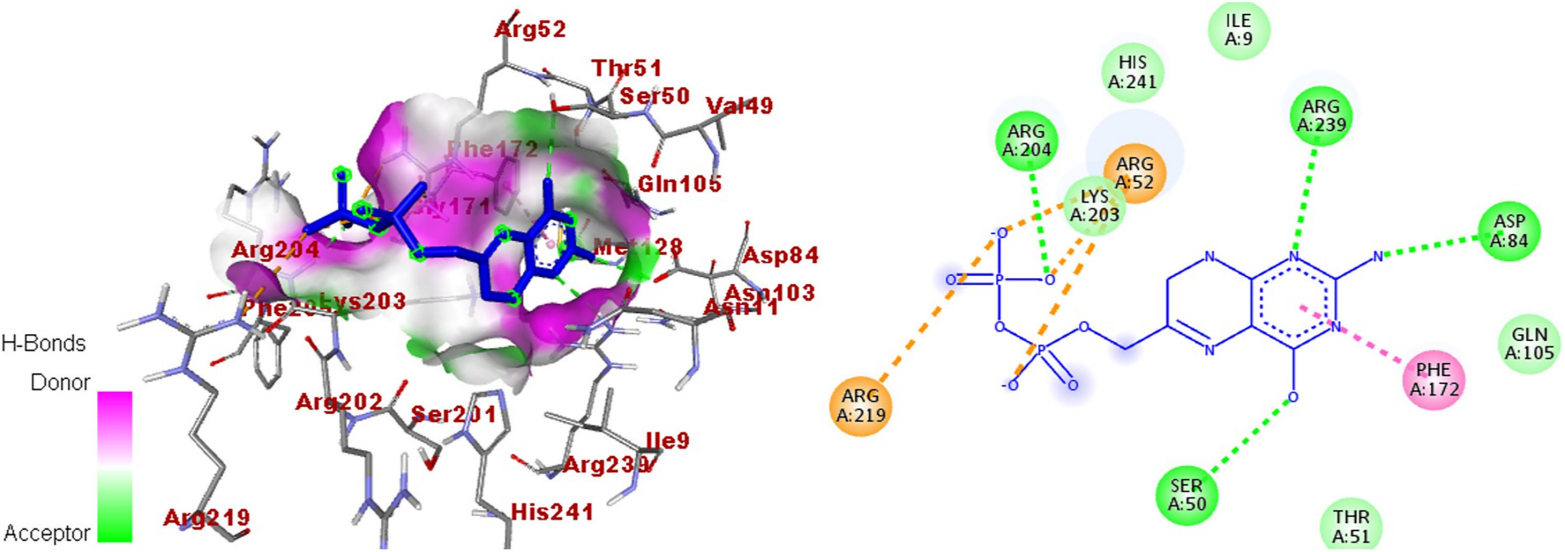
3D (left) and 2D (right) representations of the binding interactions of the co‐crystallized ligand against *S. aureus* DHPS (PDB ID: 1DA4).

The binding mode analysis of 6‐hydroxymethyl‐7,8‐dihydropterin pyrophosphate (Figure 10) reveals the critical role of hydrogen–bonding interactions involving Arg204, Arg239, Asp84, and Ser50 residues. In addition, hydrophobic interactions with Arg219, Arg52, and Phe172 further stabilize the ligand‐enzyme complex, while van der Waals contacts with His241, Ile9, Gln105, Thr51, and Lys203 line the surrounding binding pocket. These findings are consistent with previous studies, which have emphasized the importance of these key residues in the active site of DHPS [37].

Following validation, the synthesized derivatives (**12a–12h**) were docked against the *S. aureus* DHPS active site, with binding affinities ranging from −6.3 to −7.5 kcal/mol (Table 4). Notably, the majority of the synthesized compounds exhibited binding affinities equal to or more favorable than the reference drug **SMX** (−6.6 kcal/mol), and several matched or exceeded the affinity of the co‐crystallized ligand (−6.9 kcal/mol), indicating that the chalcone‐sulfonamide scaffold is generally well accommodated within this active site. All compounds exhibited hydrogen–bonding interactions with at least one key residue within the enzyme’s active site, while additional hydrophobic contacts contributed to overall complex stabilization depending on the ligand conformation (Table S2). This favorable binding profile across the series was further supported by a consistent hydrogen‐bonding pattern engaged by the majority of the synthesized derivatives as well as by both SMX and the co‐crystallized ligand, suggesting that the hybrids occupy the same pterin/pABA‐binding subsite exploited by the native substrate and the reference sulfonamide drug.

A detailed analysis of the interaction profile of compound **12e**, identified as the most active derivative at lower concentration against *S. aureus* and *S. pyogenes*, revealed a binding affinity of −6.8 kcal/mol within the DHPS active site (Table S2). The interaction shows that **12e** forms three conventional hydrogen bonds: one with Gln105 and two with Arg239, the latter engaging the ligand through both of its guanidinium hydrogens in a bidentate fashion. This dual hydrogen‐bonding contact from Arg239, together with the Gln105 interaction, anchors the sulfonamide core within the pterin‐binding subsite and mirrors the interaction pattern observed for both SMX and the co‐crystallized substrate analog at these same residues.

Beyond these classical hydrogen bonds, the complex is further stabilized by a distinct set of electrostatic pi–cation interactions, in which the positively charged guanidinium groups of Arg52 and Arg239 engage the pi–electron systems of the ligand’s aromatic rings, alongside a pi–sulfur interaction with Phe172 involving the sulfonamide sulfur atom. Additional hydrophobic stabilization arises from alkyl and pi‐alkyl contacts with Arg204, Lys207, Lys203, Met128, and Ala199, reflecting close packing of the compound’s aromatic and chalcone moieties against the arginine‐ and lysine‐rich loop lining the active site entrance.

Taken together, this multilayered interaction network, combining conventional hydrogen bonding at Gln105/Arg239 with a rich set of pi‐cation, pi‐sulfur, and pi‐alkyl contacts, provides a plausible structural rationale for **12e**’s good antibacterial activity against the Gram‐positive strains *S. aureus* and *S. pyogenes*.

The binding affinities, hydrogen‐bonding patterns, and residue interactions of the synthesized compounds are summarized in Table S2. The 2D and 3D binding interactions of the co‐crystallized ligand, **12e,** and **SMX** are illustrated in Figures 10, 11, 12, respectively, while the corresponding diagrams for the other compounds are provided in Figures S40–S48.

**FIGURE 11:**
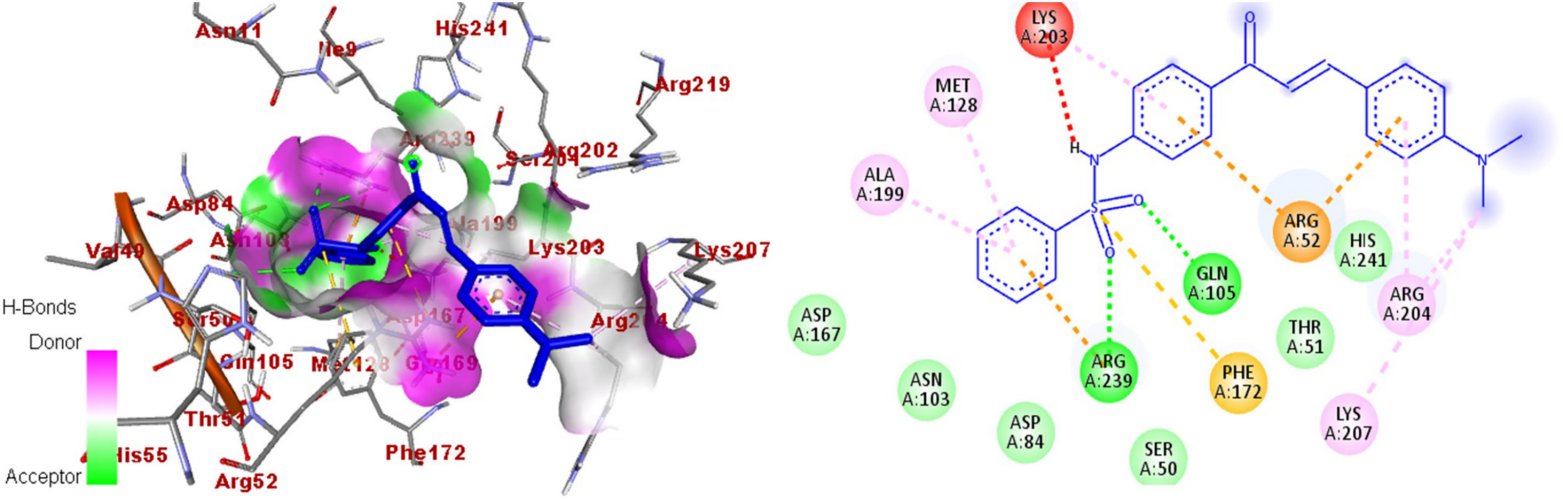
3D (left) and 2D (right) representations of the binding interactions of compound **12e** against *S. aureus* DHPS (PDB ID: 1DA4).

**FIGURE 12:**
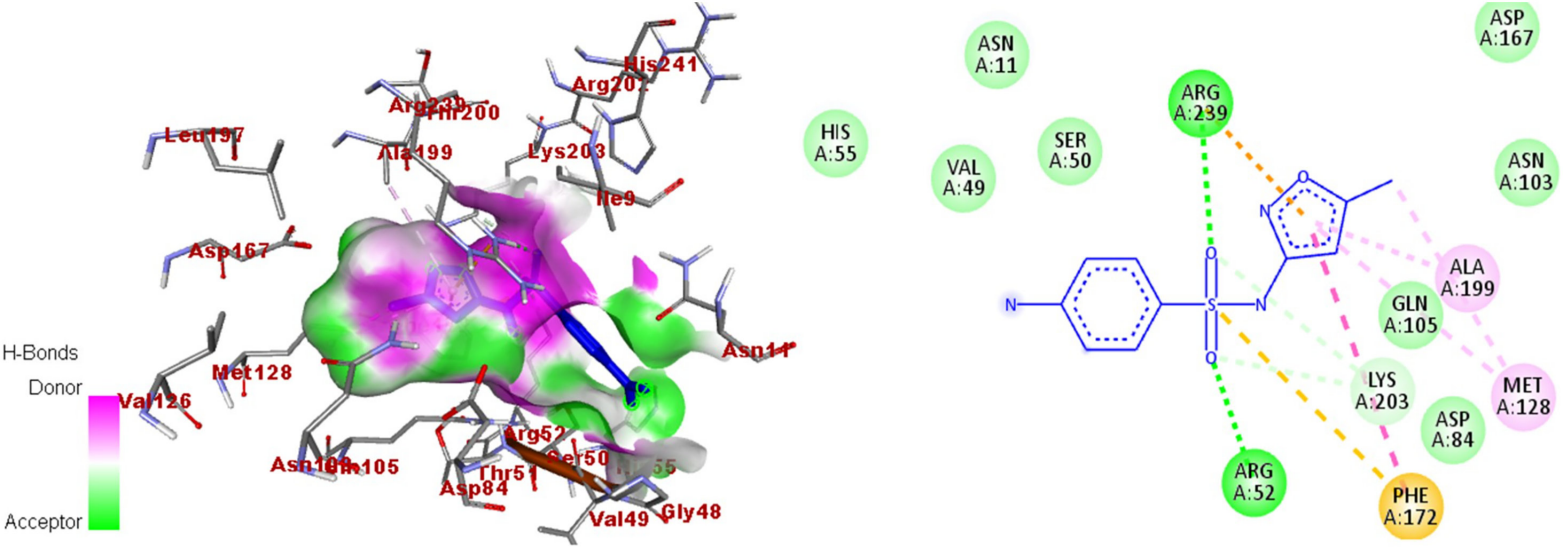
3D (left) and 2D (right) representations of the binding interactions of compound **SMX** against *S. aureus* DHPS (PDB ID: 1DA4).

Although **12a–h** showed more favorable docking scores than SMX against both *E. coli* DHPS (1AJ0: −8.5 to −8.9 kcal/mol vs. −7.1) and *S. aureus* DHPS (1AD4: −6.3 to −7.5 kcal/mol vs. −6.6), this was not mirrored in whole‐cell activity, where ZOI values (6.3–14.7 mm) remained well below SMX (23.6–24.7 mm). Docking scores reflect intrinsic ligand‐active‐site complementarity but do not capture a compound’s ability to cross the bacterial envelope or avoid efflux. The higher molecular weight and lipophilicity of **12a–h** (395–468 g/mol, iLogP 2.91–3.60) relative to the small, polar SMX (253 g/mol; Table S6), likely limit membrane penetration, particularly across the Gram‐negative outer membrane, consistent with the more lipophilic, methoxy‐substituted derivatives outperforming their analogs against Gram‐negative strains [38].

At the residue level, the most active compounds still engage the key pABA‐mimetic anchoring residues: **12b** (most active against *E. coli*) forms hydrogen bonds with Gly191, Ser222, Arg255, Pro145, Thr147, and Arg63, plus π‐interactions with Phe190, mirroring the validated sulfanilamide pose. **12e** (most active against *S. aureus*) forms bidentate hydrogen bonds with Arg239 and a further bond with Gln105, reinforced by π‐cation and π‐sulfur contacts, matching the native ligand’s binding mode. This supports genuine target engagement, while indicating that permeability and efflux avoidance, rather than binding affinity, are the more likely determinants of the moderate in vitro potency and the priority for future optimization [39, 40].

#### 2.5.3 Against Human Myeloperoxidase (MPO) (PDB ID: 1DNU)

Myeloperoxidase (MPO) is a heme enzyme with a dual role in physiology. It contributes to host defense by generating hypochlorous acid (HOCl) from hydrogen peroxide and chloride ions, aiding in pathogen elimination. However, excessive MPO activity promotes the overproduction of ROS, leading to oxidative damage of biomolecules. Elevated MPO levels are strongly associated with cardiovascular and neurodegenerative diseases, where oxidative stress and inflammation are key drivers [41]. Therefore, MPO inhibition has emerged as a promising strategy for mitigating oxidative stress‐mediated pathological conditions, making it a relevant target for the evaluation of antioxidant and anti‐inflammatory agents [42].

The molecular docking analysis of the synthesized chalcone‐sulfonamide hybrids (**12a–12h**) against MPO (PDB ID: 1DNU) revealed strong binding affinities ranging from −8.8 to −9.8 kcal/mol, substantially higher than that of the reference compound ascorbic acid (AA, −6.1 kcal/mol) (Table 4). This marked difference in binding energy indicates that the chalcone‐sulfonamide scaffold engages the MPO active site considerably more efficiently than the small, polar reference antioxidant, likely reflecting the larger surface area and greater conformational adaptability of the hybrid molecules within the heme‐lined distal cavity of the enzyme. Across the series, Arg333 emerged as the most consistently engaged hydrogen‐bonding residue, forming direct contacts with nearly all derivatives, while His336, Arg239, Arg424, and Gln91 recurred as secondary anchoring points depending on the substitution pattern on the chalcone ring. This convergence on a shared subset of active‐site residues suggests that the compounds occupy a common binding orientation within the MPO distal heme cavity. Additional stabilization throughout the series arose from extensive hydrophobic and van der Waals contacts with Phe332, Phe407, Leu417, Leu420, Gly90, Asp94, and His95, residues that line the hydrophobic channel leading to the heme edge, reinforcing that binding is driven by a combination of directional hydrogen bonding at the entrance residues and nonpolar packing deeper within the cavity (Table S3).

A detailed analysis of the interaction profile of compound **12e**, the most potent antioxidant in vitro (Table 2), showed a strong binding affinity of −9.6 kcal/mol within the MPO active site, significantly outperforming ascorbic acid (−6.1 kcal/mol); Table 4. Docking analysis revealed that **12e** is deeply embedded in the distal heme cavity, with its chalcone moiety aligned along the hydrophobic channel and the sulfonamide group oriented toward the arginine‐rich entrance of the binding pocket. This configuration enables simultaneous interactions with both polar and nonpolar residues.

A robust hydrogen‐bonding network was observed with Arg239, Arg333, and His336, supported by additional C–H interactions with Thr238. This multipoint anchoring, particularly the well‐oriented His336 interaction, enhances binding stability and distinguishes 12e from analogs that rely on fewer hydrogen bonds.

Further stabilization arises from electrostatic and hydrophobic interactions with residues such as Asp94, His95, Phe366, Leu417, Arg239, and Arg333, along with extensive van der Waals contacts involving residues lining the heme channel. These interactions strongly enclose the ligand, suggesting effective steric and electronic interference with substrate access to the heme iron, consistent with MPO inhibition.

**FIGURE 13:**
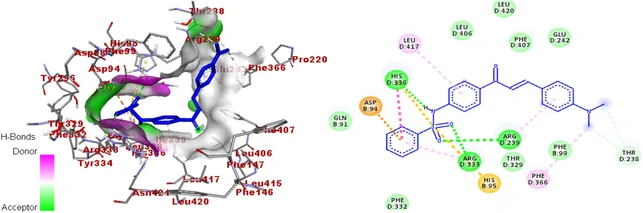
3D (left) and 2D (right) representations of the binding interactions of compound **12e** against MPO (PDB ID: 1DNU).

**FIGURE 14:**
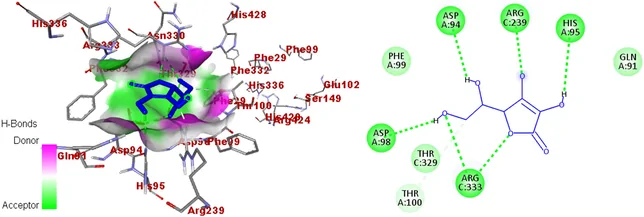
3D (left) and 2D (right) representations of the binding interactions of ascorbic acid against MPO (PDB ID: 1DNU).

In contrast, ascorbic acid exhibited a more limited interaction profile, with fewer stabilizing contacts and weaker binding affinity. Overall, the dense, multilayered interaction network of 12e provides a clear structural basis for its superior antioxidant activity and supports MPO inhibition as a key mechanism. The binding affinities, hydrogen‐bonding patterns, and residue interactions of the synthesized compounds are summarized in Table S3. The 2D and 3D binding interactions of compound **12e** and ascorbic acid are illustrated in Figures 13 and 14, while the corresponding diagrams for the other compounds are provided in Figures S49–S57.

#### 2.5.4 Against COX‐2 (PDB ID: 5IKR)

Cyclooxygenase‐2 (COX‐2) was selected as a molecular target due to its central role in inflammatory processes. COX‐2 is an inducible enzyme responsible for the conversion of arachidonic acid into prostaglandins, which are key mediators of inflammation, pain, and fever [43, 44]. Overexpression of COX‐2 is strongly associated with various inflammatory conditions, making it an important therapeutic target for anti‐inflammatory drug development [45]. Nonsteroidal anti‐inflammatory drugs (NSAIDs) exert their pharmacological effects by binding to the COX‐2 active site, thereby inhibiting prostaglandin biosynthesis and reducing inflammatory responses [46]. Therefore, investigation of ligand interactions within the COX‐2 binding pocket is essential for the identification and development of effective anti‐inflammatory agents targeting pain and inflammation.

The anti‐inflammatory potential of compounds **12a–12h** was assessed through molecular docking studies against COX‐2 (PDB ID: 5IKR). In this study, the docking analysis for Cyclooxygenase‐2 (COX‐2, PDB ID: 5IKR) was used to complement the anti‐inflammatory results and to provide a mechanistic explanation for the activity of the synthesized chalcone‐sulfonamide hybrids. Before evaluating the target compounds, the docking protocol was validated by re‐docking the co‐crystallized mefenamic acid ligand into the COX‐2 active site. The re‐docked pose (Figure 6C) showed good agreement with the crystallographic orientation, with an RMSD of 0.59 Å, well below the commonly accepted cutoff of 2.0 Å, confirming that the docking settings were reliable for predicting ligand binding within the COX‐2 active site.

**FIGURE 15:**
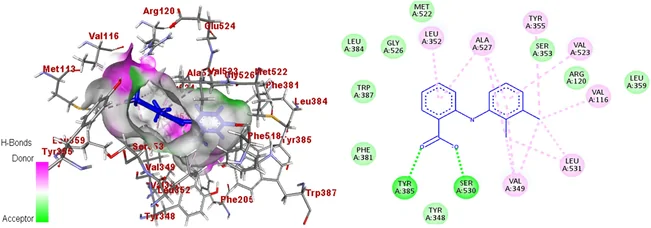
3D (left) and 2D (right) representations of the binding interactions of mefenamic acid against COX‐2 (PDB ID: 5IKR).

The binding mode analysis of mefenamic acid (Figure 15) reveals the critical role of hydrogen–bonding interactions involving the Ser530 and Tyr385 residues. In addition, electrostatic and hydrophobic interactions with Ala527, Val349, Leu531, Val116, Thr355, Leu352, and Val523, together with numerous van der Waals contacts, further stabilize the ligand‐enzyme complex. These findings are consistent with previous studies, which have emphasized the importance of these key residues in the active site of COX‐2 [47].

The validated protocol was then applied to the synthesized compounds **12a–h**. The docking scores against 5IKR ranged from −8.4 to −9.5 kcal/mol, indicating favorable binding for all derivatives, comparable to or exceeding that of the reference compound diclofenac sodium (−8.5 kcal/mol) (Table 4). Among the series, compounds **12e**, **12g**, and **12h** exhibited the most favorable binding energies, in agreement with their superior in vitro anti‐inflammatory activities.

All compounds exhibited hydrogen–bonding interactions with at least one key residue within the enzyme’s active site, while additional hydrophobic contacts contributed to overall complex stabilization depending on the ligand conformation (Table S4). A detailed analysis of the interaction profile of compound **12g**, identified as the most balanced active derivative across both assays, reveals extensive engagement with several critical active‐site residues. Specifically, the ligand formed a conventional hydrogen bond with Ser530, mirroring the key hydrogen‐bonding residue identified for mefenamic acid in the validated re‐docking pose and supporting a shared binding orientation between the reference ligand and **12g**. A pi–cation interaction with Arg120 further anchored the ligand within the polar entrance of the active site, while pi‐sigma contacts with Val116, Val349, and Ala527 contributed additional directional stabilization. Hydrophobic character was reinforced through a pi–sulfur interaction with Met522, a pi–pi T–shaped interaction with Trp387, and an amide–pi stacked interaction between Gly526/Ala527 and the ligand core. Alkyl and pi‐alkyl contacts with Val89, Tyr115, Leu352, and Leu531 completed the stabilization network. The convergence of these interactions around Ser530, Arg120, and Trp387, residues also implicated in mefenamic acid recognition, provides a structural rationale for the favorable binding affinity calculated for **12g** (−9.4 kcal/mol) and supports its enhanced in vitro anti‐inflammatory performance relative to diclofenac in the anti‐proteinase assay.

The binding affinities, hydrogen‐bonding patterns, and residue interactions of the synthesized compounds are summarized in Table S4. The 2D and 3D binding interactions of mefenamic acid, **12g,** and diclofenac are illustrated in Figures 15, 16, 17, while the corresponding diagrams for the other compounds are provided in Figures S58–S66.

**FIGURE 16:**
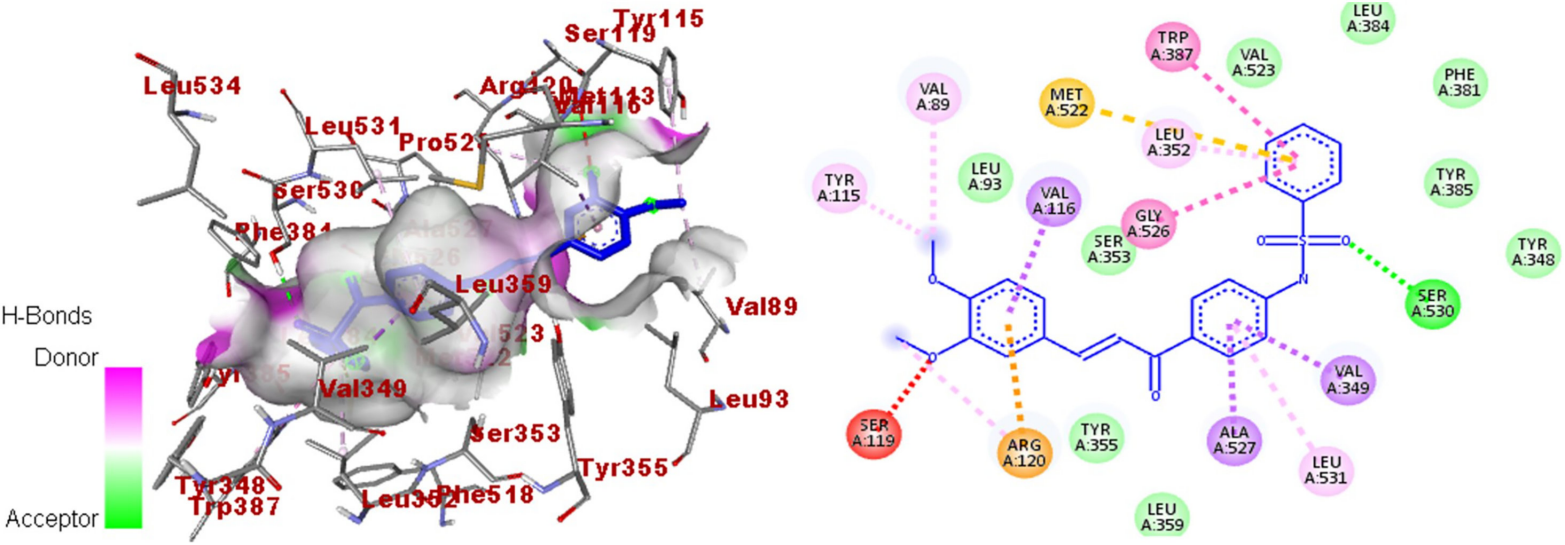
3D (left) and 2D (right) representations of the binding interactions of compound **12g** against COX‐2 (PDB ID: 5IKR).

**FIGURE 17:**
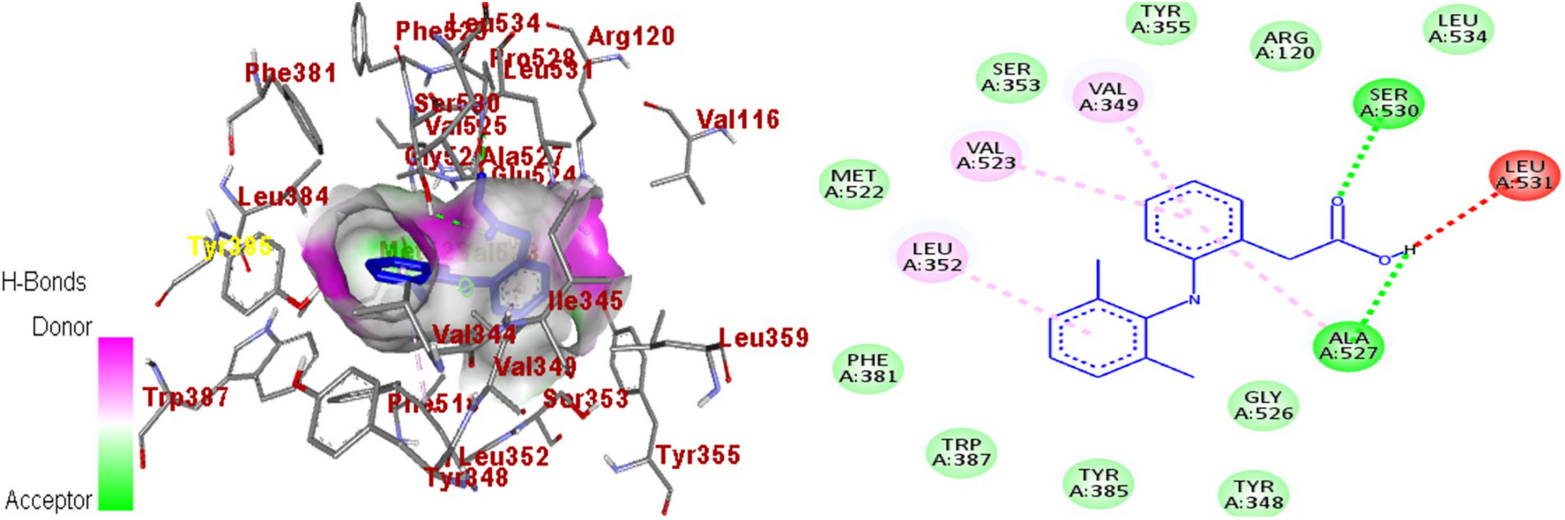
3D (left) and 2D (right) representations of the binding interactions of diclofenac against COX‐2 (PDB ID: 5IKR).

#### 2.5.5 Against Topoisomerase *IIα* (PDB ID: 4FM9)

Topoisomerase *IIα* is a critical molecular target owing to its central role in DNA replication, transcription, and cell division. The enzyme regulates DNA topology by inducing transient double‐stranded breaks, facilitating strand passage and subsequent re‐ligation to maintain genomic integrity. Given its essential function in rapidly proliferating cells, Topoisomerase *IIα* is highly expressed in many cancers and represents a well‐validated target for anticancer therapy. Consequently, inhibiting this enzyme can disrupt DNA processing and trigger cytotoxic effects, underscoring its importance in anticancer drug development [48]. Inhibition of this enzyme leads to the accumulation of DNA damage, resulting in cell cycle arrest and apoptosis in cancer cells. Clinically used anticancer agents, such as etoposide, exert their activity by stabilizing the enzyme‐DNA cleavage complex and preventing DNA re‐ligation [49]. Therefore, investigation of ligand interactions within the Topoisomerase *IIα* binding site is essential for identifying potential anticancer candidates. Molecular docking studies of the synthesized chalcone‐sulfonamide hybrids against topoisomerase *IIα* (PDB ID: 4FM9) revealed significant binding affinities, ranging from −8.8 to −9.8 kcal/mol, comparable to the reference etoposide (−9.7 kcal/mol), Table 4. Within this series, compounds **12b** and **12e** demonstrated the most favorable binding energies (−9.8 kcal/mol each), equaling that of etoposide.

**FIGURE 18:**
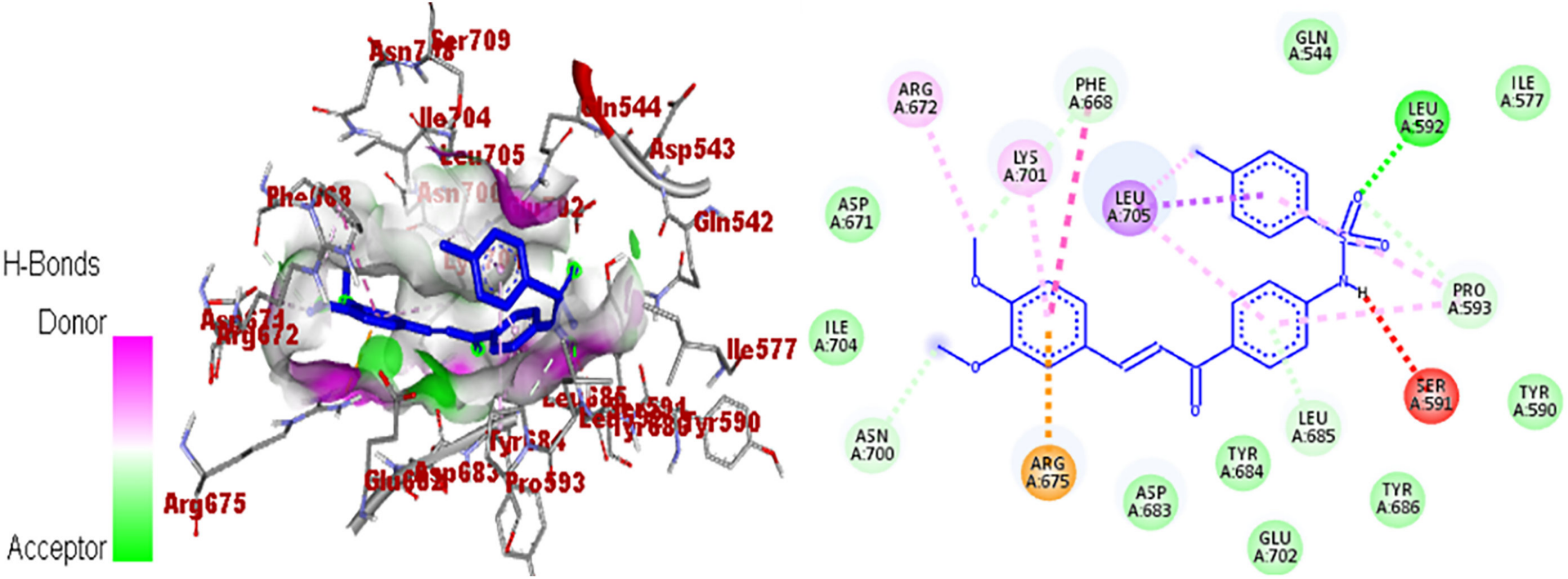
3D (left) and 2D (right) representations of the binding interactions of compound **12b** against Topoisomerase *IIα* DHPS (PDB ID: 4FM9).

**FIGURE 19:**
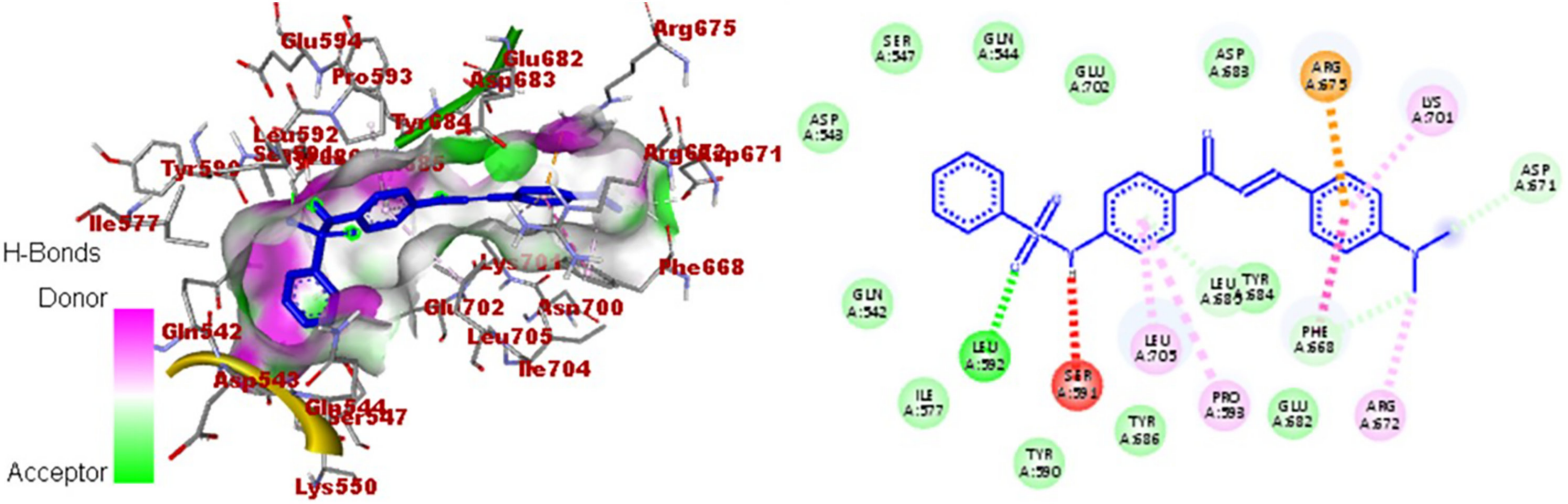
3D (left) and 2D (right) representations of the binding interactions of compound **12e** against Topoisomerase *IIα* DHPS (PDB ID: 4FM9).

**FIGURE 20:**
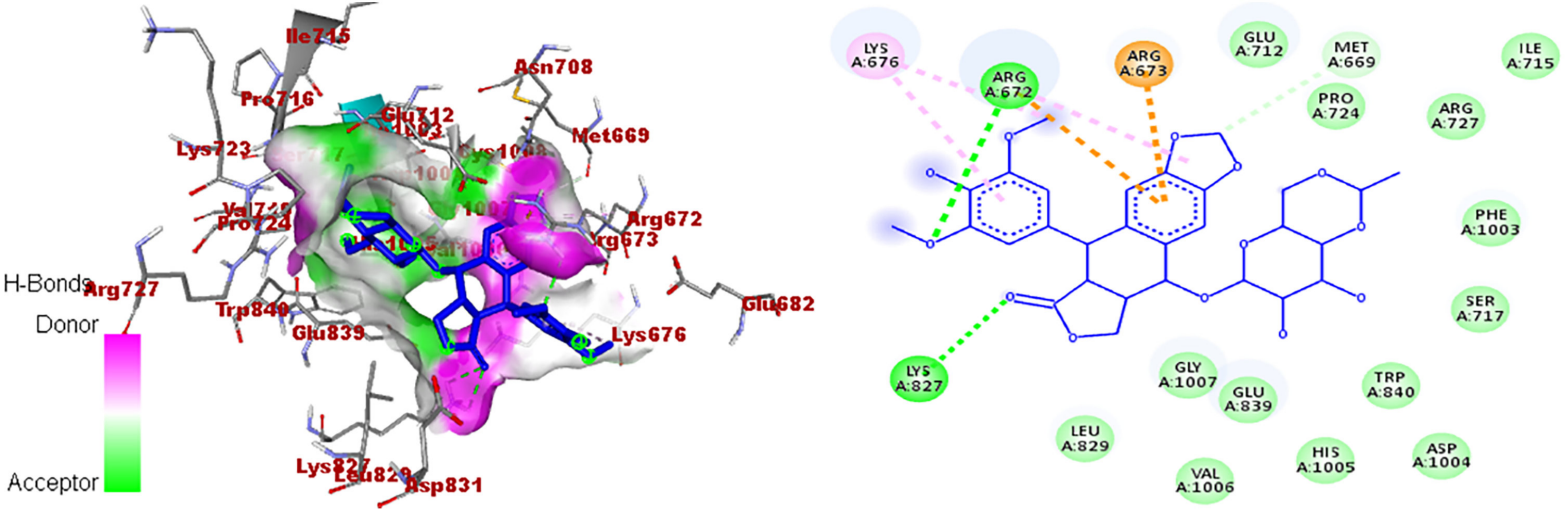
3D (left) and 2D (right) representations of the binding interactions of etoposide against Topoisomerase *IIα* DHPS (PDB ID: 4FM9).

Structural analyses showed that compounds **12b**, **12d**, **12e**, and **12g** adopt a common binding mode, forming key hydrogen bonds with Leu592 and Leu685, while also engaging in extensive hydrophobic and electrostatic interactions with residues such as Arg675, Phe668, Pro593, and Leu705, which likely contribute to complex stabilization along with van der Waals interactions. On the other hand, compounds **12a**, **12c**, **12f**, and **12h** exhibited interaction patterns similar to etoposide, involving residues Arg672, Arg673, Glu712, and Glu839. Notably, **12c** formed multiple hydrogen bonds with Arg672, Arg673, Asp1004, Ile715, and Glu712, reflecting a robust and diverse binding profile. Overall, the combination of high binding affinity and extensive hydrogen bonding observed for compounds **12b** and **12e** underscores their potential as promising predicted lead candidates for further anticancer development. The binding affinities, hydrogen‐bonding patterns, and residue interactions of the synthesized compounds are summarized in Table S5. The 2D and 3D binding interactions of compound **12b, 12e** and etoposide are illustrated in Figures 18, 19, 20, while the corresponding diagrams for the other compounds are provided in Figures S67–S75.

### 2.6 Drug‐Likeness, ADMET, and Toxicity Predictions

#### 2.6.1 Drug‐Likeness Predictions

SwissADME‐based drug‐likeness evaluation (Table S6) showed that all synthesized compounds (**12a–h**) fully comply with Lipinski’s Rule of Five, with no rule violations, indicating their suitability as orally active drug candidates [50]. The molecular weights of compounds **12a–h** (395.45–467.53 g/mol) fall within the acceptable Lipinski limit and, although higher than that of the reference drug sulfamethoxazole (**SMZ**, 253.28 g/mol), remain compatible with good pharmacokinetic behavior. All compounds possess a single hydrogen bond donor and 3–6 hydrogen bond acceptors, values comparable to **SMZ** and favorable for maintaining an appropriate balance between polarity and membrane permeability. The number of rotatable bonds (**6–9**) suggests moderate molecular flexibility, which may support effective target binding while remaining within recommended limits. Topological polar surface area (TPSA) values ranged from 71.62 to 99.31 Å^2^, well below the critical threshold for oral absorption and comparable to **SMZ** (106.60 Å^2^). Lipophilicity values (*i* Log *P* = 2.91–3.60) indicate moderate lipophilicity, higher than SMZ but still within the optimal range, potentially enhancing membrane permeability without compromising solubility. Overall, these results suggest that compounds **12a–h** possess well‐balanced physicochemical properties and promising oral drug‐likeness profiles relative to the standard drug **SMZ**.

#### 2.6.2 ADME Predictions

The ADME properties of compounds **12a–h** were predicted using the SwissADME computational platforms, which provide reliable estimates of key pharmacokinetic parameters relevant to early‐stage drug development [51, 52]. These tools are particularly valuable in medicinal chemistry for evaluating absorption, distribution, metabolism, and excretion characteristics of small molecules prior to experimental validation. All synthesized compounds (**12a–h**) were predicted to exhibit high gastrointestinal absorption, comparable to the standard drug sulfamethoxazole (**SMZ**), indicating favorable oral bioavailability (Table S7). Such a profile is essential for orally administered therapeutics and supports their potential systemic efficacy [53]. Furthermore, none of the compounds were predicted to cross the blood‐brain barrier, suggesting limited central nervous system exposure and a potentially reduced risk of neurological side effects. Cytochrome P450 inhibition analysis revealed that all synthesized compounds inhibit CYP2C19, CYP2C9, and CYP3A4, whereas **SMZ** showed no inhibitory activity toward these isoforms. In addition, compounds **12d** and **12e** were predicted to inhibit CYP1A2. These findings indicate possible drug–drug interaction liabilities that should be carefully considered during further optimization and development stages [54]. Skin permeability predictions (LogKp) suggested moderate permeability for compounds **12a–h** (−5.40 to −6.13 cm/s), higher than that of **SMZ** (−7.21 cm/s), indicating comparatively enhanced dermal penetration. Moreover, none of the compounds were identified as P‐glycoprotein substrates. Overall, the ADME predictions support the drug‐like nature of the synthesized compounds and justify their further pharmacological investigation.

#### 2.6.3 Toxicity Prediction

The in silico toxicity evaluation revealed that the synthesized compounds (**12a–h**) generally possess more favorable safety profiles than the reference drug **SMZ** (Table S8). Most of the tested compounds were predicted to have moderate hepatotoxic effects; however, compounds **12d** and **12e** showed no associated hepatotoxic risk. Notably, none of the synthesized compounds exhibited carcinogenic potential, whereas **SMZ** was predicted to present a moderate risk of carcinogenicity. Immunotoxic effects were predicted only for compounds **12c** and **12h**, while **SMZ** showed no immunotoxic concern. Importantly, all synthesized compounds were predicted to be nonmutagenic and noncytotoxic, further supporting their overall safety. The predicted acute toxicity results reinforced these findings: all synthesized compounds exhibited LD_50_ values of 6000 mg/kg, substantially higher than that of SMZ (2300 mg/kg), indicating a broader margin of safety. Accordingly, the synthesized compounds were categorized under toxicity class 6, compared to class 5 for SMZ, reflecting a lower predicted risk of acute toxicity. However, it should be noted that these toxicity profiles are based on computational prediction and require experimental validation before definitive conclusions on the safety of the synthesized compounds can be drawn. Taken together, the in silico toxicity predictions support the promising therapeutic safety profile of this compound series.

## 3 Conclusion

In conclusion, chalcone‐sulfonamide hybrids **12a–h** were successfully synthesized through the Claisen–Schmidt condensation approach and thoroughly characterized by NMR spectroscopy. The synthesized derivatives demonstrated moderate to good antibacterial activity against clinically important Gram‐negative (*E. coli*, *P. aeruginosa*) and Gram‐positive (*S. aureus*, *S. pyogenes*). Among them, compound **12b** showed notable potency against *E. coli*, while **12e** emerged as the most active candidate against *S. aureus*. MIC. In addition, all compounds exhibited significant DPPH radical‐scavenging activity, with IC_50_ values ranging from 5.2 to 11.8 μg m/L, suggesting their potential to mitigate infection‐associated oxidative stress. Furthermore, compound **12e** showed the strongest anti‐protein denaturation activity, while **12g** and **12h** exhibited strong anti‐proteinase activity among the synthesized derivatives, making them the most promising lead compounds. Molecular docking investigations indicated strong binding affinities (−8.3 to −10.3 kcal/mol) toward bacterial DHPS (1AJ0, 1AD4), myeloperoxidase (1DNU), COX‐2 (5IKR), and topoisomerase *IIα* (4FM9). These affinities were superior to those of reference drugs such as sulfamethoxazole, ascorbic acid, and diclofenac, aligning well with the in vitro findings and highlighting DHPS, MPO, and COX‐2 as probable biological targets. Furthermore, SwissADME and ProTox predictions supported favorable pharmacokinetic and safety profiles, including compliance with Lipinski’s rule of five, high gastrointestinal absorption, lack of blood‐brain barrier permeability, and low predicted toxicity (LD_50_  > 6000 mg/kg), though these computational findings warrant experimental confirmation. Generally, this hybrid scaffold effectively combines antibacterial, antioxidant, and anti‐inflammatory properties, offering a promising strategy to address AMR.

## 4 Materials and Methods

All solvents and reagents were purchased from commercial suppliers and used as received, without further purification. Melting points were determined in open capillary tubes using a digital melting point apparatus and are reported in degrees Celsius. Reaction progress was monitored by thin‐layer chromatography (TLC) on pre‐coated plates, and spots were visualized under UV light at 254 nm. The synthesized compounds were characterized based on their physical properties and spectral analyses. 1D and 2D ^1^H and ^13^C NMR spectra were recorded on a Bruker Avance 400 MHz NMR spectrometer using DMSO‐d_6_ as the solvent, and chemical shifts reported in *δ* (ppm) were referenced to the resonances of the solvent at 2.49 ppm (^1^H) and 39.5 ppm (^13^C).

### 4.1 Synthesis of Ketone Sulfonamides, 10a–b

N‐(4‐Acetylphenyl)benzene sulfonamide (**10a**) and N‐(4‐acetylphenyl)‐4‐methylbenzene sulfonamide (**10b**) were synthesized following previously reported procedures [3]. Briefly, p‐aminoacetophenone (50 mmol) was reacted with either benzenesulfonyl chloride or 4‐methylbenzenesulfonyl chloride (50 mmol) in methanol (50 mL), while pyridine (5 mL) was added dropwise. The reaction mixture was refluxed for 24 h. Upon completion, the mixture was poured onto crushed ice and acidified using 10% HCl. The resulting precipitate was collected by filtration, washed sequentially with 2% NaHCO_3_ and water, and dried to afford the desired sulfonamides.

#### 4.1.1 *N*‐(4‐acetylphenyl)‐4‐methylbenzenesulfonamide (10a)

White solid; Yield 78.3% (11.3 g); mp 190–191 °C (Lit. 188–189 °C [3]); Rf 0.47 (*n*‐hexane: ethyl acetate, 1:1). ^1^H NMR (400 MHz, DMSO‐d_6_): *δ* 10.79 (s, 1H, NH); 7.82 (m, 2H, H‐3, 5); 7.71 (m, 2H, H‐2′, 6′); 7.34 m, 2H, H‐3′, 5′); 7.20 (m, 2H, H‐2, 6); 2.44 (s, 3H, H‐8); 2.30 (s, 3H, 4′‐Me). ^13^C NMR (100 MHz, DMSO‐d_6_): *δ* 196.4 (C═O); 143.7 (C‐4′); 142.3 (C‐1); 136.4 (C‐1′); 131.9 (C‐4); 129.84 (C‐3′, 5′); 129.77 (C‐3, 5); 126.7 (C‐2′, 6′); 117.8 (C‐2, 6); 26.3 (C‐8); 20.9 (C‐4′‐Me).

#### 4.1.2 *N*‐(4‐acetylphenyl)benzenesulfonamide (10b)

White solid; Yield 66.7% (9.2 g); mp 140–142  °C (Lit. 141–142 °C [3]); Rf 0.55 (*n*‐hexane: ethyl acetate, 1:1) . ^1^H NMR (400 MHz, DMSO‐d_6_): *δ* 10.87 (s, 1H, NH); 7.83 (m, 2H, H‐2′, 6′); 7.82 (m, 2H, H‐3, 5); 7.61 (m, 1H, H‐4′); 7.55 (m, 2H, H‐3′, 5′); 7.20 (m, 2H, H‐2, 6); 2.44 (s, 3H, H‐8). ^13^C NMR (100 MHz, DMSO‐d_6_): *δ* 196.4 (C═O); 142.2 (C‐1); 139.3 (C‐1′); 133.2 (C‐4′); 132.0 (C‐4); 129.8 (C‐3, 5); 129.4 (C‐3′, 5′); 126.7 (C‐2′, 6′); 118.0 (C‐2, 6); 26.4 (C‐8).

### 4.2 General Procedure for Synthesis of Chalcones Sulfonamides (12a–h)

The sulfonamide chalcone derivatives (**12a–h**) were prepared via a Claisen–Schmidt condensation reaction, employing potassium hydroxide (30% KOH) as the base and methanol as the solvent, following a previously reported procedure with minor modifications [24]. In a typical reaction, 7 mmol of the ketone **10a–b** was dissolved in 50 mL of methanol. To this solution, 7 mmol of the appropriate aldehyde (**11a–e**) was added, followed by the dropwise addition of 15 mmol of 30% KOH solution. The reaction mixture was stirred at room temperature for 24 h and monitored by TLC on pre‐coated silica gel plates, with visualization under UV light. Upon completion, the reaction mixture was quenched in ice‐cold water and neutralized with dilu. HCl. The resulting crude chalcone precipitate was collected by filtration, washed with chilled ethanol, dried, and recrystallized from methanol.

#### 4.2.1 (E)‐*N*‐(4‐(3‐(2,4‐dimethoxyphenyl)acryloyl)phenyl)‐4‐methylbenzenesulfonamide (12a)

**Y**ellow solid; Yield 77.5% (2.3 g); mp 120–122  °C; Rf 0.53 53 (*n*‐hexane: ethyl acetate, 3:2). ^1^H NMR (400 MHz, DMSO‐d_6_): *δ* 10.82 (s, 1H, NH); 7.99 (m, 2H, H‐3, 5); 7.93 (d, *J* = 15.7 Hz, 1H, H‐9); 7.84 (d, *J* = 8.4 Hz, 1H, H‐6″); 7.73 (m, 2H, H‐2′, 6′); 7.67 (d, *J* = 15.7 Hz, 1H, H‐8); 7.35 (m, 2H, H‐3′, 5′); 7.25 (m, 2H, H‐2, 6); 6.61 (m, 1H, H‐3″); 6.59 (m, 1H, H‐5″); 3.87 (s, 3H, 2″‐OMe); 3.81 (s, 3H, 4″‐OMe); 2.30 (s, 3H, 4′‐Me). ^13^C NMR (100 MHz, DMSO‐d_6_): *δ* 187.5 (C═O); 163.0 (C‐4″); 159.9 (C‐2″); 143.7 (C‐4′); 142.1 (C‐1); 138.3 (C‐9); 136.5 (C‐1′); 133.0 (C‐4); 130.0 (C‐6″); 129.9 (C‐3, 5); 129.8 (C‐3′, 5′); 126.8 (C‐2′, 6′); 118.8 (C‐8); 118.0 (C‐2, 6); 115.9 (C‐1″); 106.3 (C‐5″); 98.3 (C‐3″); 55.8 (2″‐OMe); 55.5 (4″‐OMe); 20.9 (4′‐Me).

#### 4.2.2 (*E*)‐*N*‐(4‐(3‐(3,4‐dimethoxyphenyl)acryloyl)phenyl)‐4‐methylbenzenesulfonamide (12b)

Yellow solid; Yield 78.8% (2.4 g); mp 179–181  °C; Rf 0.55 (*n*‐hexane: ethyl acetate, 3:2). ^1^H NMR (400 MHz, DMSO‐d_6_): *δ* 10.82 (s, 1H, NH); 8.04 (m, 2H, H‐3, 5); 7.73 (m, 2H, H‐2′, 6′); 7.73 (d, *J* = 15.5 Hz, 1H, H‐8); 7.64 (d, *J* = 15.5 Hz, 1H, H‐9); 7.49 (d, *J* = 1.9 Hz, 1H, H‐2″); 7.35 (m, 2H, H‐3′, 5′); 7.33 (dd, *J* = 8.4/1.9 Hz, 1H, H‐6″); 7.25 (m, 2H, H‐2, 6); 6.99 (d, *J* = 8.4, 1H, H‐5″); 3.84 (s, 3H, 3″‐OMe); 3.79 (s, 3H, 4″‐OMe); 2.31 (s, 3H, 4′‐Me).^13^C NMR (100 MHz, DMSO‐d_6_): *δ* 187.4 (C═O); 151.2 (C‐4″); 149.0 (C‐3″); 144.0 (C‐9); 143.7 (C‐4′); 142.2 (C‐1); 136.4 (C‐1′); 132.8 (C‐4); 130.1 (C‐3, 5); 129.9 (C‐3′, 5′); 127.5 (C‐1″); 126.7 (C‐2′, 6′); 123.9 (C‐6″); 119.3 (C‐8); 117.9 (C‐2, 6); 111.5 (C‐5″); 110.6 (C‐2″); 55.7 (3″‐OMe); 55.6 (4″‐OMe); 20.9 (4′‐Me).

#### 4.2.3 (*E*)‐4‐methyl‐N‐(4‐(3‐(3,4,5‐trimethoxyphenyl)acryloyl)phenyl)benzenesulfonamide (12c)

Yellow solid; Yield 74.5% (2.4 g); mp 190–192 °C; Rf 0.5 (*n*‐hexane: ethyl acetate, 3:2). ^1^H NMR (400 MHz, DMSO‐d_6_): *δ* 10.85 (s, 1H, NH); 8.06 (m, 2H, H‐3, 5); 7.80 (d, *J* = 15.5 Hz, 1H, H‐8); 7.74 (m, 2H, H‐2′, 6′); 7.64 (d, *J* = 15.5 Hz, 1H, H‐ 9); 7.36 (m, 2H, H‐ 3′, 5′); 7.26 (m, 2H, H‐2, 6); 7.18 (s, 2H, H‐2″, 6″); 3.84 (s, 6H, 3″, 5″‐OMe); 3.70 (s, 3H, 4″‐OMe); 2.31 (s, 3H, 4′‐Me). ^13^C NMR (100 MHz, DMSO‐d_6_): *δ* 187.4 (C═O); 153.1 (C‐3″, 5″); 144.0 (C‐9); 143.7 (C‐4′); 142.4 (C‐1); 139.7 (C‐4″); 136.4 (C‐1′); 132.6 (C‐4); 130.3 (C‐1″); 130.2 (C‐3, 5); 129.9 (C‐3′, 5′); 126.8 (C‐2′, 6′); 121.0 (C‐8); 117.9 (C‐2, 6); 106.4 (C‐2″, 6″); 60.1 (C‐4″‐OMe); 56.1 (C‐3″, 5″‐OMe); 20.9 (4′‐Me).

#### 4.2.4 (*E*)‐*N*‐(4‐(3‐(4‐fluorophenyl)acryloyl)phenyl)‐4‐methylbenzenesulfonamide (12d)

Yellow solid; Yield 83% (2.3 g); mp 202–204  °C; Rf 0.7 (*n*‐hexane: ethyl acetate, 3:2). ^1^H NMR (400 MHz, DMSO‐d_6_): *δ* 10.88 (s, 1H, NH); 8.04 (m, 2H, H‐3,5); 7.91 (m, 2H, H‐2″, 6″); 7.81 (d, *J* = 15.6 Hz, 1H, H‐8); 7.74 (m, 2H, H‐2′, 6′); 7.67 (d, *J* = 15.6 Hz, 1H, H‐9); 7.35 (m, 2H, H‐3′, 5′); 7.26 (m, 2H, H‐3″, 5″); 7.25 (m, 2H, H‐2, 6); 2.30 (s, 3H, 4′‐Me).^13^C NMR (100 MHz, DMSO‐d_6_): *δ* 187.4 (C = O); 163.3 (sd, J(F,C) = 249, C‐4″); 143.7 (C‐4′); 142.6 (C‐1); 142.2 (C‐9); 136.5 (C‐1′); 132.4 (C‐4); 131.4 (sd, J(F,C) = 3.1, C‐1″); 131.1 (dd, J(F,C) = 8.9, C‐2″, 6″); 130.2 (C‐3, 5); 129.8 (C‐3′, 5′); 126.8 (C‐2′, 6′); 121.7 (C‐8); 117.9 C‐2, 6); 115.8 (dd, J(F,C) = 21.7, C‐3″,5″); 20.9 (4′‐Me). ^19^F NMR (376 MHz, DMSO‐d_6_) *δ* −109.7.

#### 4.2.5 (*E*)‐*N*‐(4‐(3‐(4‐(dimethylamino)phenyl)acryloyl)phenyl)benzenesulfonamide (12e)

Orange solid; Yield 73% (2.1 g); mp 132–134  °C; Rf 0.48 (*n*‐hexane: ethyl acetate, 3:2). ^1^H NMR (400 MHz, DMSO‐d_6_): *δ* 10.85 (s, 1H, NH); 7.99 (m, 2H, H‐3, 5); 7.83 (m, 2H, H‐2′, 6′); 7.65 (m, 2H, H‐2″, 6″); 7.61 (m, 1H, H‐9); 7.61 (m, 1H, H‐4′); 7.56 (m, 2H, H‐3′, 5′); 7.53 (d, *J* = 15.4 Hz, 1H, H‐8); 7.23 (m, 2H, H‐2, 6); 6.71 (m, 2H, H‐3″, 5″); 2.98 (s, 6H, 4″‐NMe2). ^13^C NMR (100 MHz, DMSO‐d_6_): *δ* 187.0 (C═O); 151.9 (C‐4″); 144.7 (C‐9); 141.7 (C‐1); 139.3 (C‐1′); 133.4 (C‐4); 133.2 (C‐4′); 130.6 (C‐2″, 6″); 129.8 (C‐3, 5); 129.4 (C‐3′, 5′); 126.8 (C‐2′, 6′); 122.0 (C‐1″); 118.1 (C‐2, 6); 115.8 (C‐8); 111.7 (C‐3″, 5″); 39.7 (4″‐NMe2).

#### 4.2.6 (*E*)‐*N*‐(4‐(3‐(2,4‐dimethoxyphenyl)acryloyl)phenyl)benzenesulfonamide (12f)

Yellow solid; Yield 77.5% (2.3 g); mp 210–212 °C; Rf 0.53 (*n*‐hexane: ethyl acetate, 3:2). ^1^H NMR (400 MHz, DMSO‐d_6_): *δ* 10.87 (s, 1H, NH); 7.98 (m, 2H, H‐3, 5); 7.92 (d, *J* = 15.6 Hz, 1H, H‐9); 7.84 (m, 2H, H‐2′, 6′); 7.85 (m, 1H, H‐6″); 7.66 (d, *J* = 15.6 Hz, 1H, H‐8); 7.62 (m, 1H, H‐4′); 7.58 (m, 2H, H‐3′, 5′); 7.24 (m, 2H, H‐2, 6); 6.61 (m, 1H, H‐3″); 6.59 (m, 1H, H‐5″); 3.87 (s, 3H, 2″‐OMe); 3.82 (s, 3H, 4″‐OMe). ^13^C NMR (100 MHz, DMSO‐d_6_): *δ* 187.5 (C═O); 163.0 (C‐4″); 159.9 (C‐2″); 141.9 (C‐1); 139.3 (C‐1′); 138.3 (C‐9); 133.2 (C‐4′); 133.1 (C‐4); 130.1 (C‐6″); 129.9 (C‐3, 5); 129.4 (C‐3′, 5′); 126.8 (C‐2′, 6′); 118.8 (C‐8); 118.1 (C‐2, 6); 115.9 (C‐1″); 106.3 (C‐5″); 98.3 (C‐3″); 55.8 (2″‐OMe); 55.5 (4″‐OMe).

#### 4.2.7 (*E*)‐*N*‐(4‐(3‐(3,4‐dimethoxyphenyl)acryloyl)phenyl)benzenesulfonamide (12g)

Yellow solid; Yield 78% (2.31g); mp 138–140  °C; Rf 0.55 (*n*‐hexane: ethyl acetate, 3:2). ^1^H NMR (400 MHz, DMSO‐d_6_): *δ* 10.90 (s, 1H, NH); 8.05 (m, 2H, H‐3, 5); 7.85 (m, 2H, H‐2′, 6′); 7.73 (d, *J* = 15.4 Hz, 1H, H‐8); 7.61 (m, 1H, H‐4′); 7.63 (d, *J* = 15.4 Hz, 1H, H‐9); 7.58 (m, 2H, H‐3′, 5′); 7.49 (d, *J* = 1.5, 1H, H‐2″); 7.33 (dd, *J* = 8.4, 1.5, 1H, H‐6″); 7.27 (m, 2H, H‐2, 6); 6.99 (d, *J* = 8.4 Hz, 1H, H‐5″); 3.84 (s, 3H, 3″‐OMe); 3.79 (s, 3H, 4″‐OMe).^13^C NMR (100 MHz, DMSO‐d_6_): *δ* 187.4 (C═O); 151.2 (C‐4″); 149.0 (C‐3″); 144.0 (C‐9); 142.1 (C‐1); 139.3 (C‐1′); 133.3 (C‐4′); 132.9 (C‐4); 130.1 (C‐3, 5); 129.5 (C‐3′, 5′); 127.5 (C‐1″); 126.7 (C‐2′, 6′); 123.9 (C‐6″); 119.3 (C‐8); 118.1 (C‐2, 6); 111.5 (C‐5″); 110.6 (C‐2″); 55.7 (3″‐OMe); 55.6 (4″‐OMe).

#### 4.2.8 (*E*)‐*N*‐(4‐(3‐(3,4,5‐trimethoxyphenyl)acryloyl)phenyl)benzenesulfonamide (12h)

Yellow solid; Yield 74.2% (2.35 g); mp 210–212 °C; Rf 0.5 (*n*‐hexane: ethyl acetate, 3:2). ^1^H NMR (400 MHz, DMSO‐d_6_): *δ* 10.92 (s, 1H, NH); 8.06 (m, 2H, H‐3, 5); 7.86 (m, 2H, H‐2′, 6′); 7.80 (d, *J* = 15.5 Hz, 1H H‐8); 7.61 (m, 1H, H‐4′); 7.63 (d, *J* = 15.5 Hz, 1H, H‐9); 7.58 (m, 2H, H‐3′, 5′); 7.28 (m, 2H, H‐2, 6); 7.18 (s, 2H H‐2″, 6″); 3.84 (s, 6H, 3″,5″‐OMe); 3.70 (s, 3H, 4″‐OMe). ^13^C NMR (100 MHz, DMSO‐d_6_): *δ* 187.4 (C═O); 153.1 (C‐3″, 5″); 144.0 (C‐9); 142.2 (C‐1); 139.7 (C‐4″); 139.3 (C‐1′); 133.3 (C‐4′); 132.7 (C‐4); 130.2 (C‐1″); 130.2 (C‐3, 5); 129.5 (C‐3′, 5′); 126.7 (C‐2′, 6′); 121.0 (C‐8); 118.0 (C‐2, 6); 106.4 (C‐2″, 6″); 60.1 (4′‐OMe); 56.1 (3″, 5″‐OMe).

### 4.3 Antibacterial Activity

The antibacterial activity of the synthesized compounds **12a–h** was evaluated using the agar disc diffusion method against Gram‐negative bacteria *(E. coli*, and *P. aeruginosa*) and Gram‐positive bacteria (*S. aureus*, and *Staphylococcus*), using established protocols [28]. Müller–Hinton agar plates were prepared under sterile conditions, and freshly grown bacterial cultures were adjusted to a 0.5 McFarland standard (≈1.5 × 10^8 ^CFU/mL) and uniformly swabbed onto the agar surface to obtain confluent growth. Sterile paper discs (6 mm diameter) impregnated with the test compounds at various concentrations (10, 5, 2.5, and 1.25 mg), dissolved in dimethyl sulfoxide (DMSO), were placed on the inoculated plates and incubated at 37 °C for 18–24 h. Sulfamethoxazole was used as the positive control, while DMSO alone served as the negative control. All experiments were performed in triplicate, and antibacterial activity was determined by measuring the diameter of the ZOI (mm), with results expressed as mean ± standard deviation.

### 4.4 MIC Determination

The MICs of compounds **12b** and **12e**, selected on the basis of their notable activity against *E. coli* (Gram‐negative) and *S. aureus* (Gram‐positive), respectively, in the zone of inhibition assay, were determined by the broth macrodilution method following previously reported protocol [55]. Stock solutions of the two compounds were prepared in dimethyl sulfoxide (DMSO) and serially diluted twofold in Mueller–Hinton broth in sterile test tubes to give concentrations of 2, 1, 0.5, 0.25, 0.125, and 0.0625 mg/mL. Bacterial suspensions were adjusted to a 0.5 McFarland standard and further diluted in broth to yield a final inoculum density of approximately 5 × 10^5 ^CFU/mL per tube. Each tube containing 1 mL of the compound dilution was inoculated with 1 mL of the standardized bacterial suspension. A growth control tube (broth and inoculum, no compound) and a sterility control tube (broth and compound, no inoculum) were included for each test series. Tubes were incubated at 35–37 °C for 18–24 h. The MIC was defined as the lowest concentration of test compound that completely inhibited visible bacterial growth, as determined by the absence of turbidity [56]. All determinations were performed in triplicate, and the modal value was reported.

### 4.5 Radical Scavenging Activity Assay

The synthesized derivatives were evaluated for their in vitro radical scavenging activity using the DPPH assay, with ascorbic acid as a standard [28]. This assay measures the ability of the compounds to donate hydrogen atoms or electrons by monitoring the reduction of the purple‐colored DPPH radical to its yellow nonradical form. To prepare for the assay, five serial dilutions were made from a 2 mg/mL stock solution to achieve concentrations of 500, 250, 125, 62.5, and 31.25 μg/mL. For the DPPH assay, 1 mL of each diluted sample was combined with 1 mL of a methanolic DPPH solution (4 mg/100 mL). The reaction mixtures were incubated in the dark at room temperature for 30 min. Following incubation, the absorbance was measured at 517 nm using a UV–vis spectrophotometer. The percentage of inhibition of the DPPH radical was calculated using the following formula:

$$\text{\%}\mathrm{Scavenging}=(A_{\mathrm{control}}-A_{\mathrm{sample}})/A_{\mathrm{control}}\times 100$$

where *A*_control_ is the absorbance of the DPPH solution without the test compound, and *A*_sample_ is the absorbance of the test compound with DPPH. All experiments were performed in triplicate.

### 4.6 In Vitro Anti‐Inflammatory Activity

#### 4.6.1 Albumin Denaturation Inhibition Activity

The anti‐inflammatory activities of the synthesized compounds were assayed by the protein denaturation inhibition assay as described earlier, with slight modifications [57]. A reaction mixture containing 0.2 mL of bovine serum albumin (BSA) (1% aqueous solution), 2.8 mL of Tris‐buffered saline (TBS, pH 6.4), and 2.0 mL of the test compounds at various concentrations was prepared to obtain final concentrations of 50, 100, 200, 400, and 800 µg/mL. The mixtures were incubated at 37 °C for 15 min, followed by heating at 70 °C for 5 min. After cooling to room temperature, the absorbance was measured at 660 nm. An equal volume of distilled water served as the blank. Diclofenac sodium at corresponding concentrations was used as the reference standard for comparison. The experiment was performed in triplicate. The percentage inhibition of protein denaturation was calculated as follows:

$$\text{\%}\mathrm{Inhibition}=(A_{\mathrm{control}}–A_{\mathrm{test}}\text{)}\text{/}A_{\mathrm{control}}\times 100$$

where *A*_test_  =  absorbance of the test sample, *A*_control _ =  absorbance of the control.

#### 4.6.2 Inhibition of Proteinase Action

The trypsin inhibitory activity was evaluated according to the method reported before with some modifications [58]. The reaction mixture (2.0 mL) consisted of 1.0 mL of Tris‐HCl buffer (20 mM, pH 7.4), trypsin (60 µg), and 1.0 mL of the test sample at different concentrations (50, 100, 200, 400, and 800 µg/mL) or diclofenac sodium as the reference standard. After pre‐incubation at room temperature for 5 min, 1.0 mL of 0.8% (w/v) casein was added to initiate the reaction. The mixture was further incubated for 20 min at room temperature. The reaction was terminated by adding 2.0 mL of 70% perchloric acid, resulting in turbidity. The samples were centrifuged at 3000 rpm for 5 min, and the absorbance of the supernatant was measured at 210 nm against a blank containing buffer only. All experiments were performed in triplicate. The percentage inhibition of proteinase activity was calculated using the following equation:

$$\mathrm{Percentage}\;\mathrm{inhibition}\;\text{(}\text{\%}\text{)}\;=\;(A_{\mathrm{control}}-A_{\mathrm{sample}}\text{)}\text{/}A\times \;100$$

where, *A*_control_ represents the absorbance of the control, and *A*_sample_ represents the absorbance of the test sample.

### 4.7 Molecular Docking Studies

The synthesized compounds were subjected to in silico molecular docking studies against selected biological targets using established protocols [59]. Ligand structures were initially sketched using ChemDraw 16.0 (PerkinElmer Informatics, Waltham, MA, USA) and subsequently energy‐minimized in ChemBio3D to obtain stable conformations. The optimized ligands were then converted into .pdbqt format using MGLTools/AutoDockTools 1.5.7 (The Scripps Research Institute, La Jolla, CA, USA) [60]. The model proteins used in this study included TDHS PDB ID: 1AJ0 (2.00 Å resolution) [61], 1AD4 (2.40 Å resolution) [62], human myeloperoxidase (PDB ID: 1DNU, 1.85 Å resolution) [63], Cyclooxygenase‐2 (PDB ID: 5IKR, 2.34 Å resolution) [64] and Topoisomerase *IIα* (PDB ID: 4FM9, 2.90 Å resolution) [65], all retrieved from the Protein Data Bank (https://www.rcsb.org/) [66].

Prior to docking, protein structures were prepared by removing co‐crystallized ligands, and water molecules and adding polar hydrogens using Discovery Studio Visualizer, and the specific binding sites were identified [67]. Molecular docking was performed using AutoDock Vina 1.2.0 (Center for Computational Structural Biology, The Scripps Research Institute, La Jolla, CA, USA) [68]. A blind docking approach was employed by defining a grid box encompassing the entire protein structure to allow unbiased exploration of potential binding sites. For each ligand, nine binding poses were generated, and the most favorable binding poses were selected based on a combination of the highest binding affinity scores and key stabilizing interactions, including hydrogen bonding and hydrophobic contacts with functionally important residues. Notably, the top‐ranked poses consistently localized within known or biologically relevant binding pockets reported in structural and literature data. Ligand–target binding interactions were visualized in 2D and 3D using Discovery Studio Visualizer (Dassault Systèmes BIOVIA, San Diego, CA, USA).

The docking protocol was validated using a self‐docking (re‐docking) approach for protein targets with available co‐crystallized ligands. The native ligands were extracted from their respective crystal structures and re‐docked into the corresponding binding sites using the same grid parameters and docking settings described above. The accuracy of the protocol was evaluated by calculating the root mean square deviation (RMSD) between the experimental and predicted ligand poses, with values ≤2.0 Å considered acceptable for reliable docking performance [34].

For protein targets lacking co‐crystallized ligands, RMSD‐based validation was not applicable; however, docking was performed using the same validated parameters to ensure methodological consistency across all systems.

### 4.8 In Silico Pharmacokinetics (Drug likeness) and Toxicity Studies

An in silico evaluation was conducted to assess the drug‐likeness and toxicity profiles of the synthesized compounds in relation to their suitability as orally active drugs, based on Lipinski’s Rule of Five [50]. Drug‐likeness and key pharmacokinetic parameters were predicted using the SwissADME web tools (https://www.swissadme.ch), providing information on relevant molecular descriptors [51]. Potential organ‐specific toxicities and other toxicological endpoints were further evaluated using SwissADME, ProTox‐3 (https://tox.charite.de/protox3/), and Property Explorer. The predicted pharmacokinetic and toxicity profiles of the synthesized compounds were compared with those of the clinically used reference drug, sulfamethoxazole [69].

## Author Contributions

Conceptualization: Yadessa Melaku and Endale Mulugeta. Methodology and software‐related tasks: Mlis Belete. Writing–original draft preparation: Mlis Belete. Writing, reviewing, and editing, as well as supervision, formal analysis, data curation: Yadessa Melaku and Endale Mulugeta. NMR analysis and reviewing: Daniel Rentsch. Antibacterial, antioxidant and anti‐inflammatory activity evaluations conducted: Muhdin Aliye and Kebede Shenkutie. All authors have read and agreed to the published version of the manuscript.

## Funding

The authors have nothing to report.

## Conflicts of Interest

The authors declare no conflicts of interest.

## Supporting information

Supplementary Material

## Data Availability

The data that support the finding of this study are available in the Supporting Information of this article.
