# Supplementary material for: Synthesis of Chalcone‐Sulfonamide Hybrids as Antibacterial, Antioxidant, and Anti‐Inflammatory Agents With Comprehensive In Silico and ADMET Profiling
Source: ChemistryOpen. 2026 Aug 27;15(9):e70292. doi: 10.1002/open.70292 (PMC13519690; doi:10.1002/open.70292)
Supplement: Supplementary file 1 [file OPEN-15-e70292-s001.pdf]

Supporting Information

Synthesis of Chalcone-Sulfonamide Hybrids as Antibacterial, Antioxidant, and Anti-inflammatory Agents with Comprehensive *In-Silico* and ADME Profiling

Mlis Belete *[a], Endale Mulugeta [a,c], Daniel Rentsch [b], Muhdin Aliye [a], Kebede Shenkutie [a], and Yadessa Melaku *[a]

[a] Department of Applied Chemistry, College of Applied Natural Science, Adama Science and Technology University, P.O. Box 1888, Adama, Ethiopia

[b] Laboratory for Functional Polymers, Empa, Swiss Federal Laboratories for Materials Science and Technology, Überlandstrasse 129, 8600 Dübendorf, Switzerland

[c] Department of Chemistry, College of Natural Sciences, Kyungpook National University, Taegu 41566, South Korea

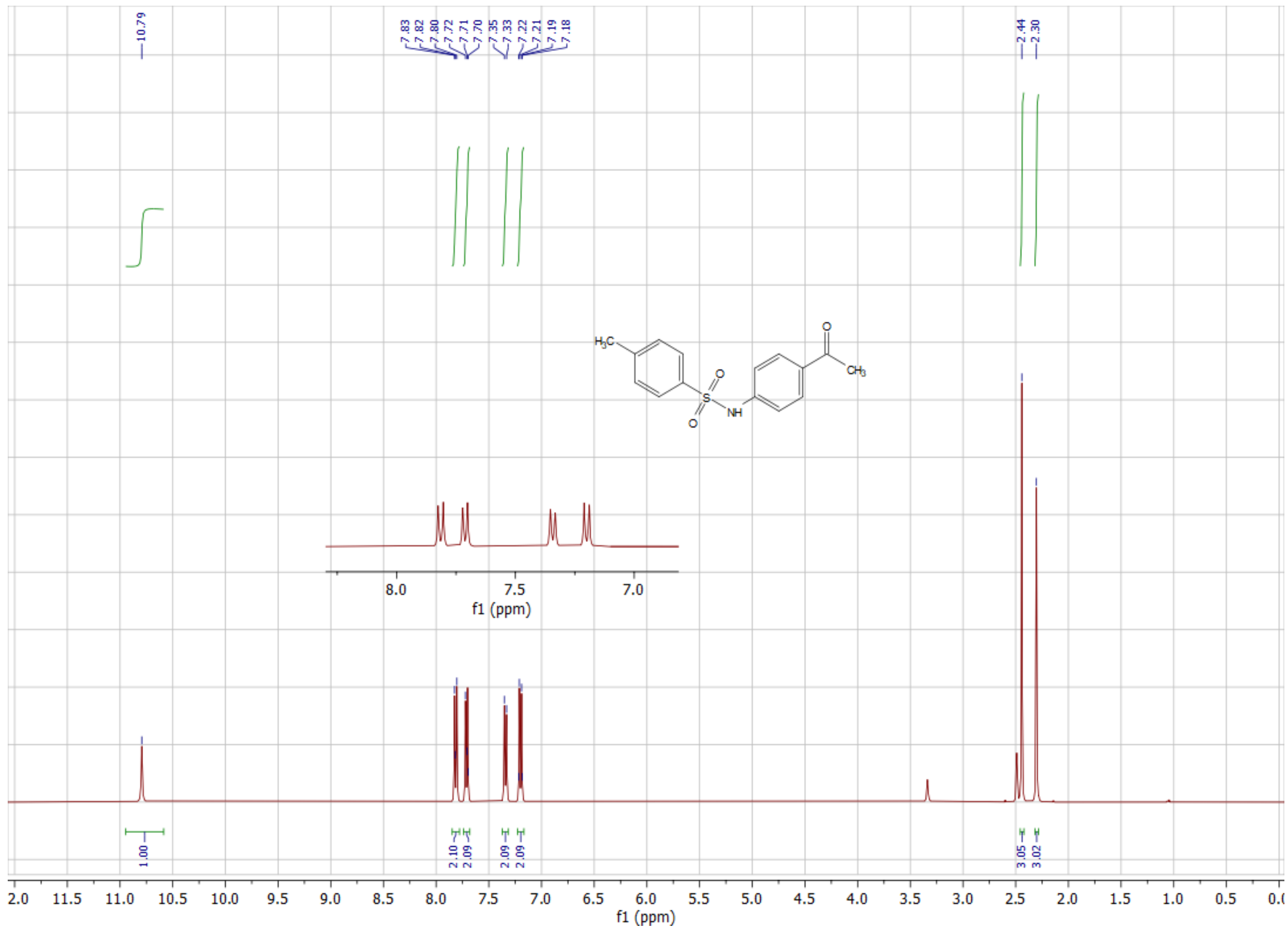

Fig. S1: ^{1}H-NMR spectrum (400 MHz, DMSO-d_6) of compound **10a**

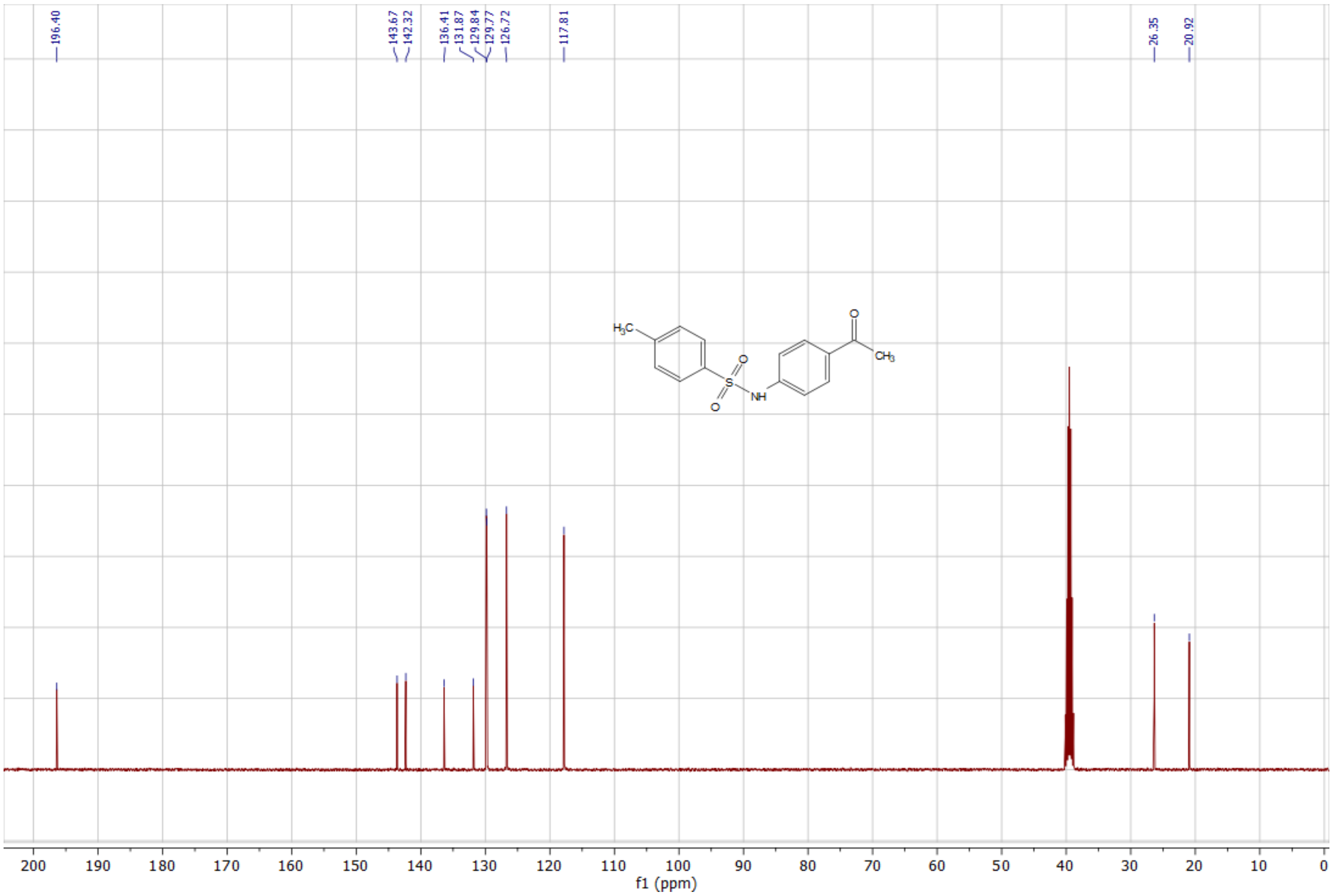

Fig. S2: ^{13}C-NMR spectrum (100 MHz, DMSO-d_6) of compound **10a**

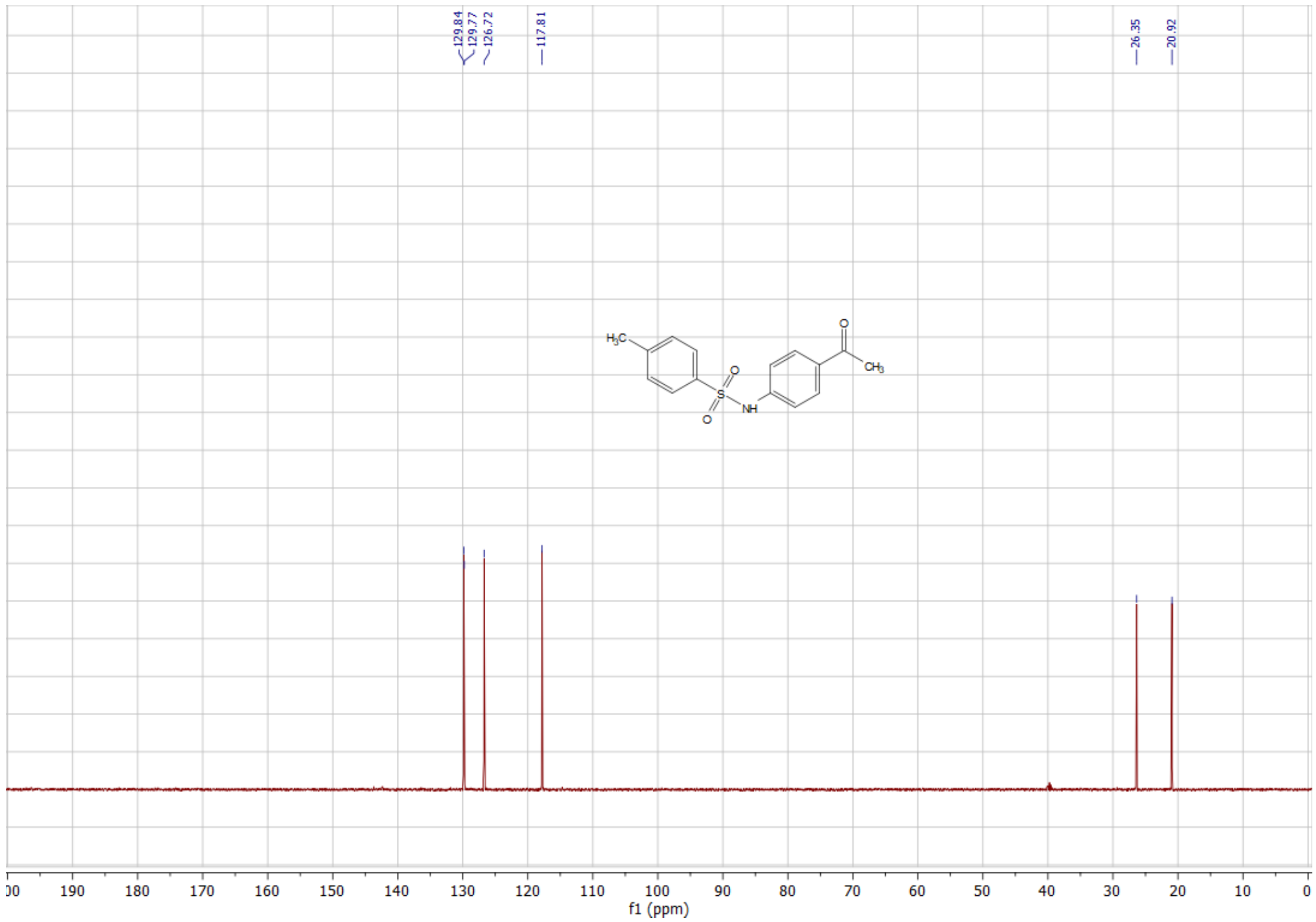

Fig. S3: DEPT NMR spectrum (100 MHz, DMSO-d_6) of compound **10a**

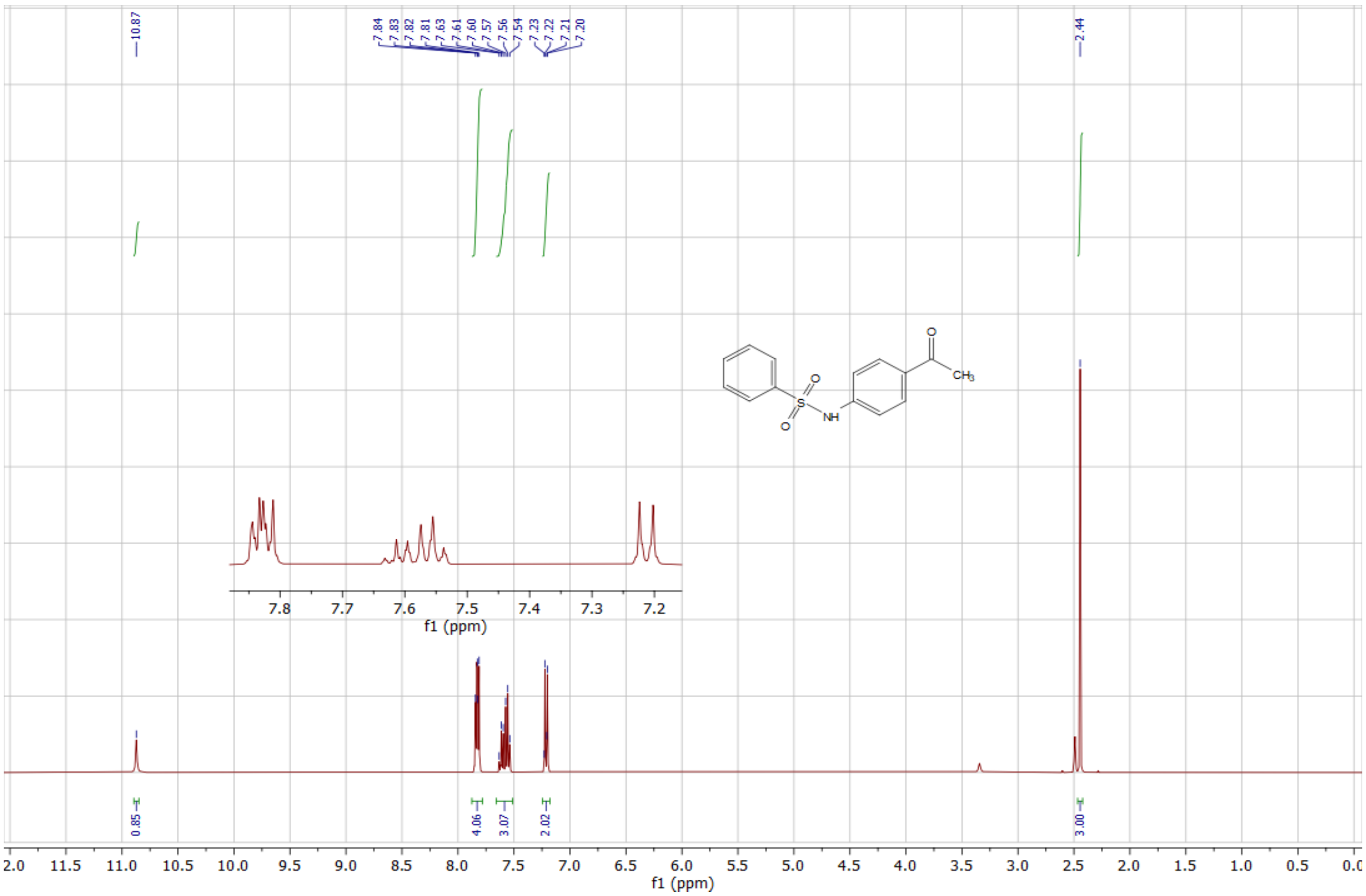

Fig. S4: ^{1}H-NMR spectrum (400 MHz, DMSO-d_6) of compound **10b**

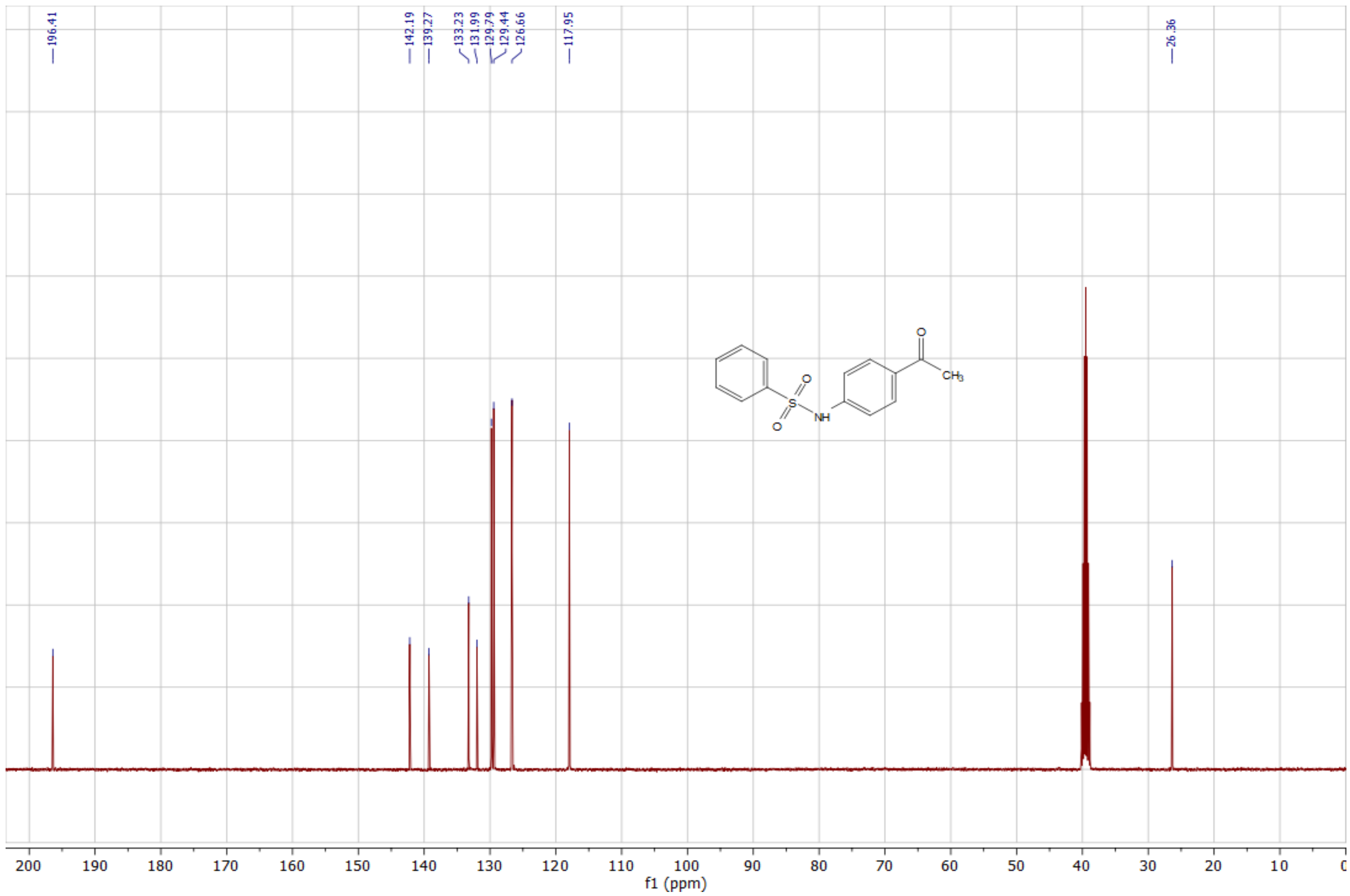

Fig. S5: ^{13}C-NMR spectrum (100 MHz, DMSO-d_6) of compound **10b**

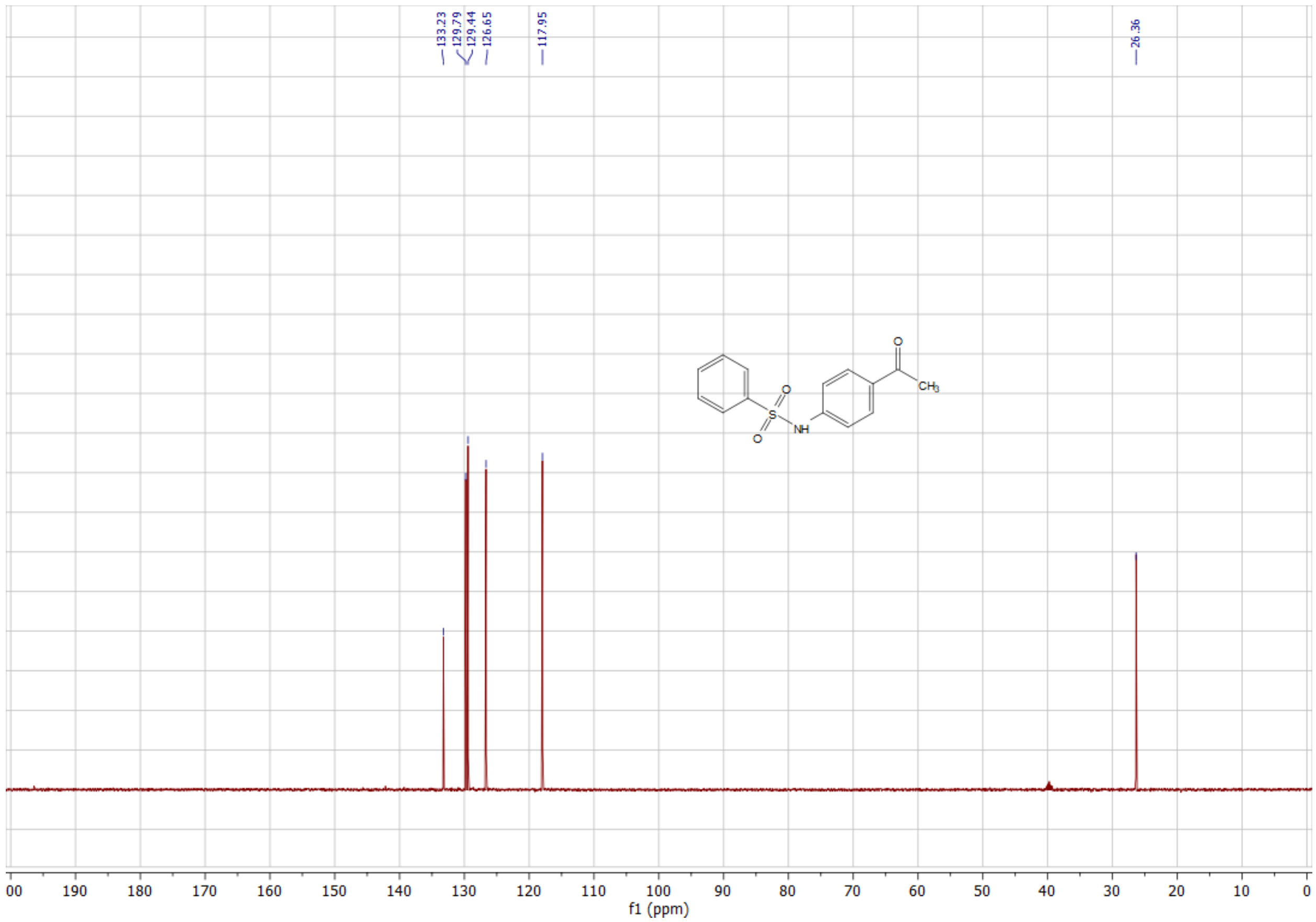

Fig. S6: DEPT NMR spectrum (100 MHz, DMSO-d_6) of compound **10b**

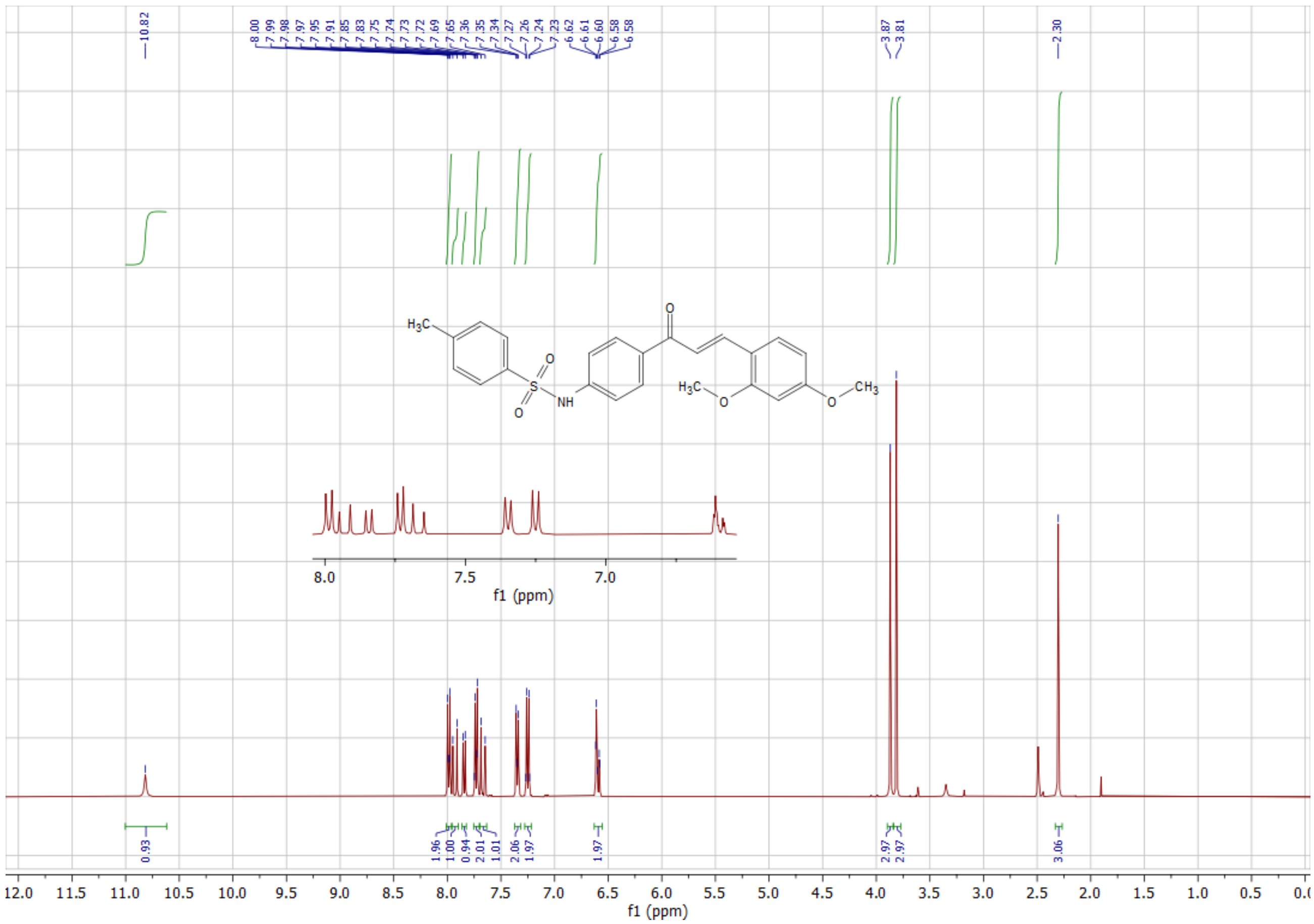

Fig. S7: ^{1}H-NMR spectrum (400 MHz, DMSO-d_6) of compound **12a**

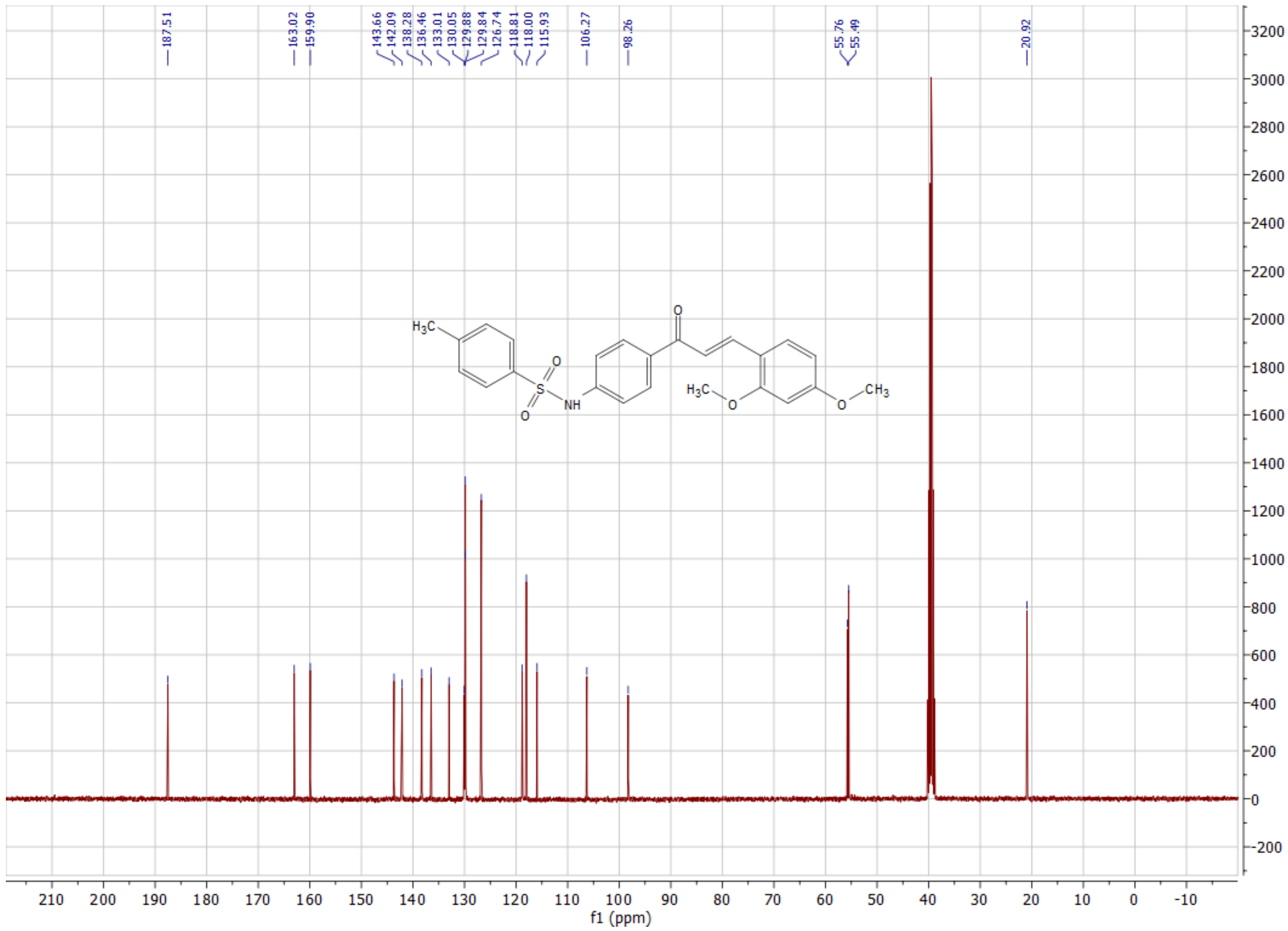

Fig. S8: ^{13}C-NMR spectrum (100 MHz, DMSO-d$_6$) of compound **12a**

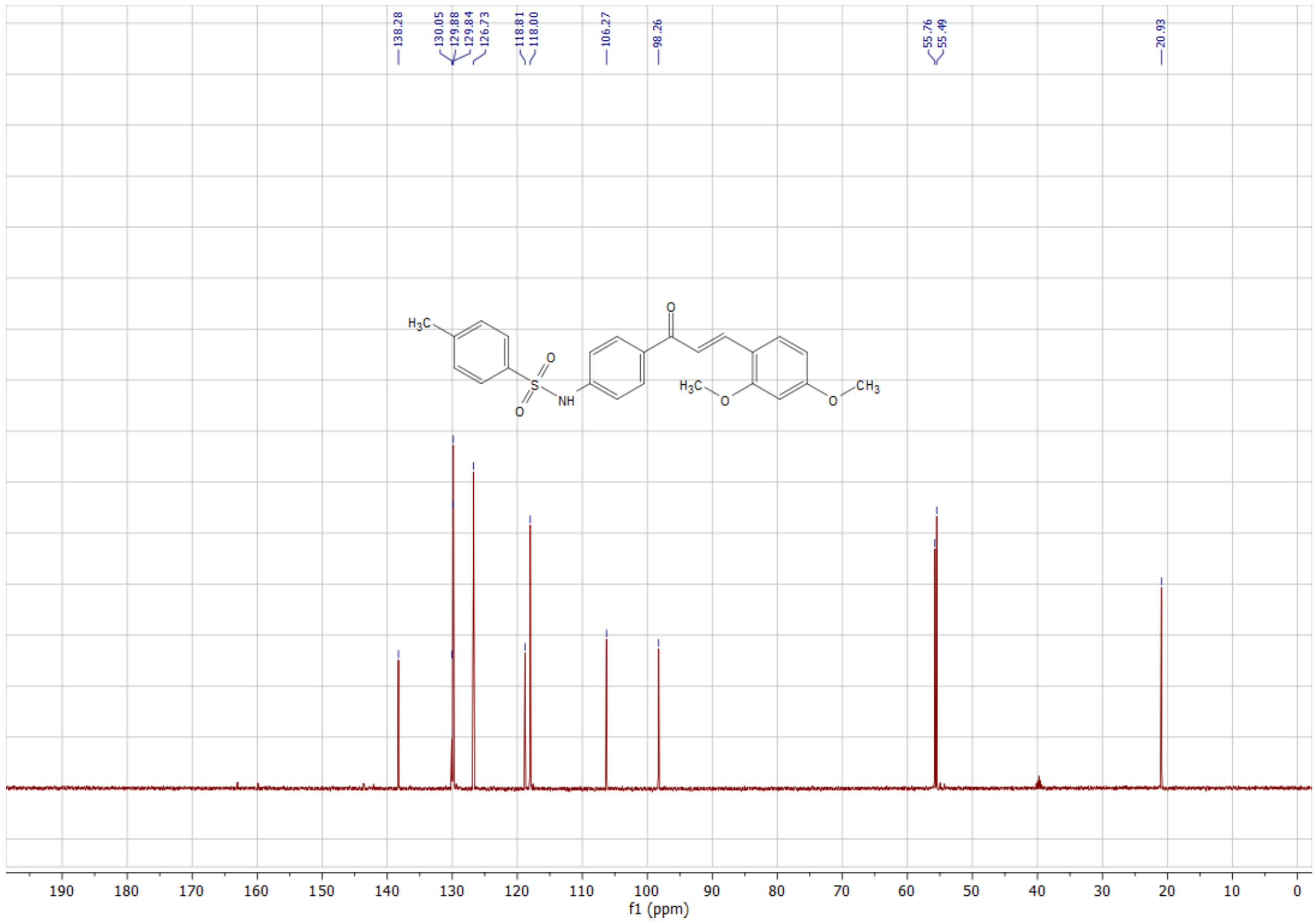

Fig. S9: DEPT NMR spectrum (100 MHz, DMSO-d_6) of compound **12a**

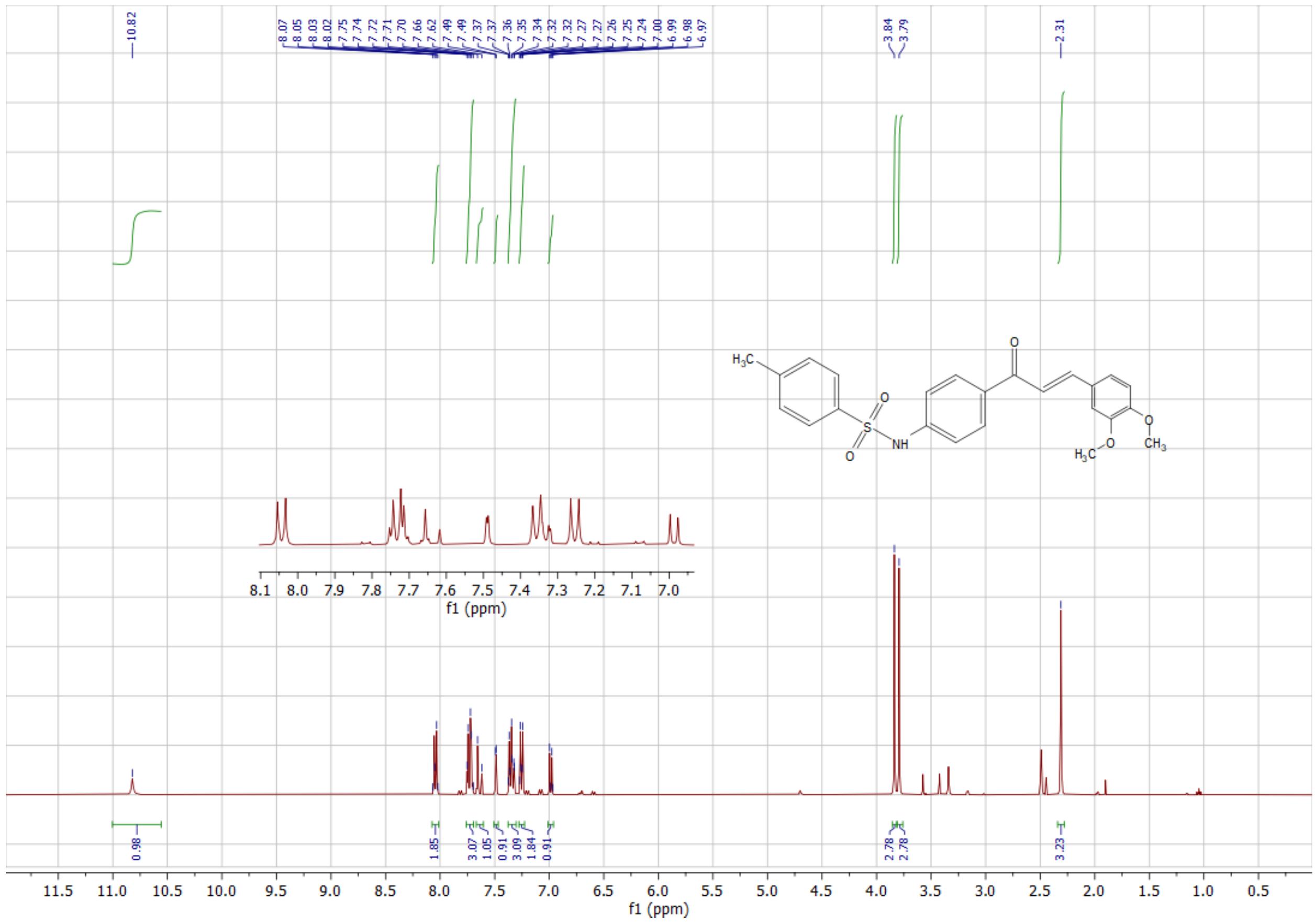

Fig. S10: ^{1}H-NMR spectrum (400 MHz, $DMSO_{d\text{-}6}$) of compound **12b**

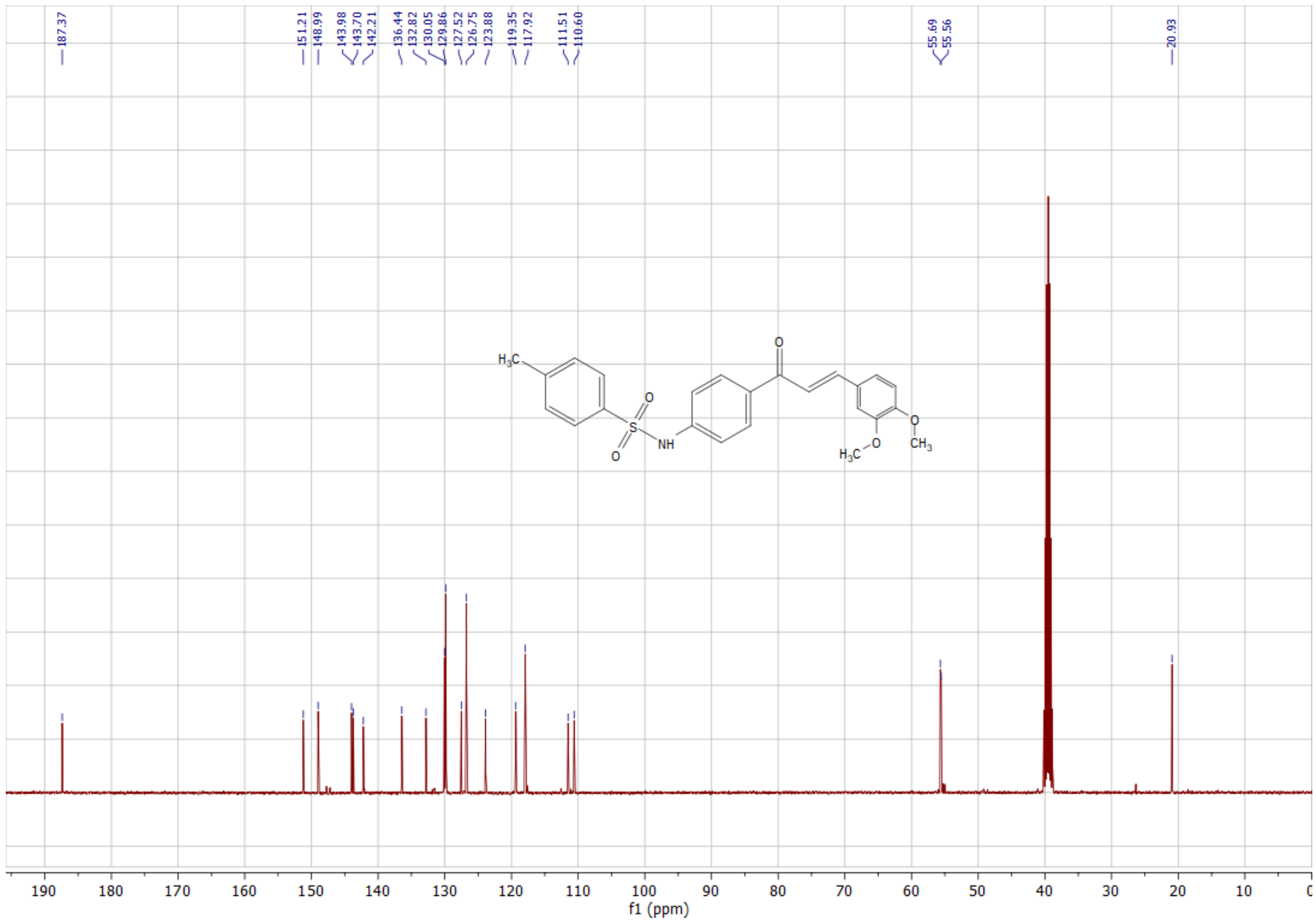

Fig. S11: ^{13}C-NMR spectrum (100 MHz, $DMSO_{d6}$) of compound **12b**

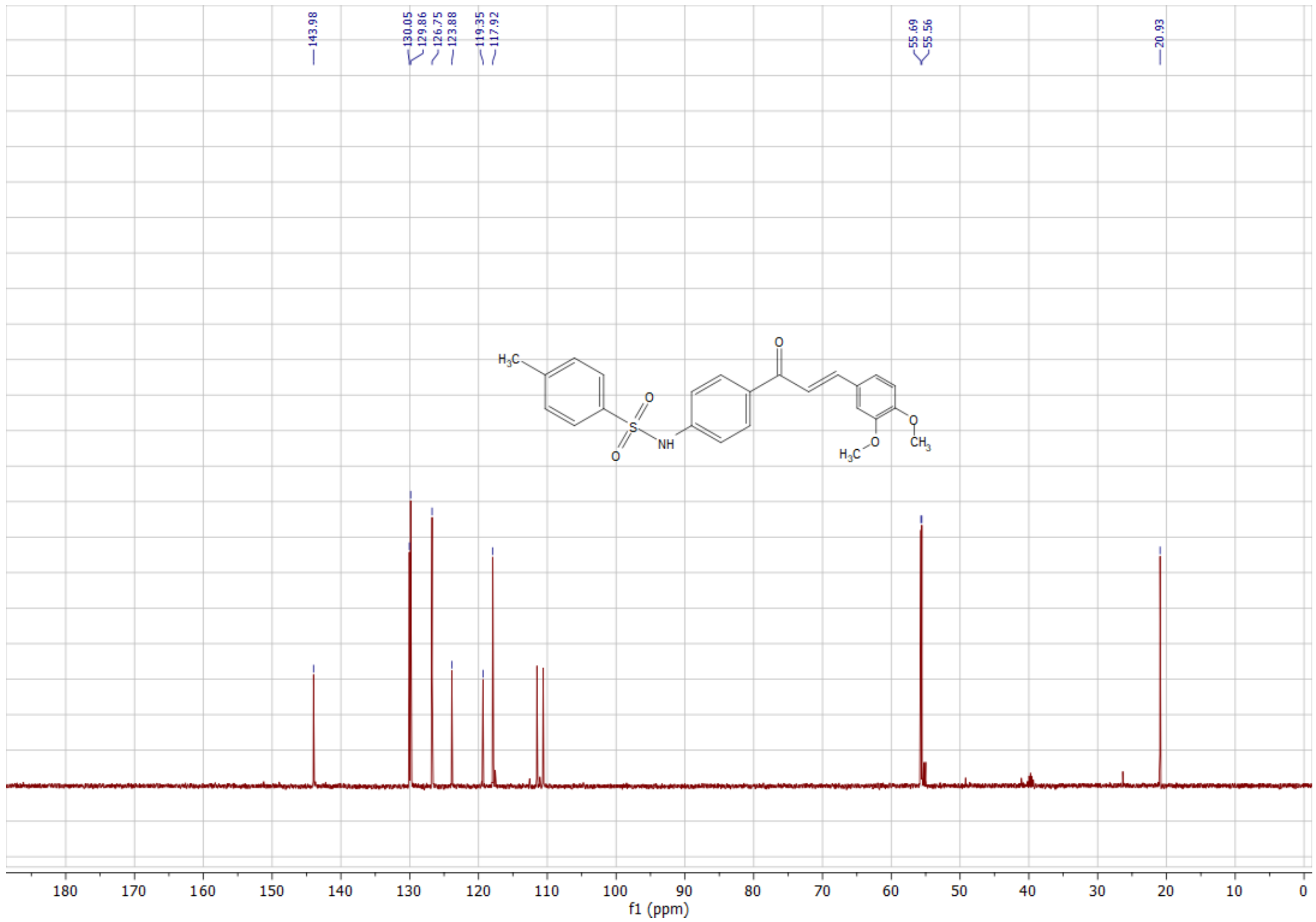

Fig. S12: DEPT NMR spectrum (100 MHz, $DMSO_{d6}$) of compound **12b**

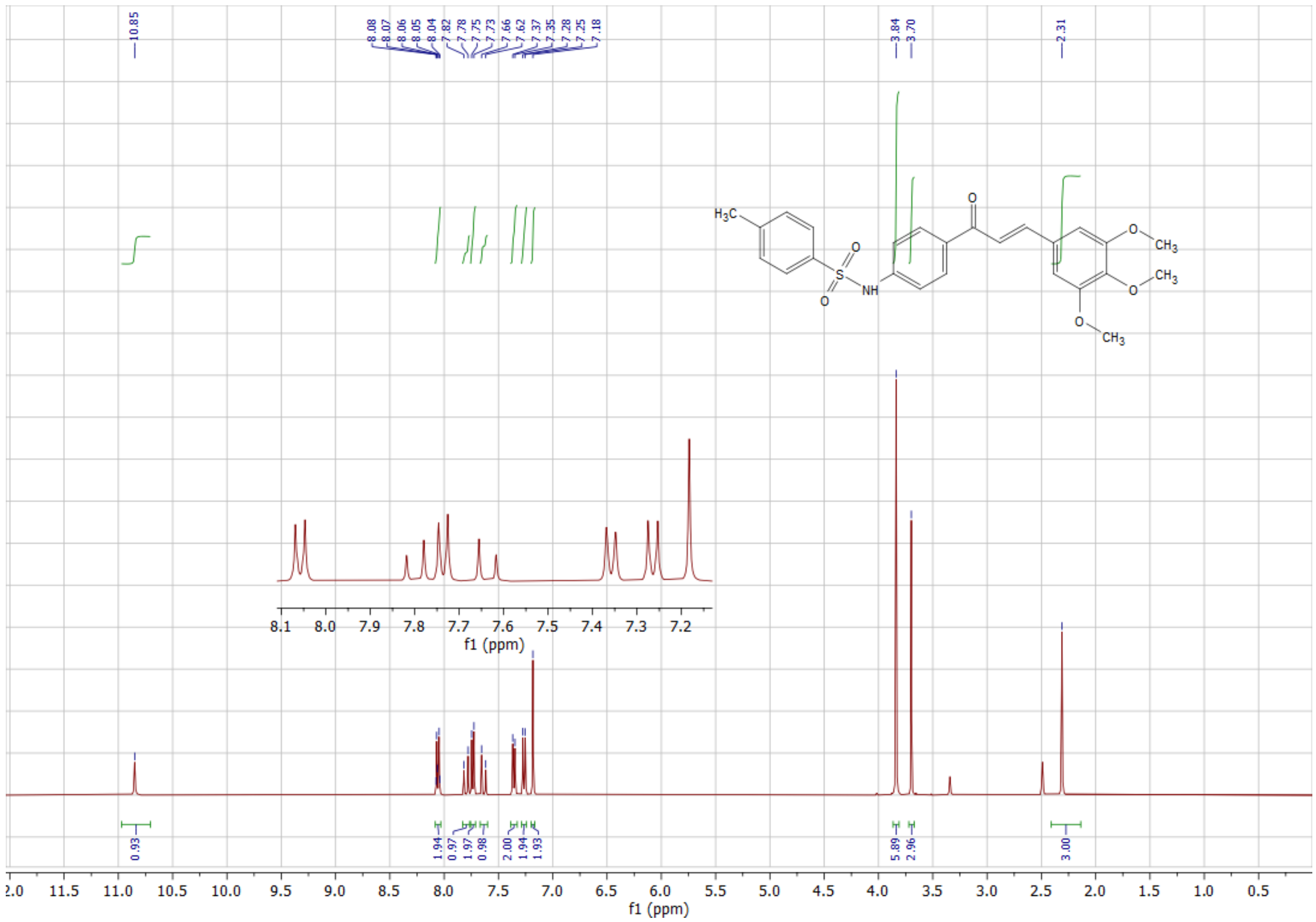

Fig. S13: ^{1}H-NMR spectrum (400 MHz, $DMSO_{d6}$) of compound **12c**

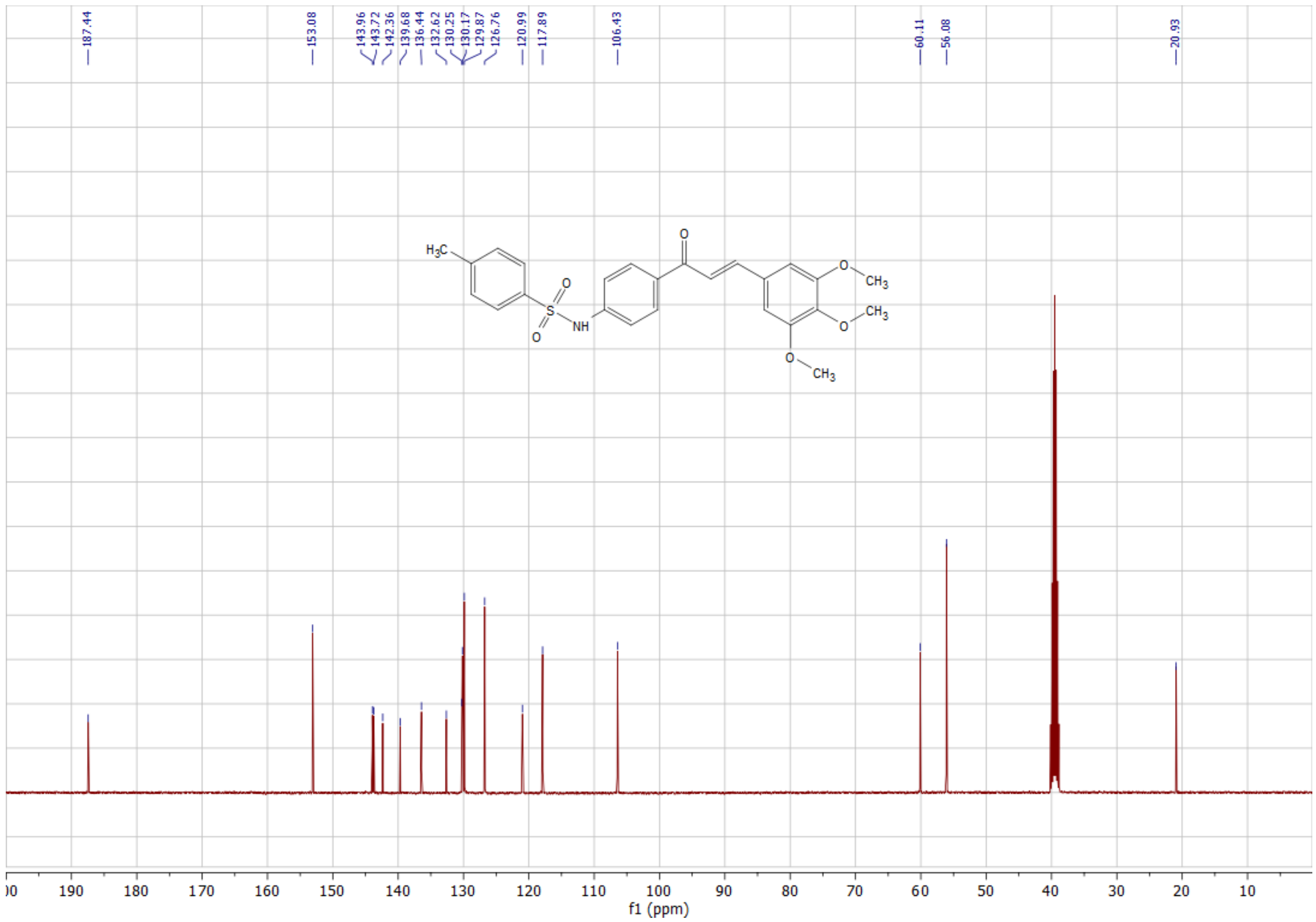

Fig. S14: ^{13}C-NMR spectrum (100 MHz, $DMSO_{d6}$) of compound **12c**

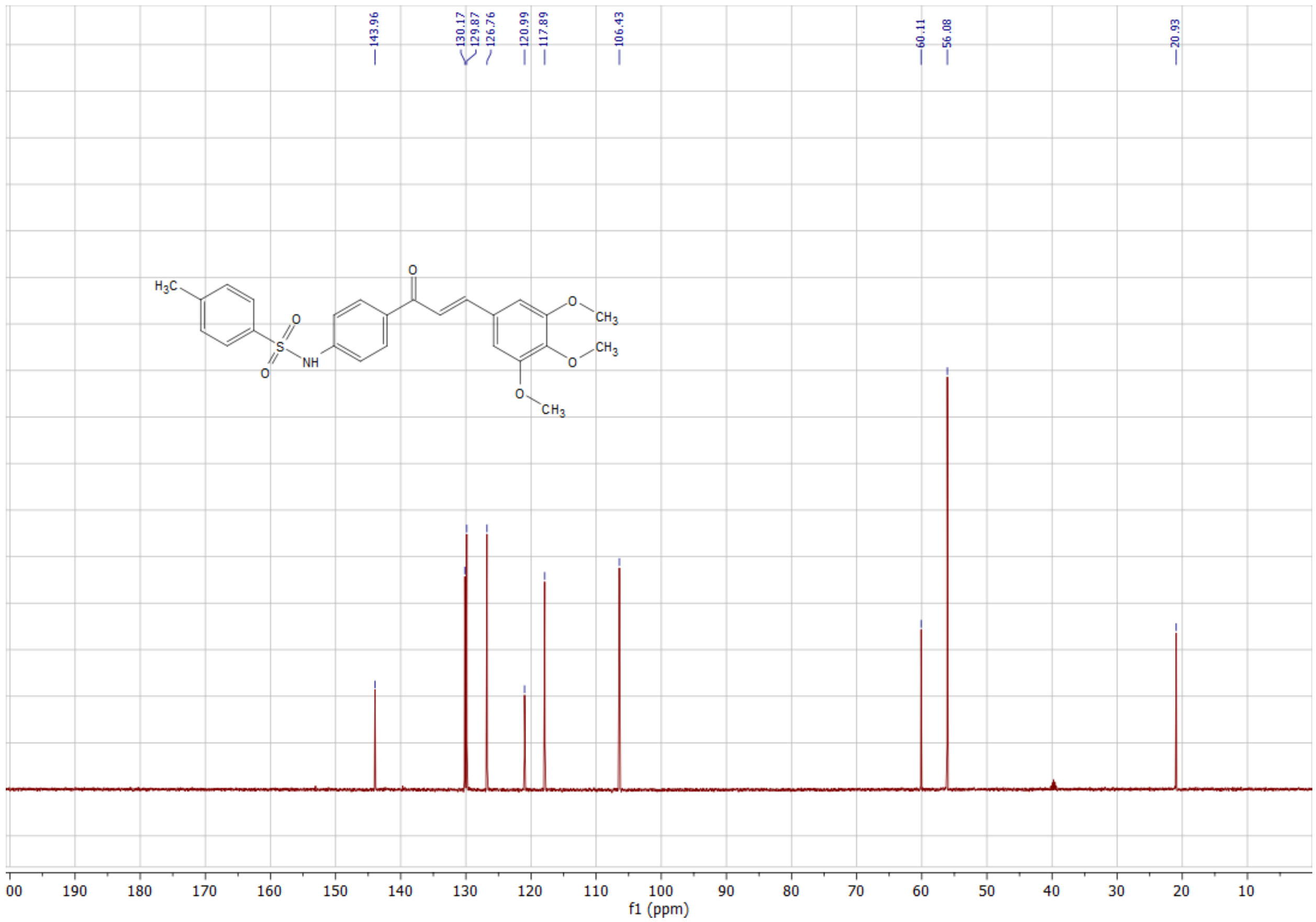

Fig. S15: DEPT NMR spectrum (100 MHz, $DMSO_{d6}$) of compound **12c**

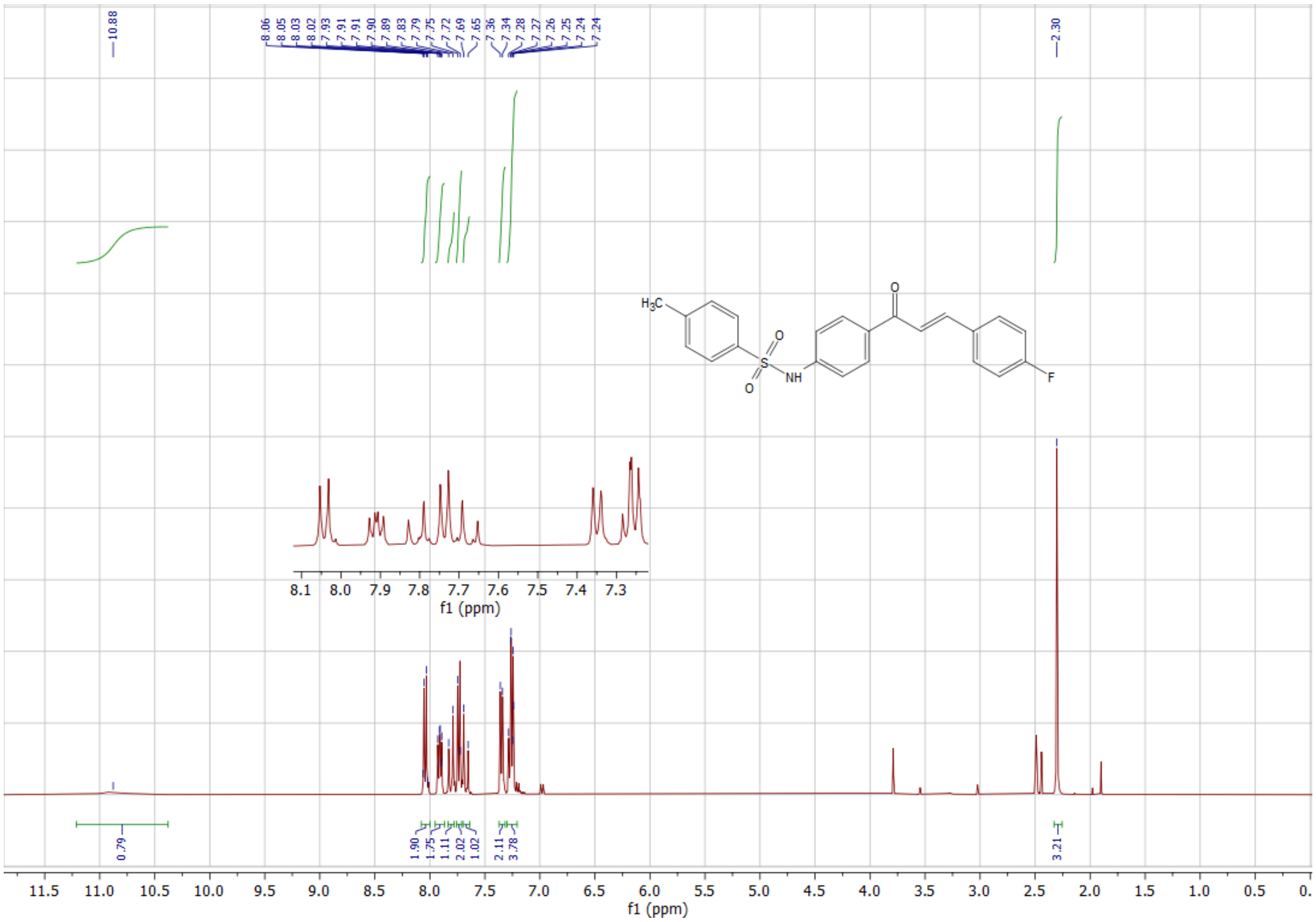

Fig. S16: ^{1}H-NMR spectrum (400 MHz, $DMSO_{d6}$) of compound **12d**

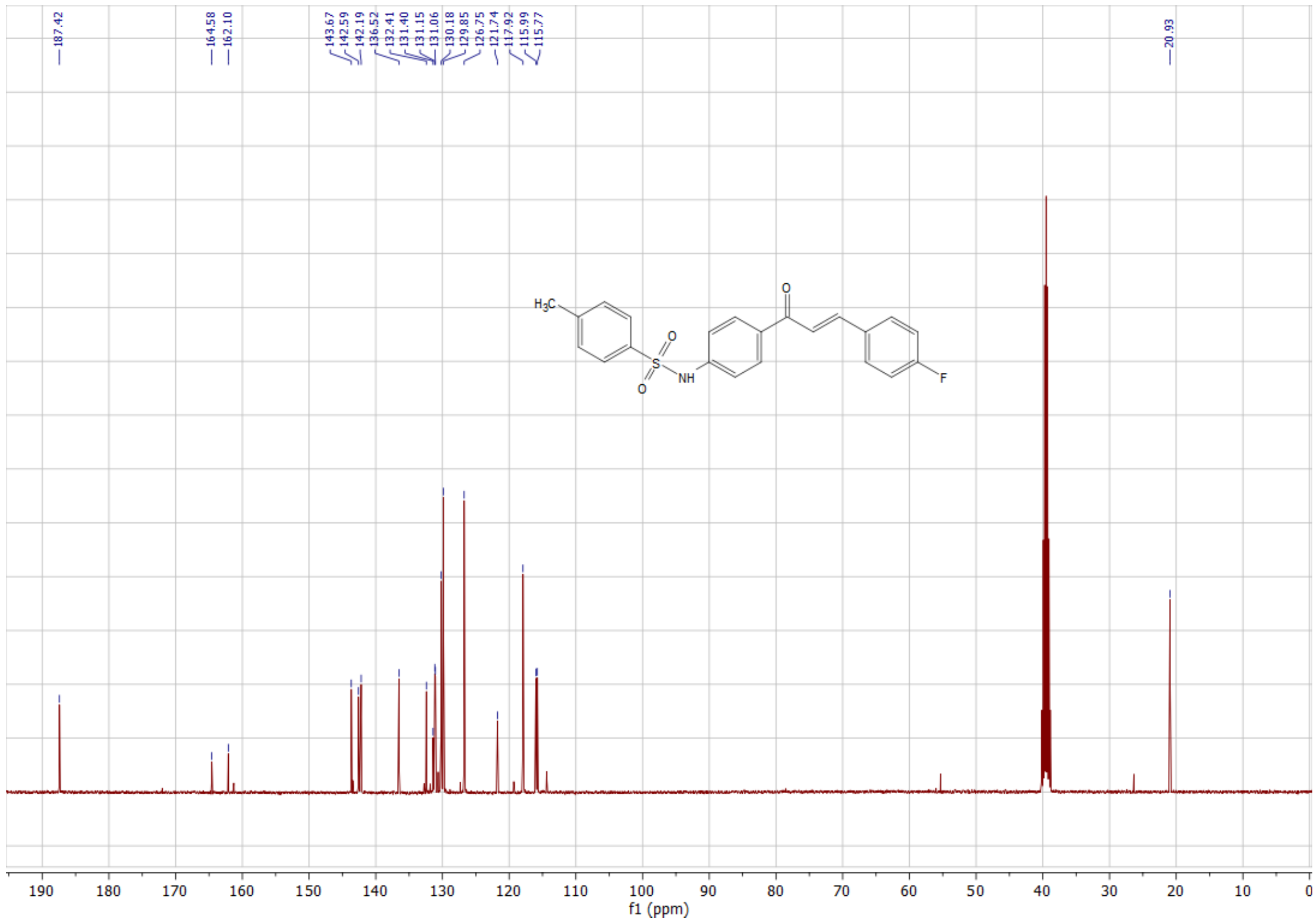

Fig. S17: ^{13}C-NMR spectrum (100 MHz, DMSO-d_6) of compound **12d**

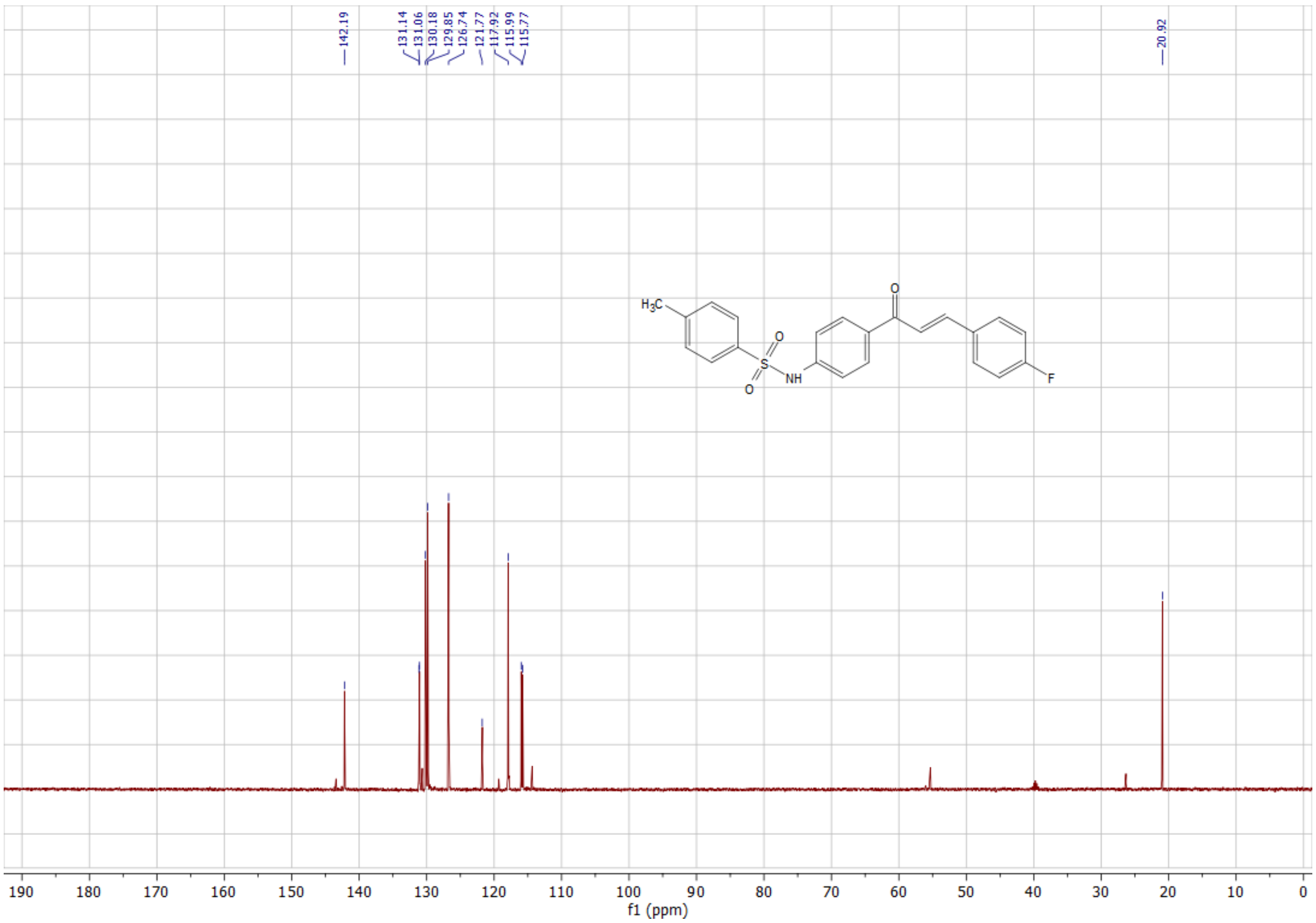

Fig. S18: DEPT NMR spectrum (100 MHz, DMSO-d_6) of compound **12d**

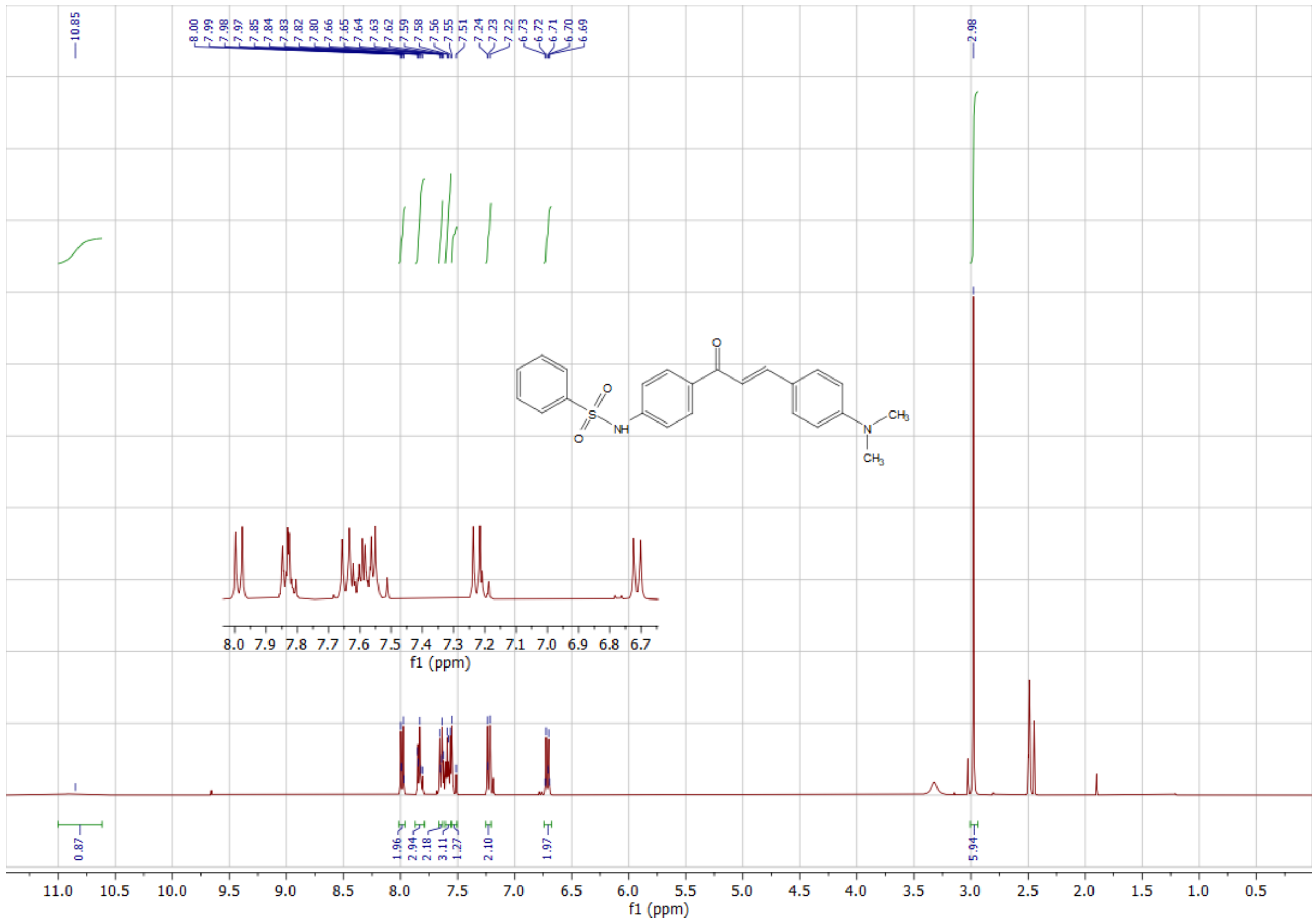

Fig. S19: ^{1}H-NMR spectrum (400 MHz, DMSO-d_6) of compound **12e**

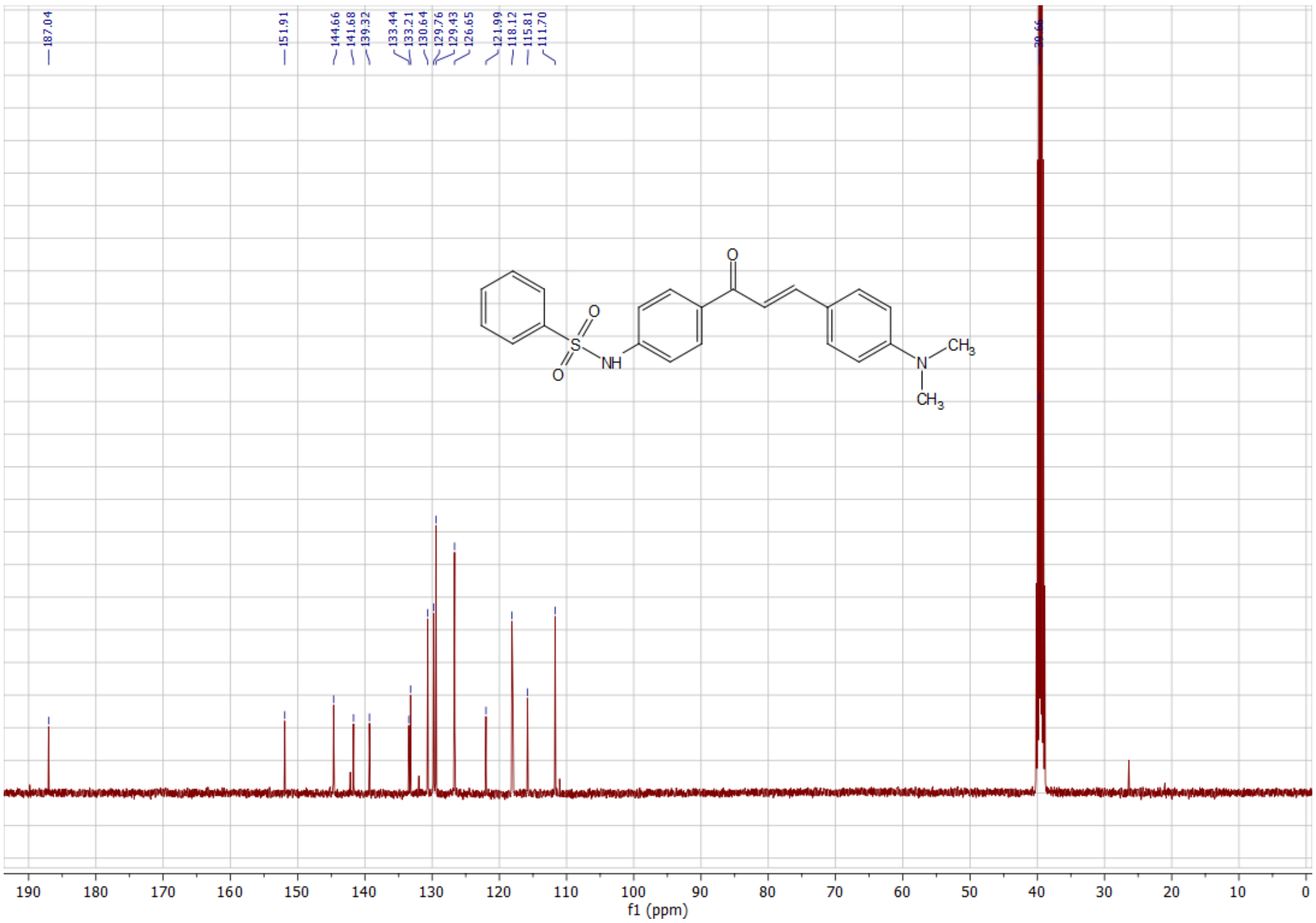

Fig. S20: ^{13}C-NMR spectrum (100 MHz, DMSO-d_6) of compound **12e**

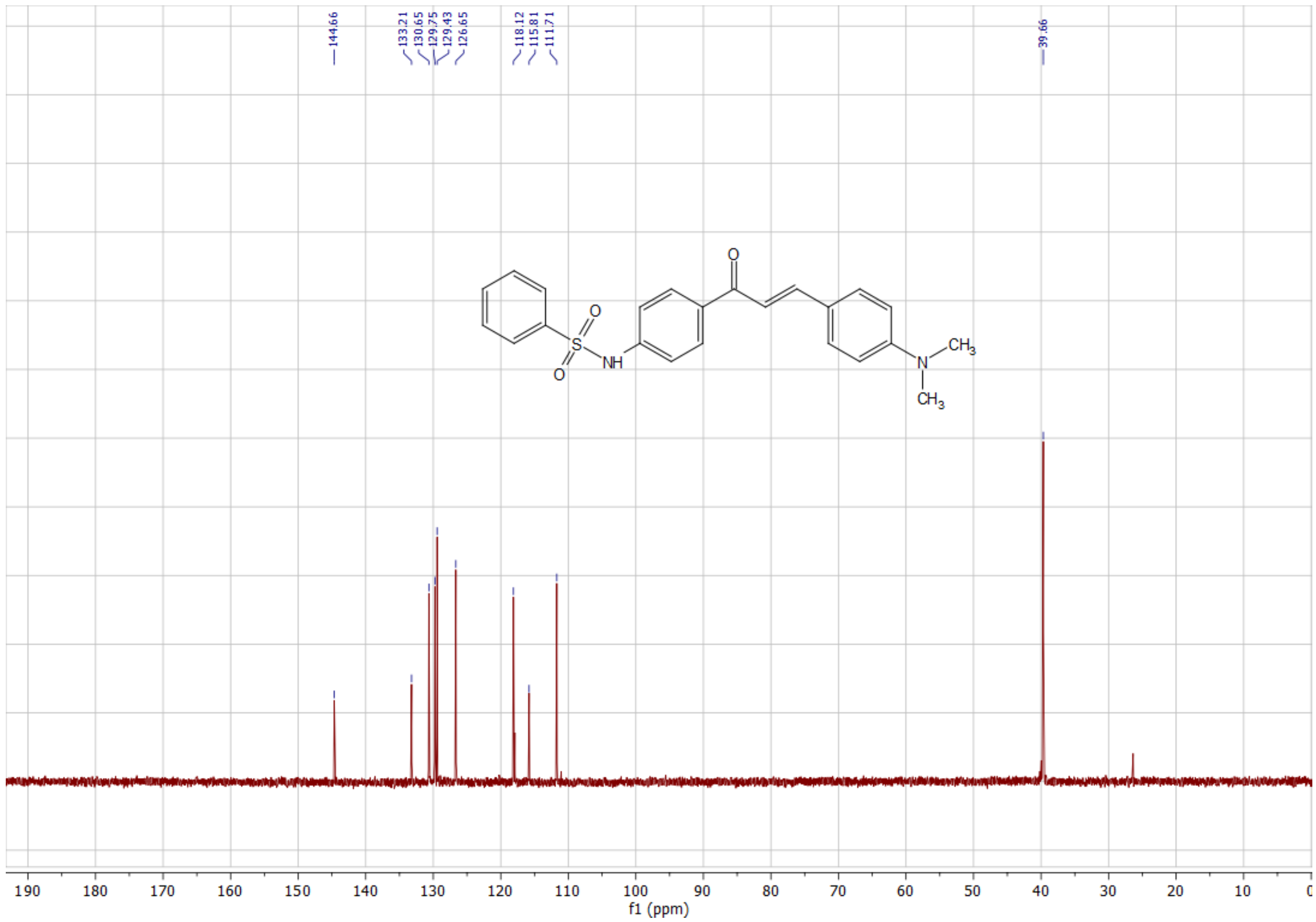

Fig. S21: DEPT NMR spectrum (100 MHz, DMSO-d_6) of compound **12e**

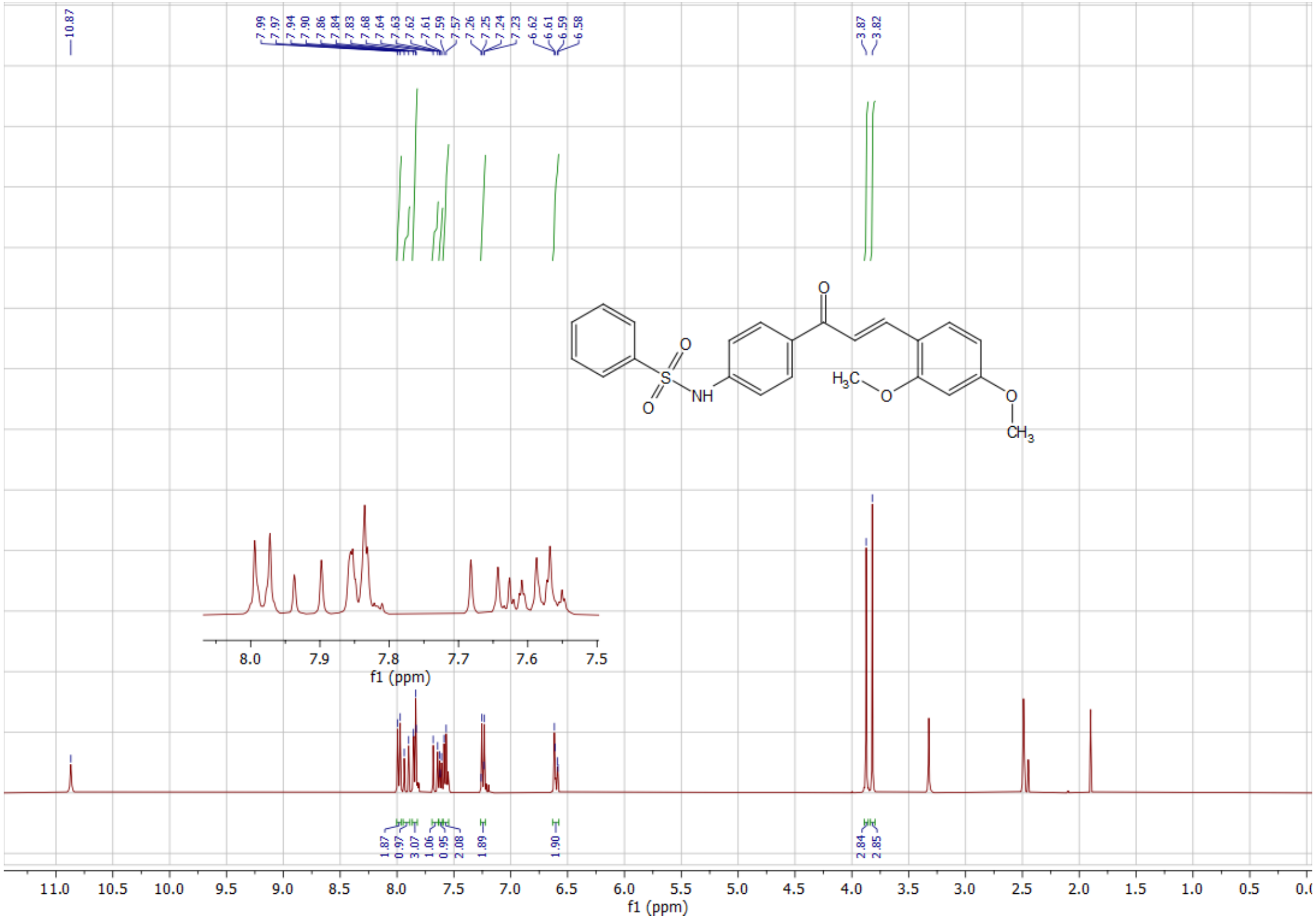

Fig. S22: ^{1}H-NMR spectrum (400 MHz, DMSO-d_6) of compound **12f**

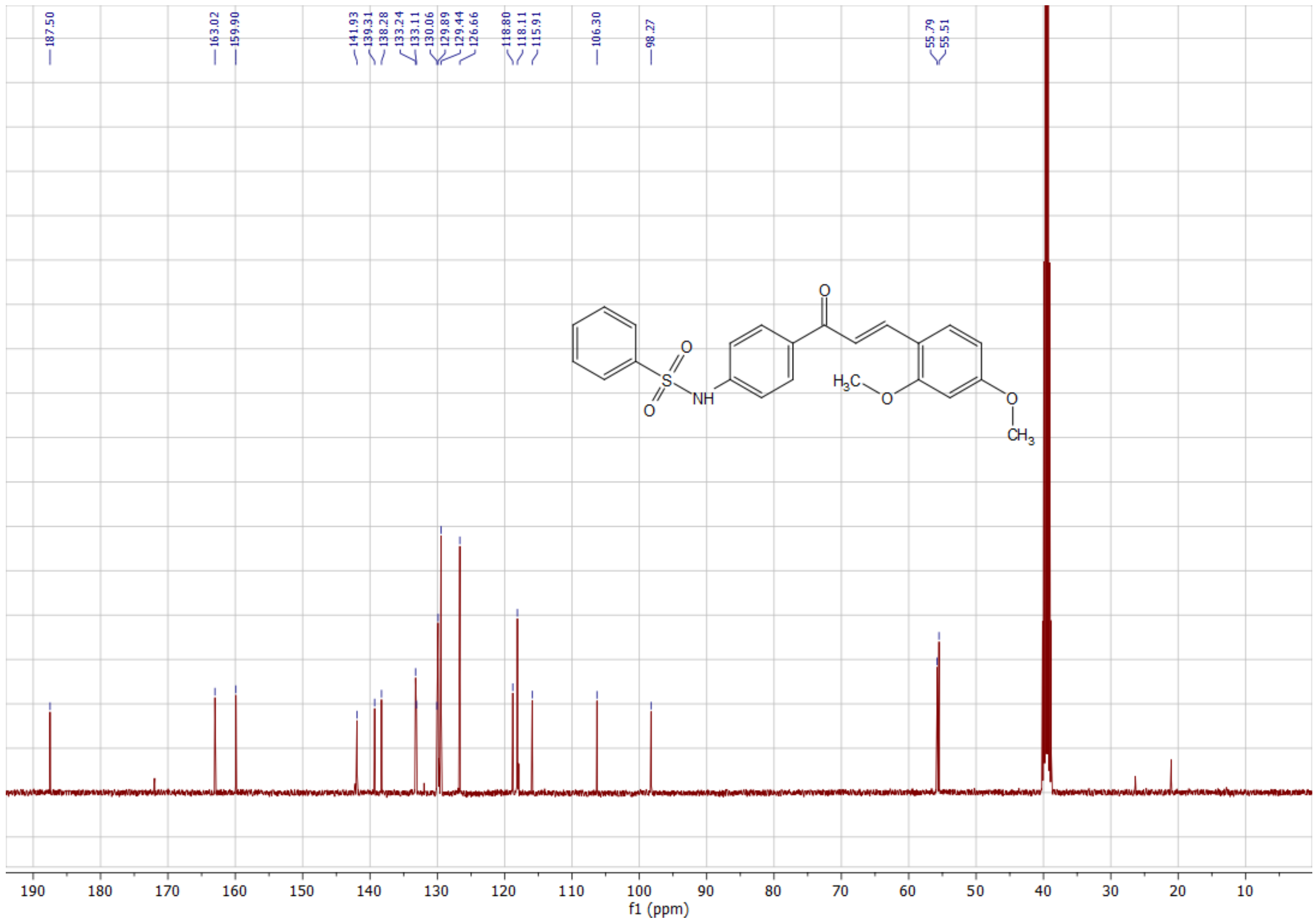

Fig. S23: ^{13}C-NMR spectrum (100 MHz, DMSO-d_6) of compound **12f**

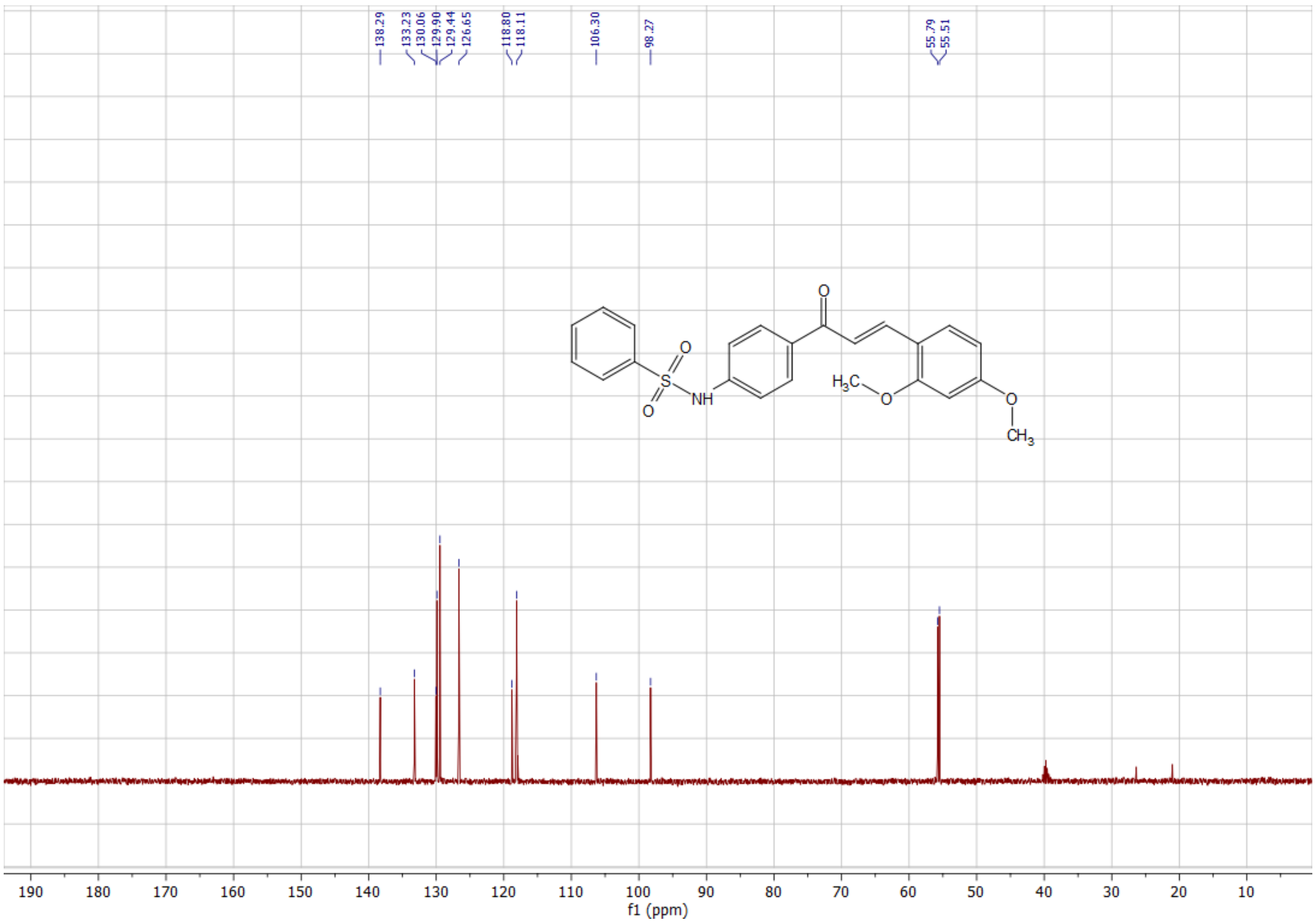

Fig. S24: DEPT NMR spectrum (100 MHz, DMSO-d_6) of compound **12f**

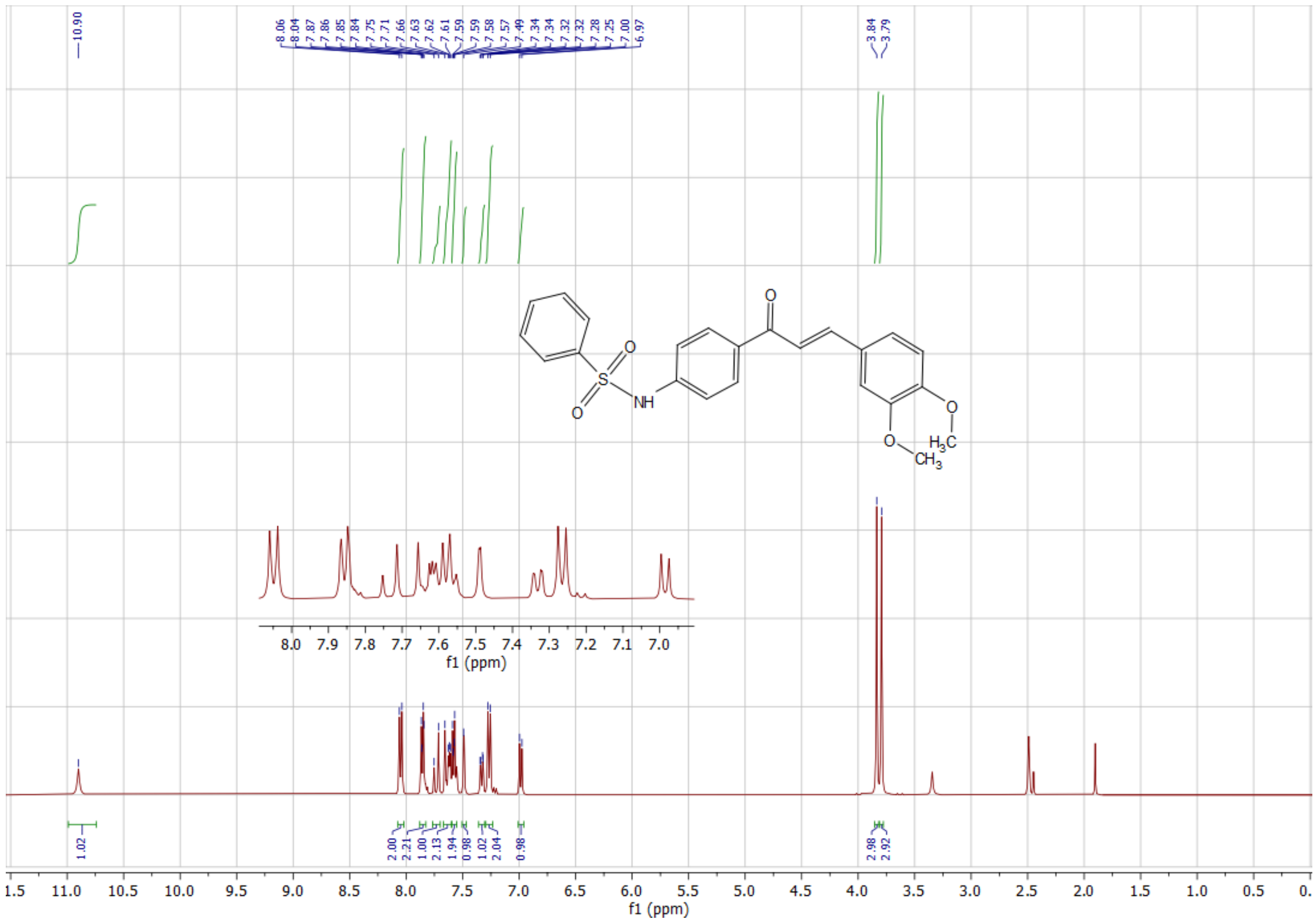

Fig. S25: ^{1}H-NMR spectrum (400 MHz, DMSO-d$_6$) of compound **12g**

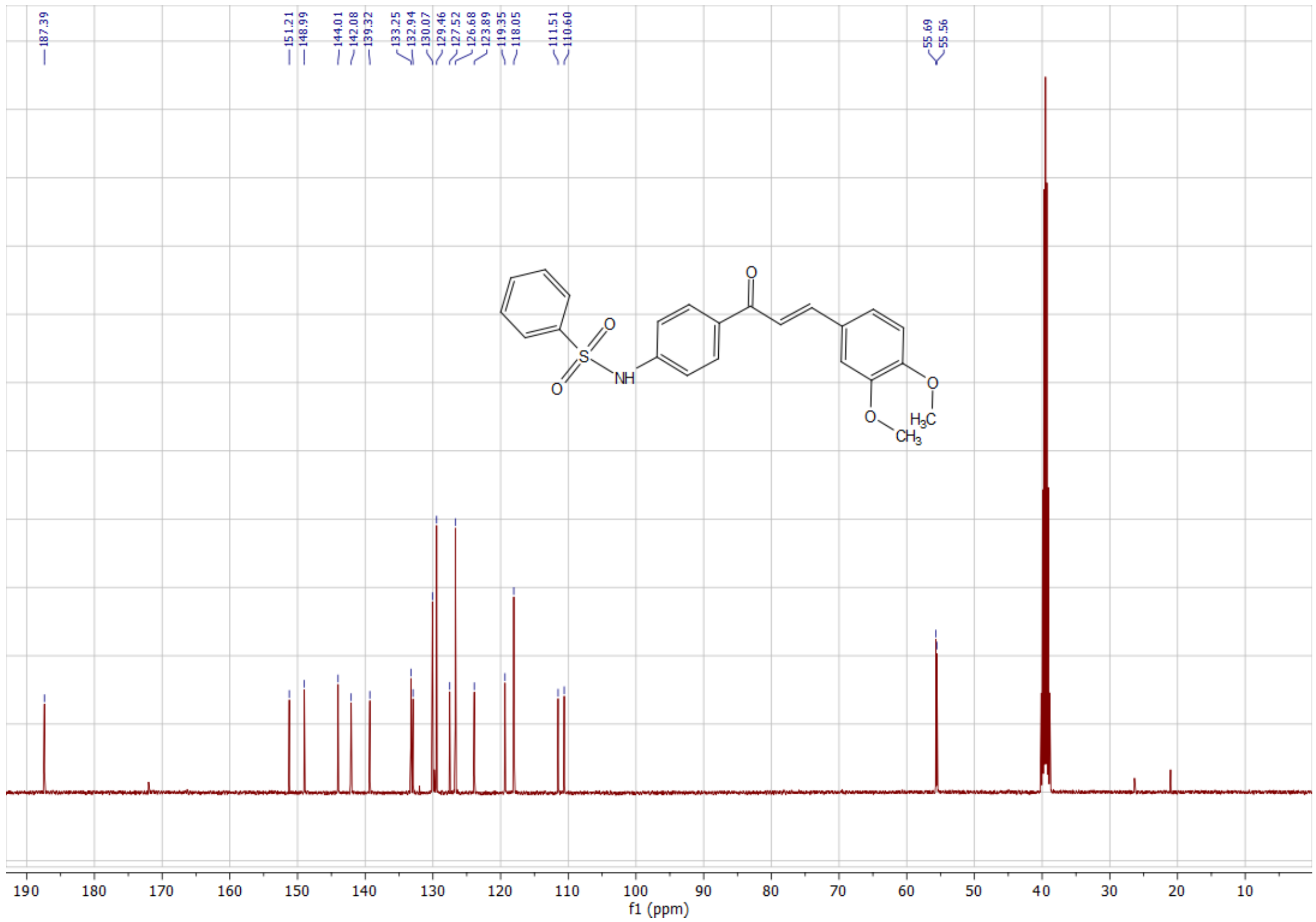

Fig. S26: ^{13}C -NMR spectrum (100 MHz, DMSO-d_6) of compound **12g**

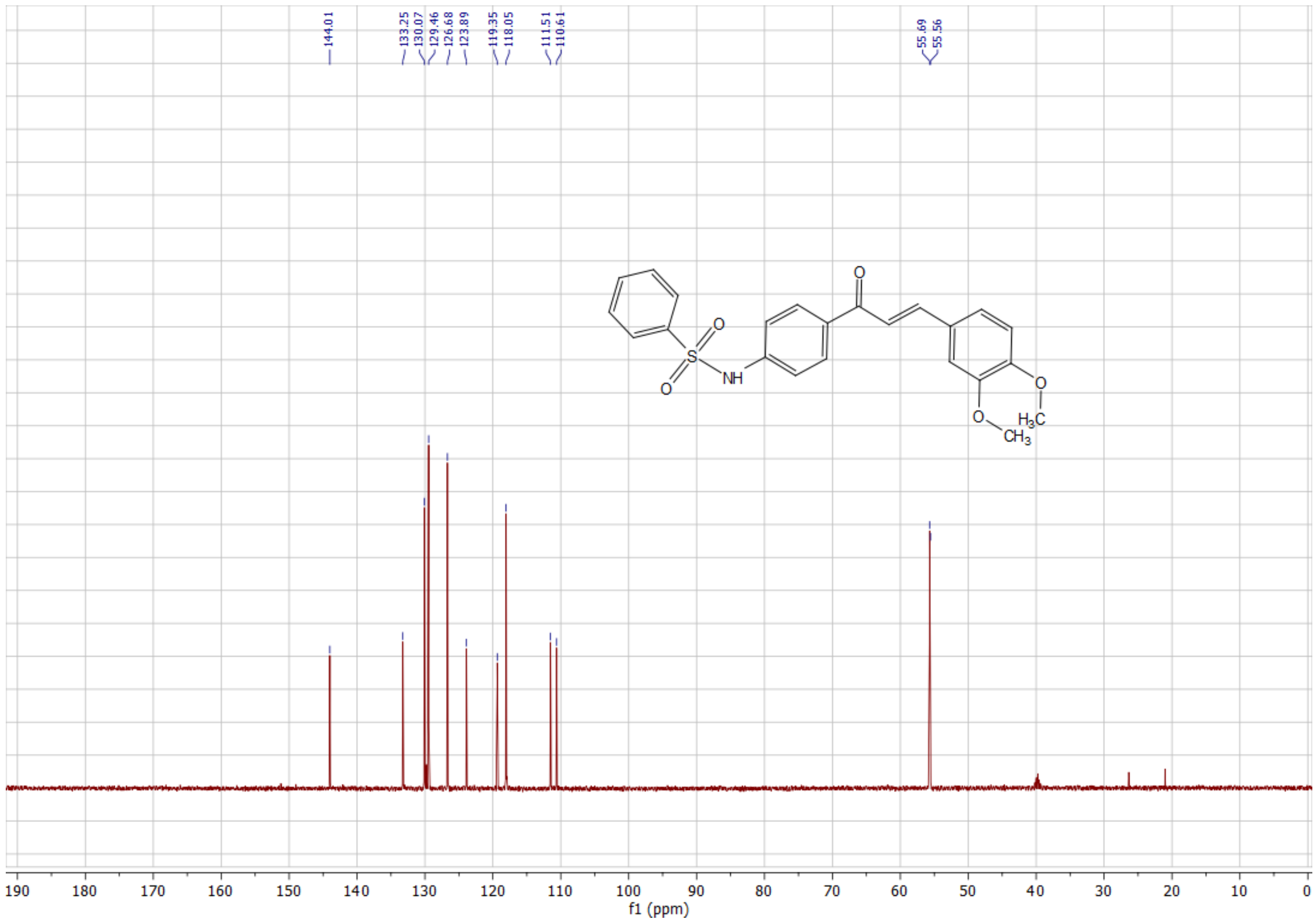

Fig. S27: DEPT NMR spectrum (100 MHz, DMSO-d_6) of compound **12g**

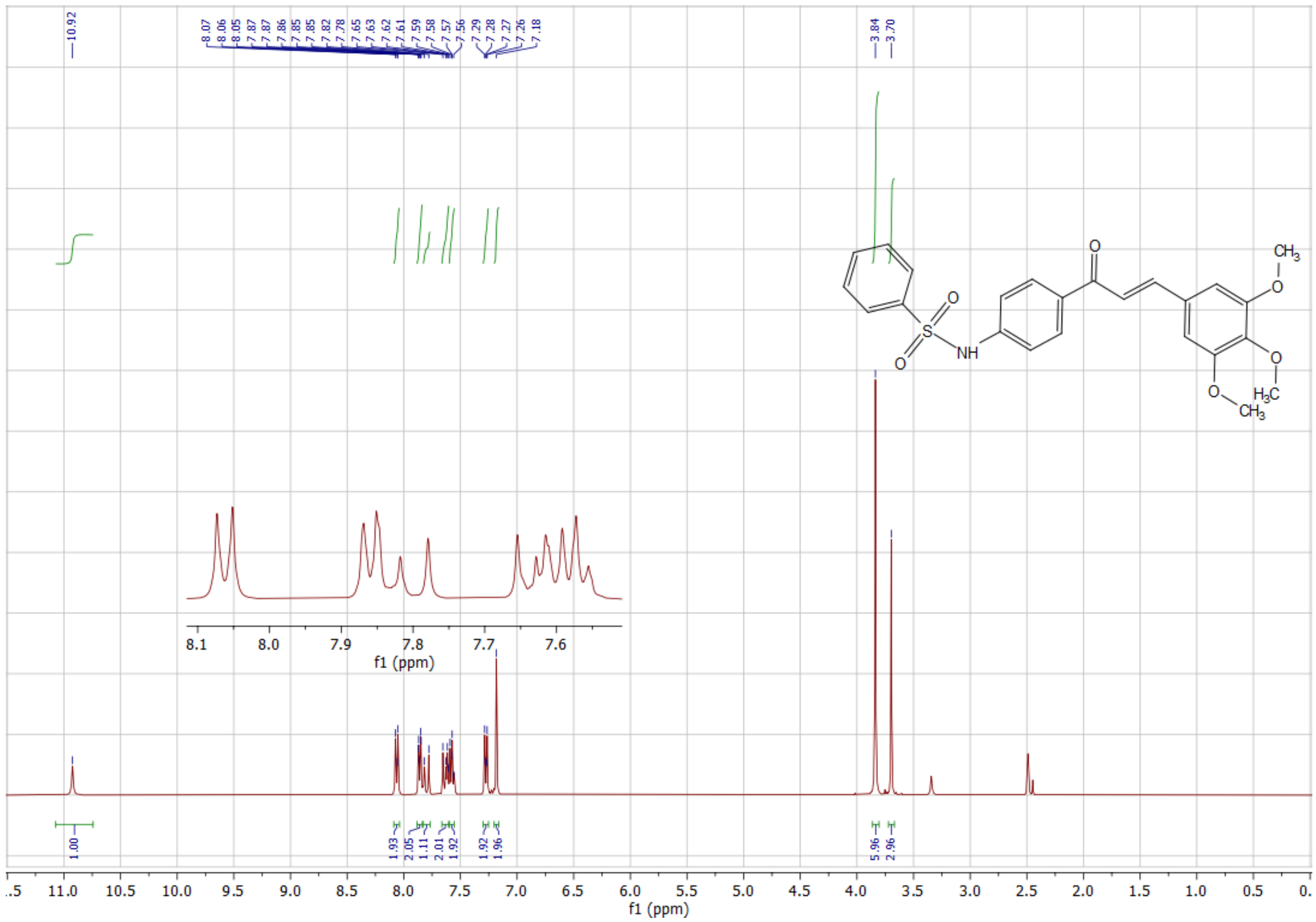

Fig. S28: ^{1}H -NMR spectrum (400 MHz, DMSO-d_6) of compound **12h**

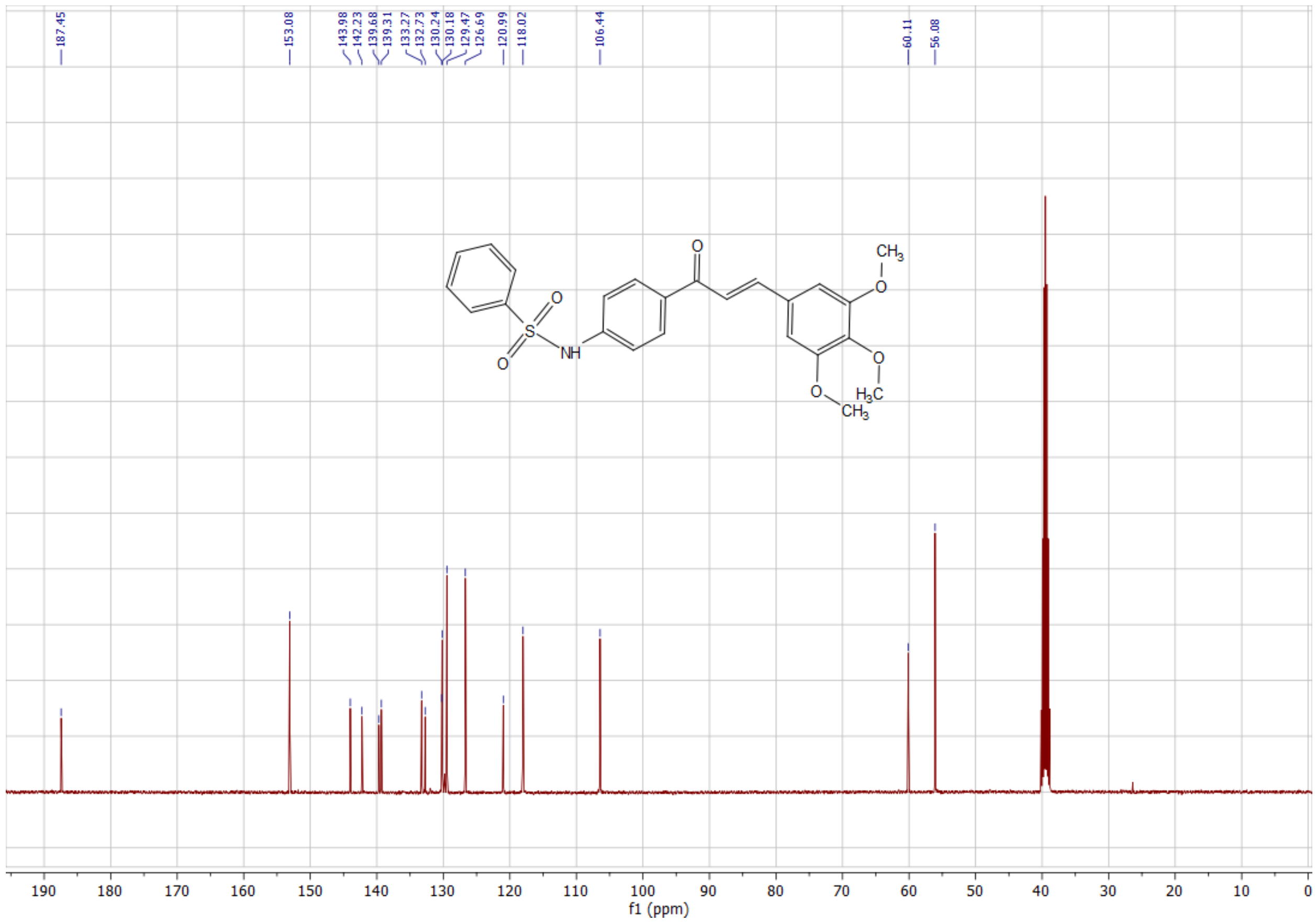

Fig. S29: ^{13}C -NMR spectrum (100 MHz, DMSO-d_6) of compound **12h**

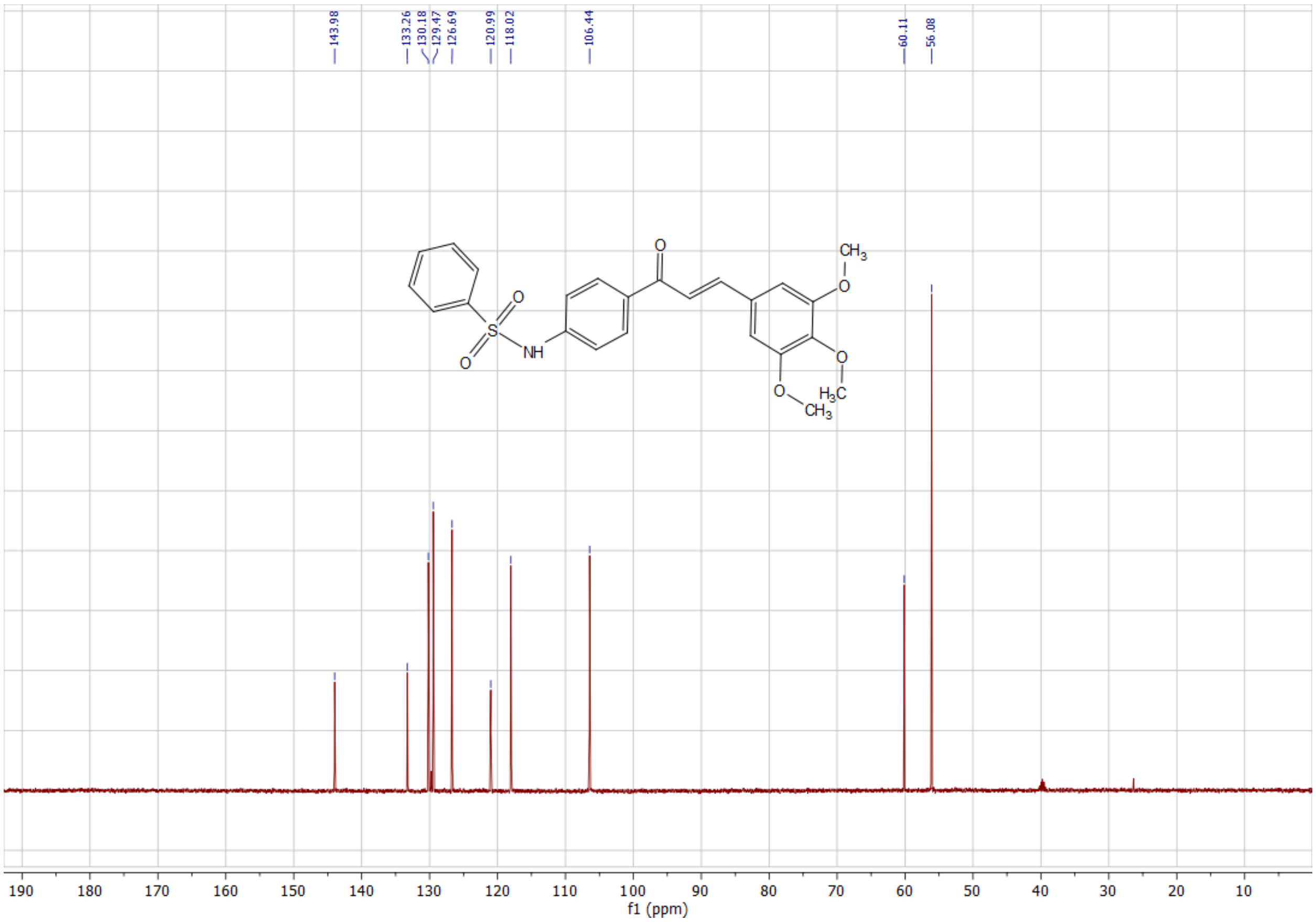

Fig. S30: DEPT NMR spectrum (100 MHz, DMSO-d_6) of compound **12h**

Table S1: Molecular Docking Results of Synthesized Compounds against *E. coli* DHPS (PDB ID: 1AJ0)

Compounds	Binding affinity (kcal/mol)	H-bonding	Electrostatic and Hydrophobic interactions	Vander Waals interactions
12a	-8.8	Gly191 (dist. 1.94097Å, 134.691^{o}), Arg255 (dist. 1.94731Å, 161.095^{o}), Pro145 (dist. 2.14543Å, 128.45^{o}), Thr147 (dist. 2.97981Å, 118.438^{o}), Ser222 (3.62624Å, carbon H-bond), Arg63 (dist. 3.15555Å, pi-donor H-bond)	Arg63, Phe190, Ala151, Arg220, Ile20, His257, Pro64, Lys221	Asn144, Lys146, Gln149, Glu150, Asn193, Asn22, Lys192, Gly189, Thr62, Ser219
12b	-8.9	Gly191 (dist. 1.93767Å, 134.808^{o}), Ser222 (dist. 2.80547Å, 154.167^{o}), Arg255 (dist. 1.84485Å, 168.687^{o}), Pro145 (dist. 2.14721Å, 129.471^{o}), Thr147 (dist. 2.97697Å, 117.725^{o}), Ser222 (dist. 3.5696Å, carbon H-bond), Arg63 (dist. 3.12374Å, pi-donor H-bond)	Arg63, Phe190, Ala151, Ile20, His257, Pro64, Lys221	Asn144, Lys146, Gln149, Glu150, Asn193, Lys192, Gly189, Thr62
12c	-8.7	Gly1491 (dist. 2.07193Å, 131.432^{o}), Arg225 (dist. 2.06307Å, 150.662^{o}), Thr147 (dist. 2.60196Å, 114.145^{o}), Ser222 (dist. 3.60809Å, carbon H-bond)	Lys221, Phe190, Ala151, His257, Pro64, Arg63	Asn193, Lys192, Gly189, Ile117, Thr162, Ile20, Asn22, Arg220, Ser219, Asn144, Gln149, Glu150, Pro145

12d	-8.8	Gly191 (dist. 1.99272Å, 111.39^o), Ser222 (dist. 2.25989Å, 155.559^o), Pro145 (dist. 3.43387Å, 161.143^o) Ser222 (dist. 3.75233Å, carbon H-bond), Arg63 (dist. 2.61046Å, pi-donor H-bond)	His257, Arg63, Phe190, Ala151, Pro64, Lys221	Asn193, Glu150, Gln149, Asn144, Lys146, Asn22, Ile20, Gly189, Lys192
12e	-8.5	Arg235 (dist. 1.8012Å, 153.703^o), Arg235 (dist. 3.04301Å, 124.631^o), Arg220 (dist. 2.54585Å, 145.493^o), Gln149 (dist. 3.48319Å, carbon H-bond), Gln142 (dist. 3.57148Å, carbon H-bond), Asn144 (dist. 3.49923Å, carbon H-bond), Thr147 (dist. 3.42292Å, carbon H-bond)	Arg63, Ala151, Lys221, Pro64, Pro232	Thr62, Glu150, Pro145, Gly189, Phe190, Gly191, Pro152, Ser222
12f	-8.6	Gly191 (dist. 1.91729Å, 136.996^o), Arg255 (dist. 1.99156Å, 149.179^o), Arg255 (dist. 2.13396Å, 142.941^o), Pro145 (dist. 2.30435Å, 120.899^o), Thr147 (dist. 2.84335Å, 123.994^o), His257 (dist. 3.26888Å, carbon H-bond)	Phe190, Ile20, Arg220, Lys221, His257, Pro64, Arg63, Als151	Glu150, Gln149, Gly189, Ser222, Thr62, Asn22, Ser219, Asn144, Lys192
12g	-8.7	Ser222 (dist. 2.3229Å, 136.321^o), Arg255 (dist. 2.08697Å, 170.182^o), Arg255 (dist. 2.67221Å, 109.439^o), His257 (dist. 3.50187Å, carbon H-bond)	Arg63, Lys221, Ala151, Ile20, Ile117, His257, Phe190, Pro64,	Glu150, Gln149, Gly189, Thr62, Asn22, Gln142, Pro152, Gly191, Thr147, Asn144, Lys192

12h	-8.8	Asn22 (dist. 2.62101Å, 124.659^o), Gly191 (dist. 2.11921Å, 136.269^o), Arg255 (dist. 1.92912Å, 128.564^o),<br>Thr147 (dist. 3.05992Å, 106.317^o),<br>Glu60 (dist. 3.78262Å, carbon H-bond),<br>Ser61 (dist. 3.30467Å, carbon H-bond)	Phe190, Ilee117, Arg63, Phe190, Pro64, Ala151	Lys192, Gln149, Asn144, Pro145, Asp196, Thr62, Lys221, Ser222, Gly189
SMX	-7.1	Thr62 (dist. 2.17886Å, 149.208^o),<br>Thr62 (dist. 2.25916Å, 113.728^o),<br>Arg255 (dist. 2.70125Å, 95.164^o), Arg255 (dist. 2.42418Å, 129.469),<br>Arg255 (dist. 2.79272Å, 93.557^o), Arg255 (dist. 2.0574Å, 137.875^o),<br>Thr62 (dist. 2.65946Å, 105.472^o)	Arg63, Arg255, Phe190, Ile117, Met139, Lys221	Gly217, Asn115, Arg220, Ser219, His257, Ser61, Asn22
Co-crystallized ligand	-5.0	Asn22 (dist. 2.3476Å, 129.124^o),<br>Thr62 (dist. 2.89385Å, 118.783^o),<br>Arg63 (dist. 2.2475Å, 127.733^o),<br>Arg255 (dist. 2.1.85859Å, 154.184^o),<br>Arg255 (dist. 2.32718Å, 135.872^o),<br>Arg63 (dist. 3.10706Å, carbon H-bond)	Arg63, Arg255, His257, Lys221	Phe190, Ile20

Table S2: Molecular Docking Results of Synthesized Compounds against DHPS of *S. aureus* (PDB ID: 1AD4)

Compounds	Binding affinity (kcal/mol)	H-bonding interactions	Electrostatic and Hydrophobic interactions	Vander Waals interactions
12a	-7.0	Gln105 (dist. 2.7054Å, 108.64°), Arg204 (dist. 2.11719Å, 158.29°), Arg239 (dist. 2.03892Å, 129.41°)	Arg52, Lys203, Arg204, His55	Asn11, Ser50, Val49, Thr51, Asp84, Phe172, His241, Ser201, Gly171
12b	-6.9	Gln105 (dist. 2.54256Å, 109.89°), Arg239 (dist. 2.45116Å, 124.62°), Ser201 (dist. 3.3241Å)	Arg204, His55, Lys203	Asp84, Thr51, Phe172, Gly171, Arg52, Lys207, Arg219, His241, Asn11, Val49, Ser50
12c	-6.7	Arg52 (dist. 2.30816Å, 106.42°), Gln105 (dist. 2.54458Å, 107.00°), Asp84 (dist. 3.22344Å)	Arg52, Phe172, Met128, His55	Gly54, Pro53, Ser50, Val49, Arg239, Lys203, Ser201, Arg202, Arg204
12d	-7.5	Gln105 (dist. 2.69939Å, 106.30°), Arg239 (dist. 2.33736Å, 125.20°), Ser201 (dist. 3.36183Å)	Arg204, His55, Lys203	Asp84, Thr51, Phe172, Arg52, Lys207, Arg202, Arg219, His241, Asn11, Val49, Ser50
12e	-6.8	Gln105 (dist. 2.89867Å, 93.41°), Arg239 (dist. 2.93755Å, 116.11°), Arg239 (dist. 2.08146Å, 128.20°)	Arg52, Arg239, Phe172, Arg204, Lys207, Lys203, Met128, Ala199	Asp167, Asn103, Asp84, Ser50, Thr51, His241
12f	-7.0	Asn11 (dist. 2.26954Å, 139.624°), Arg239 (dist. 2.44891Å, 123.85°),	His241, Arg52, His55, Lys203	Phe172, Gln105, Thr51, Val49, Ser50, Ile9, Arg202, Arg204,

		Arg239 (dist. 2.05524Å, 131.551°), Lys203 (dist. 3.40177Å, carbon H-bond), Pro53 (dist. 3.55609Å, carbon H-bond)		Gly54
12g	-6.9	Gln105 (dist. 2.82466Å, 103.443°)	Arg52, Arg239, Phe172, Arg204, Lys203, Met128, Ala199	Asp84, Lys207, Arg219, Arg202, Ser201, His241, Ser50, Val149
12h	-6.3	Arg204 (dist. 2.54593Å, 109.588°), Arg219 (dist. 2.45094Å, 115.637°), Arg239 (dist. 2.23726Å, 147.179°), Asn11 (dist. 2.41005Å, 145.362°), Ser50 (dist. 3.63781Å, carbon H-bond), Lys203 (dist. 3.33162Å, carbon H-bond)	Arg239, Arg204, Pro216, Lys203	Val12, Arg52, Val149, Ser50, His55, Phe172, His241, Arg202
SMX	-6.6	Arg52 (dist. 2.48616Å, 142.378°), Arg52 (dist. 2.03817Å, 159.916°), Arg239 (dist. 2.27122Å, 162.864°), Lys203 (diat. 3.76065Å, carbon H-bond), Lys203 (dist. 3.58297Å, carbon H-bond)	Arg239, Phe172, Met128, Ala199	His55, Val49, Asn11, Ser50, Asp167, Asn103, Gln105, Asp84
Co-crystallized ligand	-6.9	Arg204 (dist. 2.0346Å, 153.377°), Arg239 (dist. 2.89193Å, 112.247°), Asp84 (dist. 3.1042Å) Ser50 (dist. 2.96003Å)	Arg52, Arg219, Phe172	His241, Ile9, Gln105, Thr51, Lys203

Table S3: Molecular Docking Results of Synthesized Compounds against MPO of (PDB ID: 1DNU)

Compound	Binding affinity (kcal/mol)	H-bonding interactions	Electrostatic and Hydrophobic interactions	Vander Waals interactions
12a	-9.5	Arg333 (dist. 3.08227Å, 112.527°), Tyr334 (dist. 3.67334Å)	Arg333, Glu102, Phe407, Gly90, Gly91, Ile339, Leu338, Arg424, Tyr334, His335	Tyr296, Phe332, Ser149, His95, Thr100, Phe99, Leu406, Glu241, Leu417, Arg239, Thr329, Asp94, Gln91
12b	-9.5	Arg333 (dist. 2.91113Å, 113.353°)	Arg333, Phe407, His336, Gly90, Gly91, Met87, Ile339, Leu338, Arg239, Phe99, Tyr334,	Tyr296, Phe332, Gly335, Phe366, Glu242, Leu420, Leu406, Leu417, Asn421, Thr329, Asp94, Gln91
12c	-9.3	Arg424 (dist. 2.03242Å, 155.186°), His95 (dist. 2.47861Å, 139.411°)	Asp94, His95, His336, Leu420, Met411, Leu415, Arg239, Arg333	Glu102, Phe147, Phe146, Pro145, Glu116, Leu417, Phe407, Phe332, Gly90, Gly335, Gln91, Glu242, Phe366
12d	-9.8	Arg333 (dist. 2.50916Å, 113.243°), Arg333 (dist. 2.69172Å, 111.395°), His336 (dist. 3.08925Å, 91.711°)	Pro145, Glu102, Leu417, His95, His336, Phe332, Leu415, Leu420	Met411, Leu406, Phe407, Ile339, Gly335, Gly90, Asp94, Asn421, Arg424,

				Phe146, Glu116, Phe147, Gln91, Arg239, Glu242
12e	-9.6	Arg239 (dist. 2.2.8075Å, 107.33^{o}), Arg333 (dist. 2.91579Å, 100.66^{o}), His336 (dist. 2.2168Å, 154.71^{o}), Thr238 (dist. 3.47541Å, carbon H-bond), Thr238 (dist. 3.4516Å, carbon H-bond)	Asp94, His95, His336, Phe366, Leu417, Arg239, Arg333	Leu406, Leu420, Phe407, Glu242, Gln91, Phe332, Thr329, Phe99
12f	-9.4	Arg333 (dist. 3.00834Å, 119.927^{o}), Arg424 (dist. 2.59088Å, 126.924^{o}), Arg424Å (dist. 2.48096Å, 122.731^{o}), Met87 (dist. 3.59754Å)	Arg333, Glu102, Leu420, His336, Gly90, Gln91, Met87, Ile339, His336, Leu415	Gly335, Phe332, Leu417, Phe407, Phe147, Phe146, Pro145, Asn421, Arg239, Thr329, Asp98, Asp94, Gln91
12g	-9.6	Gln91 (dist. 2.3755Å,127.893^{o}), Arg333 (dist. 2.73358Å,139.278^{o}), Asn421 (dist. 3.0615Å, 116.959^{o}), Arg424 (dist. 2.16915Å, 133.763^{o}), Arg424 (dist. 2.53222Å,125.4^{o}), Gly90 (dist. 3.52615Å)	Leu420, Met87, His336, Leu338, Phe332	Leu415, Phe407, Leu406, Gly90, Asp94, His95, Leu417, Glu242
12h	-8.8	Arg424 (dist. 2.10129Å, 145.796^{o}), Glu102 (dist. 3.66543Å)	Met87, His336, His95, Gly90, Gln91, Leu420,	Gly335, Ile339, Leu417, Glu242, Arg239, Phe407, Leu415, Pro145, Glu102, Phe146, Phe147, Asp94,

				Arg333, Gln91,
AA	-6.1	Asp94 (dist. 2.3541Å, 110.05)<br>Arg333 (dist. 1.90236Å, 139.34^{o}),<br>Arg333 (dist. 2.75631Å, 137.21^{o}),<br>Arg239 (dist. 2.84079Å, 105.41^{o}),<br>Asp98 (dist. 2.87085Å, 103.04^{o}),<br>His95 (dist. 2.75611Å, 126.14^{o}),<br>Thr100 (dist. 3.70517Å),<br>Arg239 (dist. 3.65796Å)	-	Phe99, Gln91, Thr329

Table S4: Molecular Docking Results of Synthesized Compounds against COX-2 (PDB ID: 5IKR)

Compounds	Binding affinity (kcal/mol)	H-bonding interactions	Electrostatic and Hydrophobic interactions	Vander Waals interactions
**12a**	-8.6	Tyr (dist. 2.23499Å, 118.67^{o}), Ser530 (dist. 1.98065Å, 111.86^{o}), Val523 (dist. 3.63834Å, carbon H-bond)	Arg120, Ala527, Phe205, Tyr385, Phe209, Val116, Val228, Tyr255, Leu352, Val349, Leu534	Leu359, Leu531, Met522, Gly526, Trp387, Tyr348, Thr206, Val344, Gly227, Ser353, Gly533, Phe381
**12b**	-8.6	Ser530 (dist. 1.96958Å, 151.29^{o}), Glu524 (dist. 3.49403Å, carbon H-bond)	Arg120, Val349, Ala527, Met522, Trp387, Gly526, Val89, Leu384, Leu531, Val116, Leu352	Val523, Ser353, Leu359, Tyr355, Glu524, Leu93, His90, Pro86, Tyr115, Tyr348, Tyr385, Phe381
**12c**	-8.4	Arg120 (dist. 2.38451Å, 129.66^{o}), Arg120 (dist. 2.35887Å, 113.61^{o}), Tyr355 (dist. 2.7230Å, 119.73^{o}), Tyr355 (dist. 3.19856Å, carbon H-bond)	Leu94, Val116, Tyr355, Ile112, Trp100, Leu359, Ala527, Val89	Tyr115, Leu531, Val349, Ser530, Tyr348, Tyr385, Phe381, Trp387, Leu352, Gly526, Phe518, Val523, Met522, leu384
**12d**	-8.9	Arg120 (dist. 2.20411Å, 144.49^{o}), Arg120 (dist. 2.22893Å, 131.68^{o}), Tyr355 (dist. 2.09955Å, 126.97)	Met522, Leu93, Val116, Tyr355, Ala527, Ile112, Leu531, Leu352, Val523, Gly526	Leu384, Trp387, Ser530, Val349, Leu359, Val89, Tyr115, Trp100

**12e**	-9.5	Ser530 (dist. 2.26956Å, 134.30^{o})	Arg120, Ala527, Met522, Trp387, Gly526, Val89, Tyr115, Val349, Val116, Leu352	Ser353, Leu359, Tyr355, Ser119, Val523, Leu531, Leu93, Leu384, Phe381, Tyr385, Tyr348
**12f**	-8.5	Ser530 (dist. 2.10235Å, 139.72^{o})	Val349, Ala527, Met522, Trp387, Leu93, Val116, Leu531, Leu352	Tyr385, Phe518, Tyr348, Val523, Leu384, Phe381, Ser353, Arg120, Tyr355, Leu359, Phe357, Val89, Gli526, Tyr115, Ile112
**12g**	-9.4	Ser530 (dist. 2.1331Å, 151.17^{o})	Arg120, Val116, Val349, Ala527, Met522, Trp387, Gly526, Val89, Tyr115, Leu531, Leu352	Leu93, Ser353, Tyr355, Leu359, Tyr348, Tyr385, Phe381, Leu384, Val523
**12h**	-9.1	Ser530 (dist. 2.4011Å, 130.06^{o}), Ala527 (dist. 3.71223Å, carbon H-bond)	Arg120, Val116, Ala527, Met522, Trp387, Gly526, Val89, Leu93, Val349, Leu352	Val523, Ser353, Leu359, Tyr355, Ser119, Tyr115, Leu531, Ile112, Tyr385, Phe381, Leu384
**Diclo**	-8.5	Ser530 (dist. 2.36693Å, 120.12^{o}), Ala527 (dist. 2.0657Å, 134.42^{o})	Val349, Val523, Ala527, Leu352	Met522, Ser353, Tyr355, Arg120, Leu532, Gly526, Tyr348, Tyr385, Trp387, Phe387, Phe381

**Co-crystallized ligand**	-9.1	Tyr385 (dist. 2.0296Å, 156.32^{o})<br>Ser530 (dist. 1.98744Å, 155.82^{o})	Ala527, Val349, Leu531, Val116, Val349, Thr355, Leu352, Val523	Leu384, Gly526, Met522, Trp387, Phe381, Ryr348, Ser353, Arg120, Leu359

Table S5: Molecular Docking Results of Synthesized Compounds against Topoisomerase *IIα* of (PDB ID: 4FM9)

**Compounds**	**Binding affinity (kcal/mol)**	**H-bonding interactions**	**Electrostatic and Hydrophobic interactions**	**Vander Waals interactions**
12a	-8.9	Glu839 (dist. 2.21852Å), Arg713 (dist. 3.47664Å)	Arg727, Glu712, Arg713, Pro724, Tyr757, His759, Pro724,	Gly725, Lys724, Leu829, Trp840, Ser717, Phe1003, Gly1007, Arg672, Arg673, Ser709, Gln544
12b	-9.8	Leu592 (dist. 1.89303Å, 164.417^{o}), Pro593 (dist. 3.76224Å), Asn700 (dist. 3.56275Å), Phe668 (diat. 3.62783Å), Leu685 (2.54156Å)	Arg675, Leu705, Phe668, Arg672, Leu705, Pro593, Leu685, Lys701,	Asp671, Ile704, Asp683, Tyr684, Glu702, Tyr686, Tyr590, Ile577, Gln544
12c	-9	Arg672 (dist. 2.0317Å, 129.985^{o}), Arg672 (dist. 2.62793Å, 119.252^{o}), Arg673 (dist. 3.04814Å,127.591^{o}), Asp1004 (3.6488Å), Ile715 (3.74933Å), Glu712 (3.7471Å)	Arg672, Lys676, Phe1003, Pro724, Trp840,	Glu839, Arg727, His1005, Ser717, Pro716, Lys723, Gly1007, Gly679, Leu680
12d	-9.7	Leu592 (dist. 2.15793Å, 141.675^{o}), Ser591 (dist. 3.30049Å), Leu685 (dist. 2.68996Å)	Arg675, Phe668, Pro593, Leu685, Leu705,	Gln544, Ile577, Tyr590, Gln542, Tyr686, Glu702, Lys701, Asp683, Asp671, Glu682,Tyr684

12e	-9.8	Leu592 (dist. 1.85983Å, 144.584^{o}), Asp671 (dist. 3.37299Å), Phe668 (dist. 3.78507Å), Leu685 (dist. 2.76047Å)	Arg675, Phe668, Arg672, Pro593, Leu685, Leu705, Lys701	Asp543, Ser547, Gln544, Glu702, Asp683, Glu682, Tyr686, Tyr690, Ile577, Gln542, Leu685, Tyr684
12f	-8.8	Arg713 (dist. 3.48884Å), Gly1007 (3.69034Å), His759 (dist. 3.65257Å)	Phe1003, Glu712, Arg713, Pro724, Tyr757, His759, Leu829,	Lys728, Glu837, Glu839, Trp840, Ser717, Ile715, Ser709, Arg727
12g	-9.3	Leu592 (dist. 2.24584Å, 136.913^{o}), Ser591 (dist. 3.30061Å), Leu685 (dist. 2.69884Å)	Arg675, Arg672, Phe668, Pro593, Leu685, Leu705	Glu682, Glu702, Gln544, Ile577, Tyr590, Gln542, Tyr686, Tyr684, Asp683, Lys701, Asp671, Asn700
12h	-8.9	Arg672 (dist. 2.69293Å), Arg672 (dist. 2.09797Å), Arg673 (dist. 2.57738Å), Glu712 (dist. 3.59824Å), Glu712 (dist. 3.79705Å), Ile715 (dist. 3.63747Å)	Arg672, Lys676, Phe1003, Pro724, Trp840,	Arg675, Leu680,Gly1007, Glu839, Ser717, Asp1004, Pro716, Lys723Arg727
Ethoposide	-9.7	Arg672 (2.49709Å, 118.985^{o}), Lys827 (dist. 2.83034Å, 110.074^{o}), Lys827 (dist. 2.91468Å, 104.779^{o}), Met669 (dist. 3.53174Å)	Arg672, Arg673, Lys676,	Leu829, Gly1007, Glu839, Val1006, His1005, Asp1004, Trp840, Ser717, Phe1003, Arg727, Ile715, Pro724, Glu712

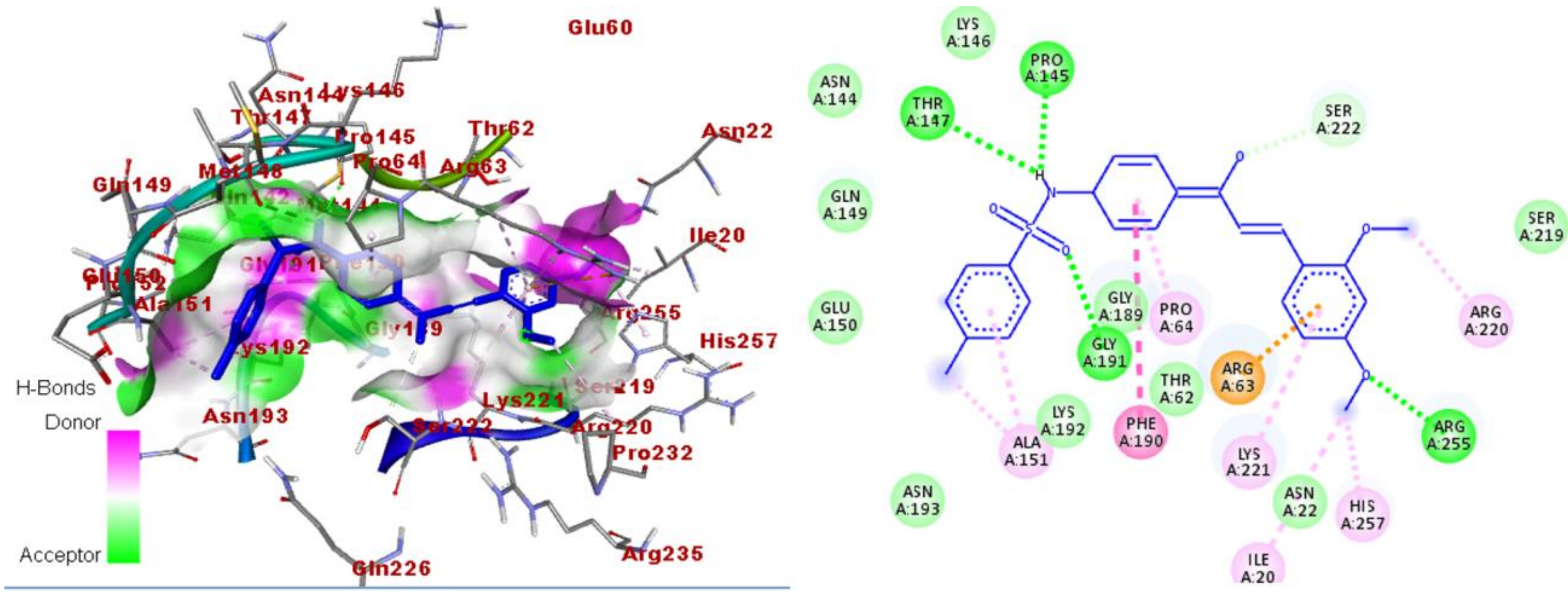

Fig. S31: 3D (left) and 2D (right) representations of the binding interactions of compound **12a** against *E. coli* DHPS (PDB ID: 1AJ0). Hydrogen bonding interactions are shown with green dashed lines; pink dashed lines highlight hydrophobic interactions, while **o**range dashed lines denote **e**lectrostatic interactions with key amino acid residues.

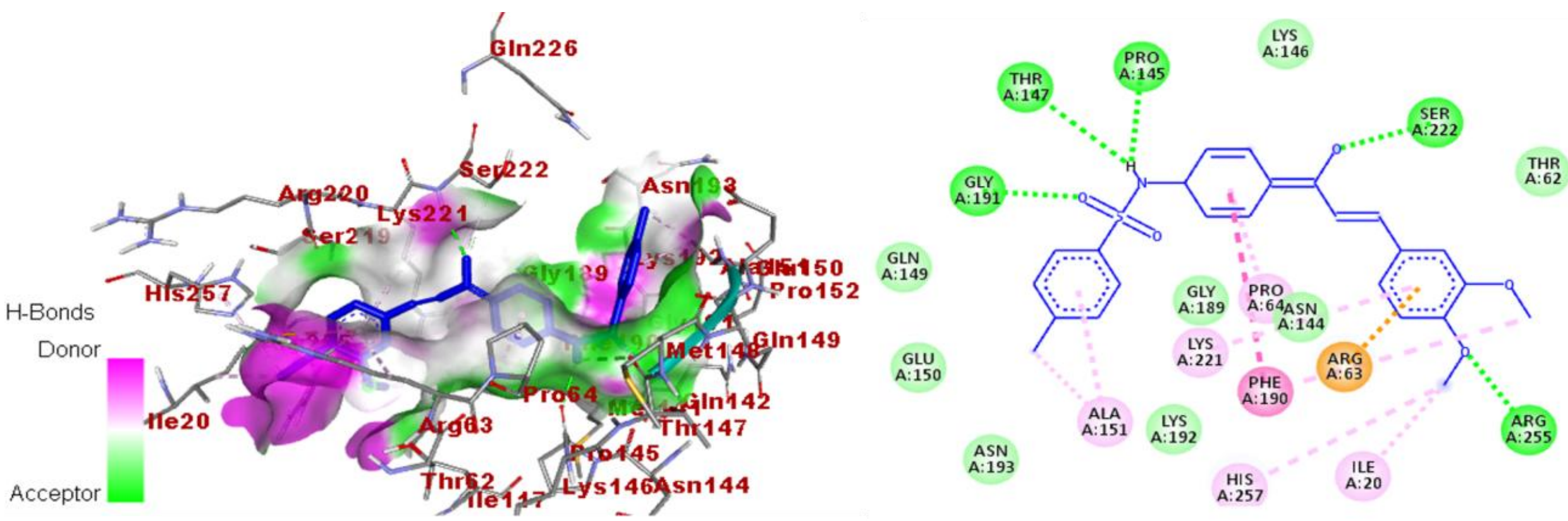

Fig. S32: 3D (left) and 2D (right) representations of the binding interactions of compound **12b** against *E. coli* DNA DHPS (PDB ID: 1AJ0)

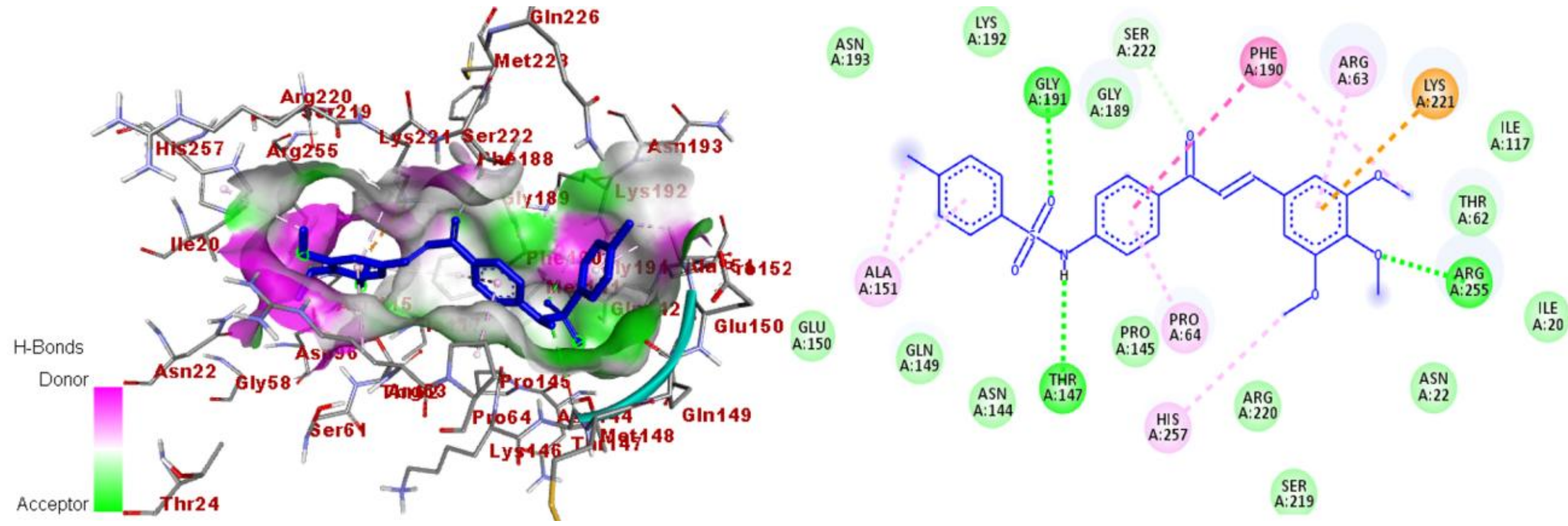

Fig. S33: 3D (left) and 2D (right) representations of the binding interactions of compound **12c** against *E. coli* DHPS (PDB ID: 1AJ0)

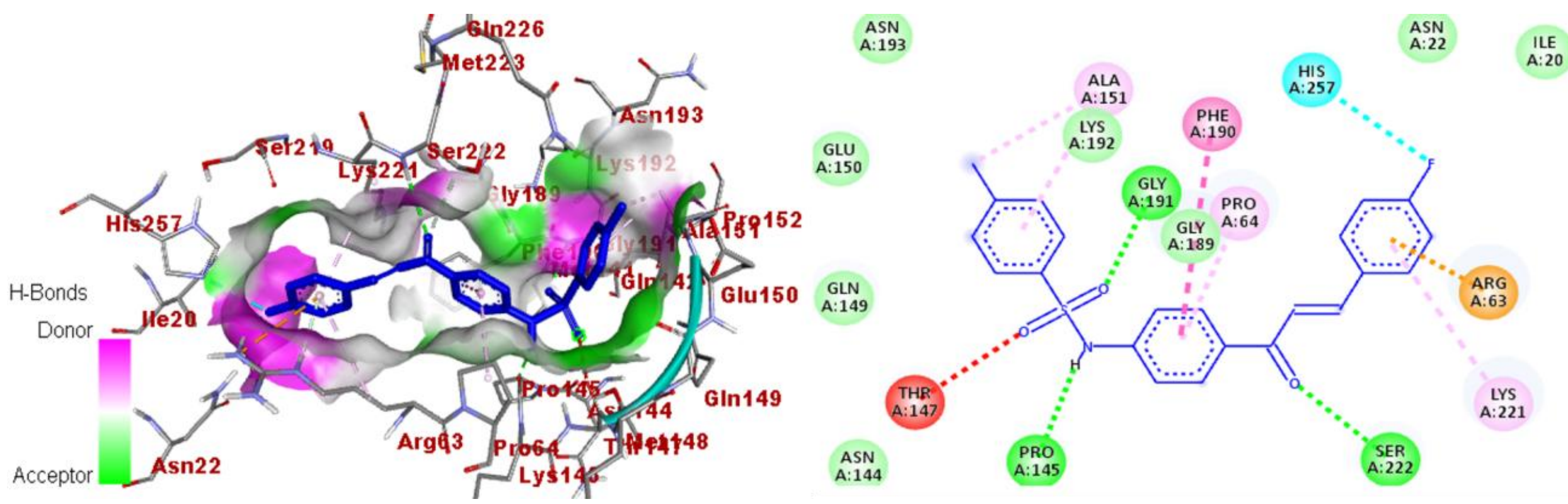

Fig. S34: 3D (left) and 2D (right) representations of the binding interactions of compound **12d** against *E. coli* DHPS (PDB ID: 1AJ0)

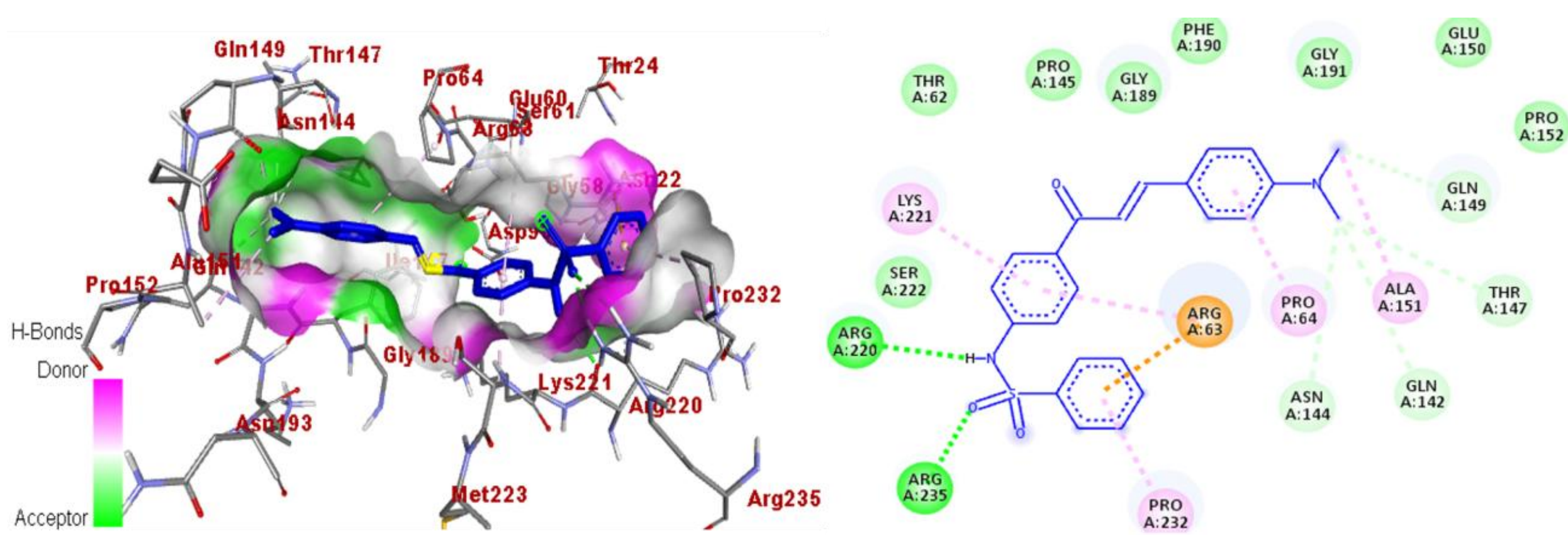

Fig. S35: 3D (left) and 2D (right) representations of the binding interactions of compound **12e** against *E. coli* DHPS (PDB ID: 1AJ0)

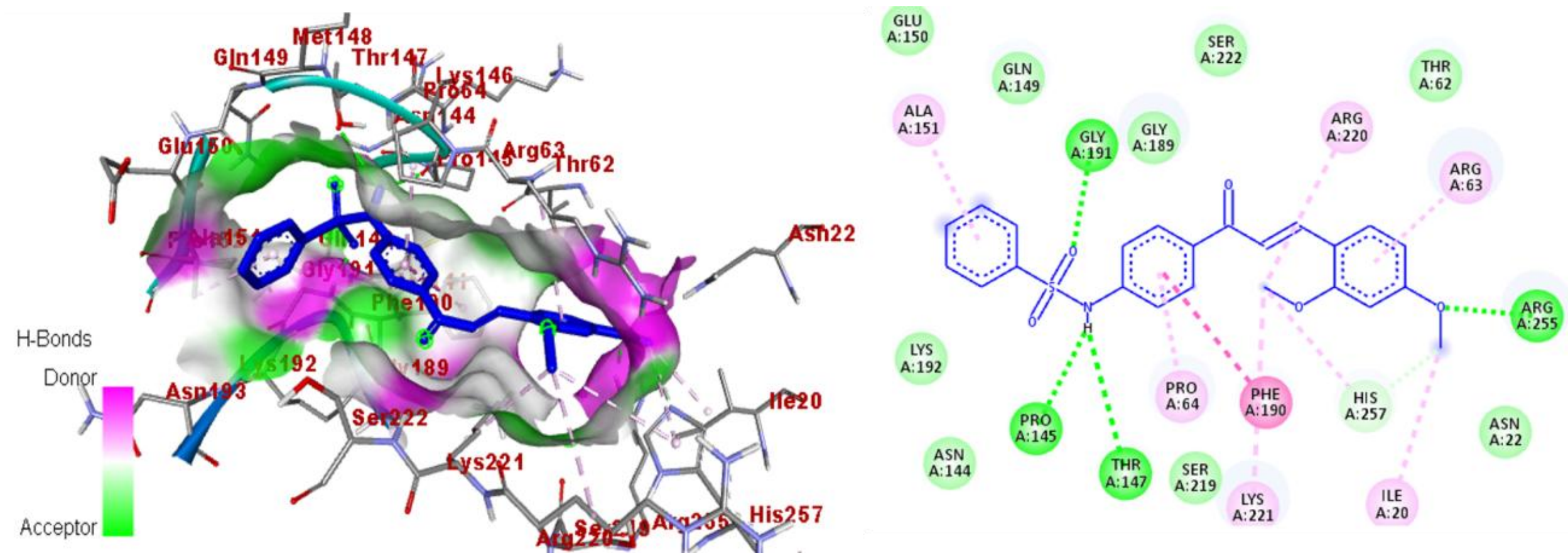

Fig. S36: 3D (left) and 2D (right) representations of the binding interactions of compound **12f** against *E. coli* DHPS (PDB ID: 1AJ0)

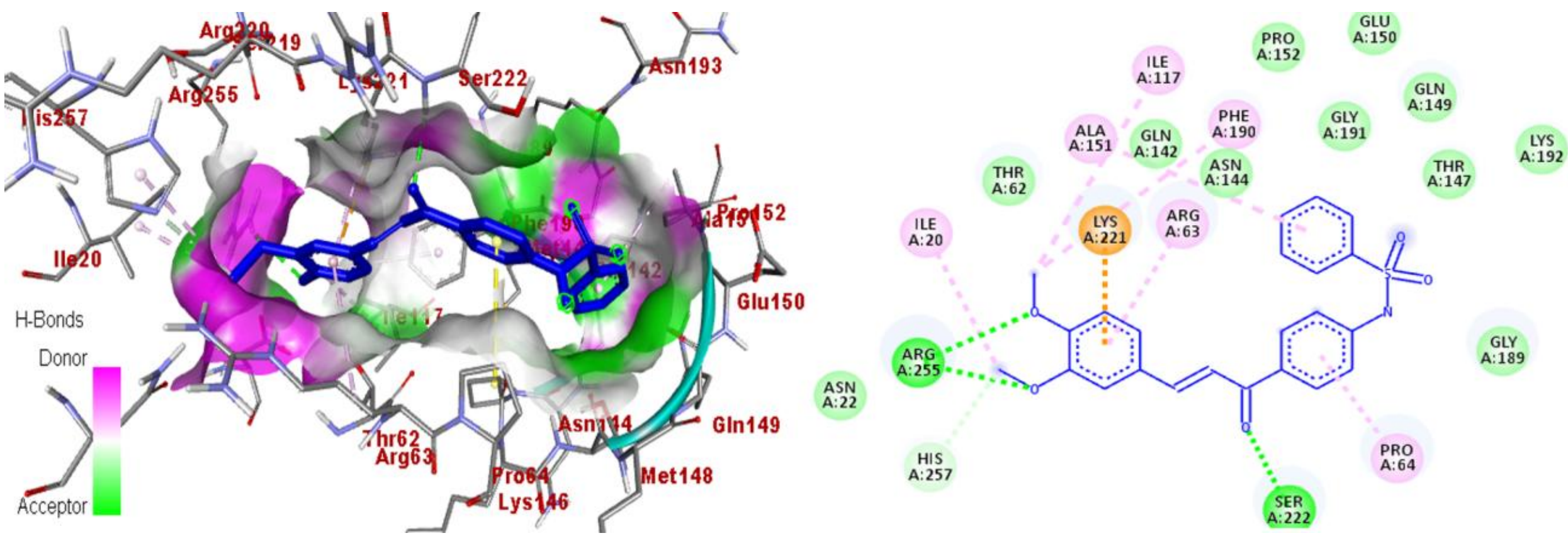

Fig. S37: 3D (left) and 2D (right) representations of the binding interactions of compound **12g** against *E. coli* DHPS (PDB ID: 1AJ0)

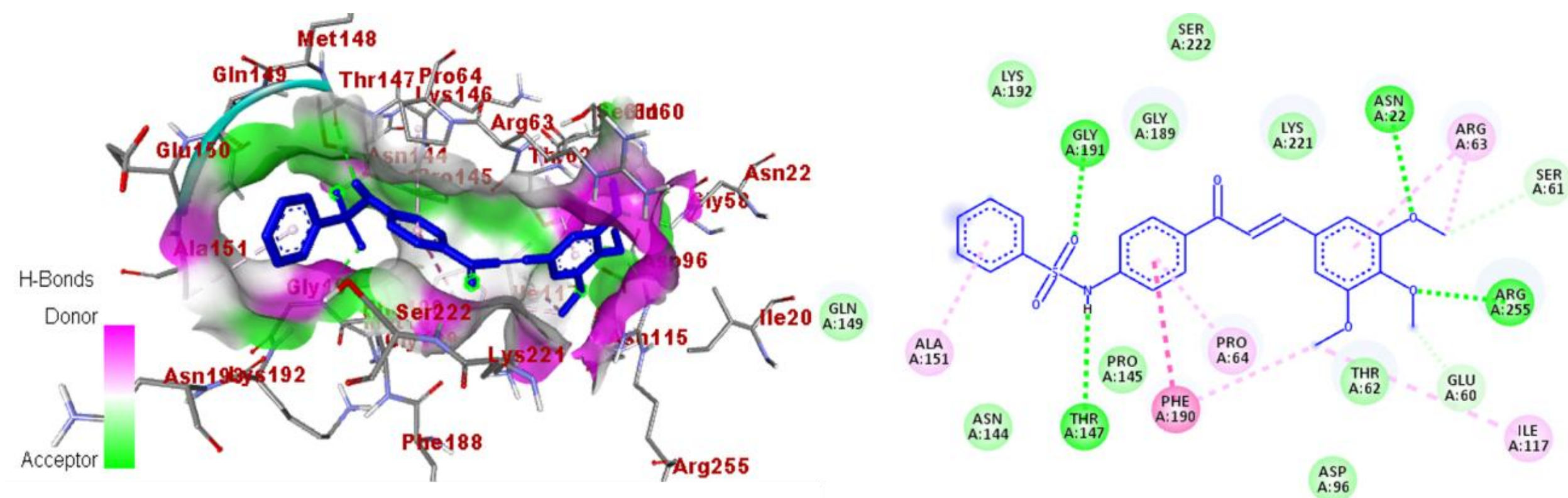

Fig. S38: 3D (left) and 2D (right) representations of the binding interactions of compound **12h** against *E. coli* DHPS (PDB ID: 1AJ0)

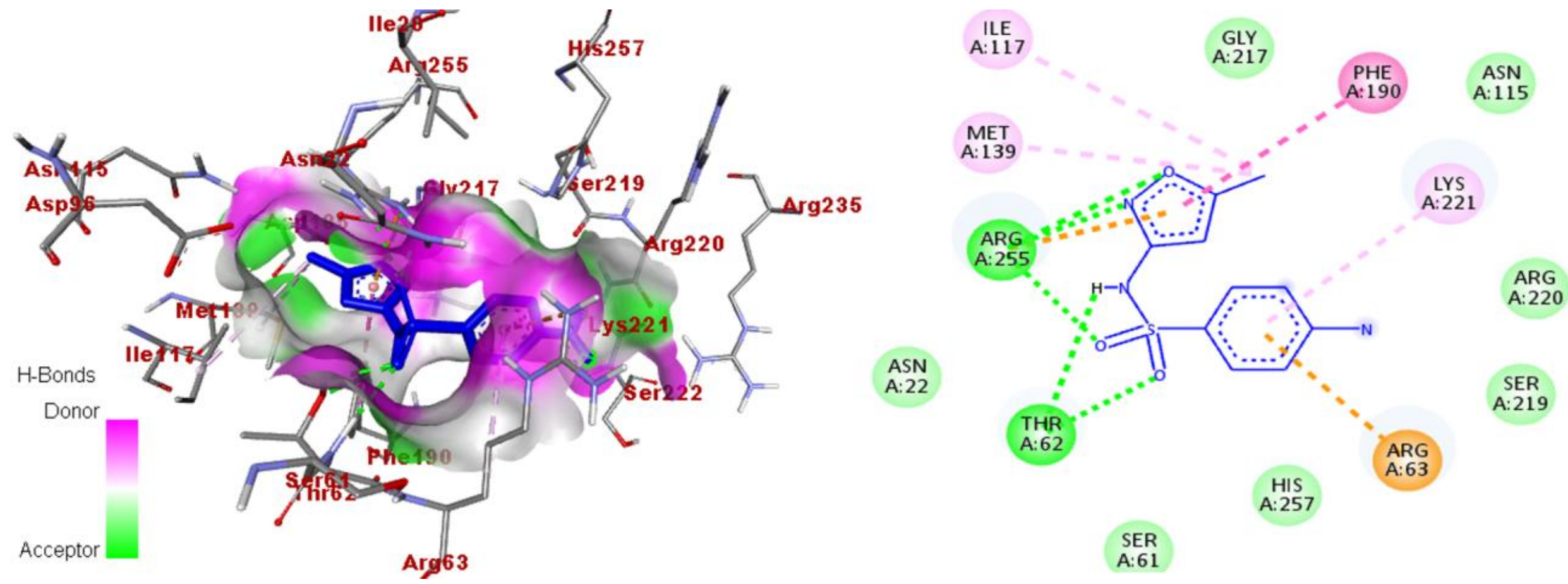

Fig. S39: 3D (left) and 2D (right) representations of the binding interactions of compound **SMX** against *E. coli* DHPS (PDB ID: 1AJ0)

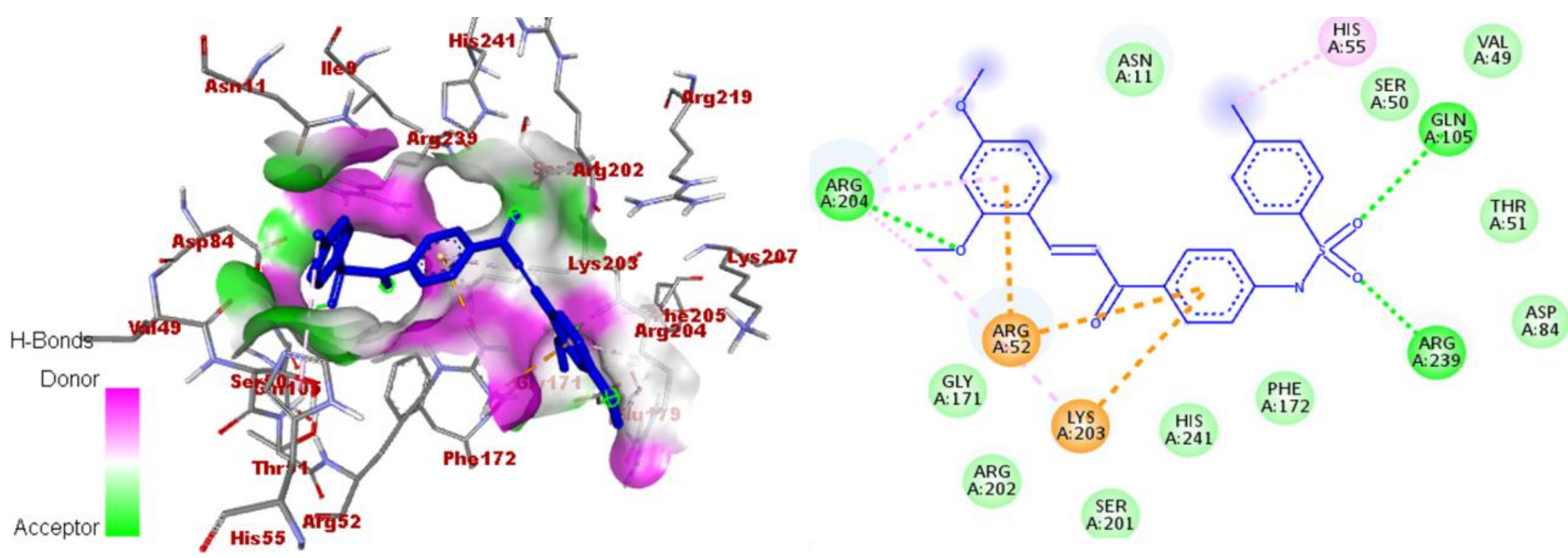

Fig. S40: 3D (left) and 2D (right) representations of the binding interactions of compound **12a** against *S. aureus* DHPS (PDB ID: 1AD4)

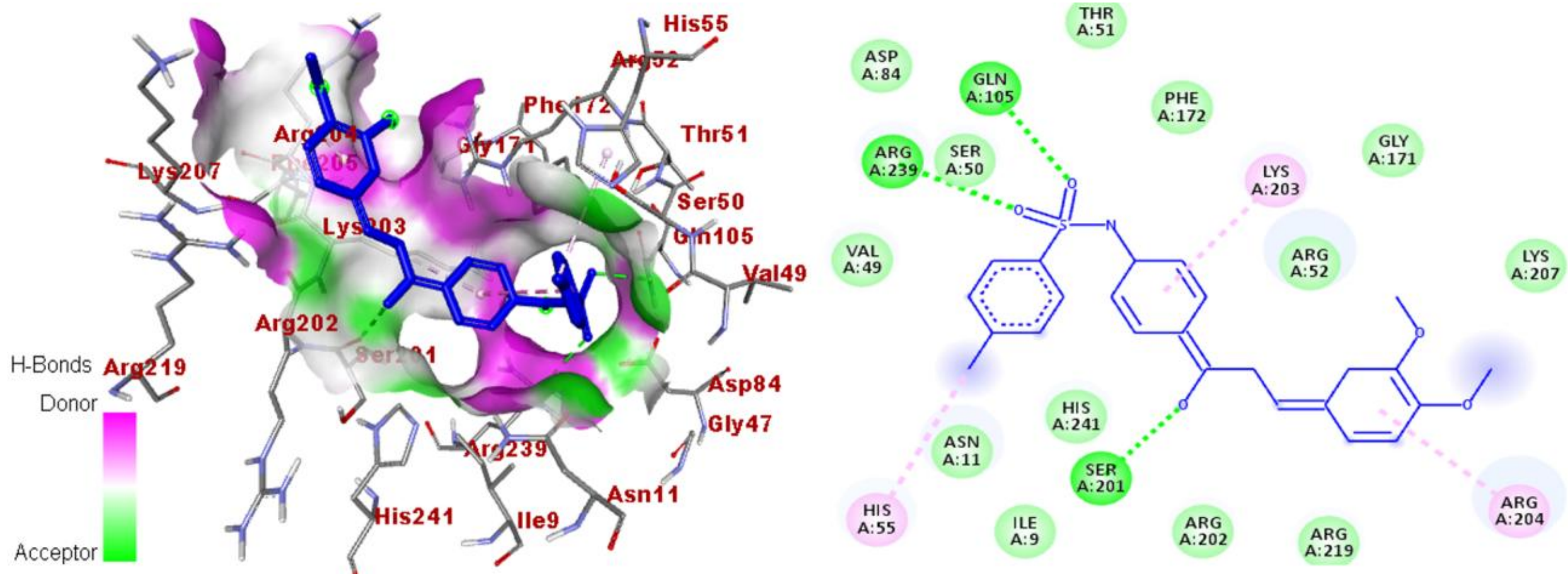

Fig. S41: 3D (left) and 2D (right) representations of the binding interactions of compound **12b** against *S. aureus* DHPS (PDB ID: 1AD4)

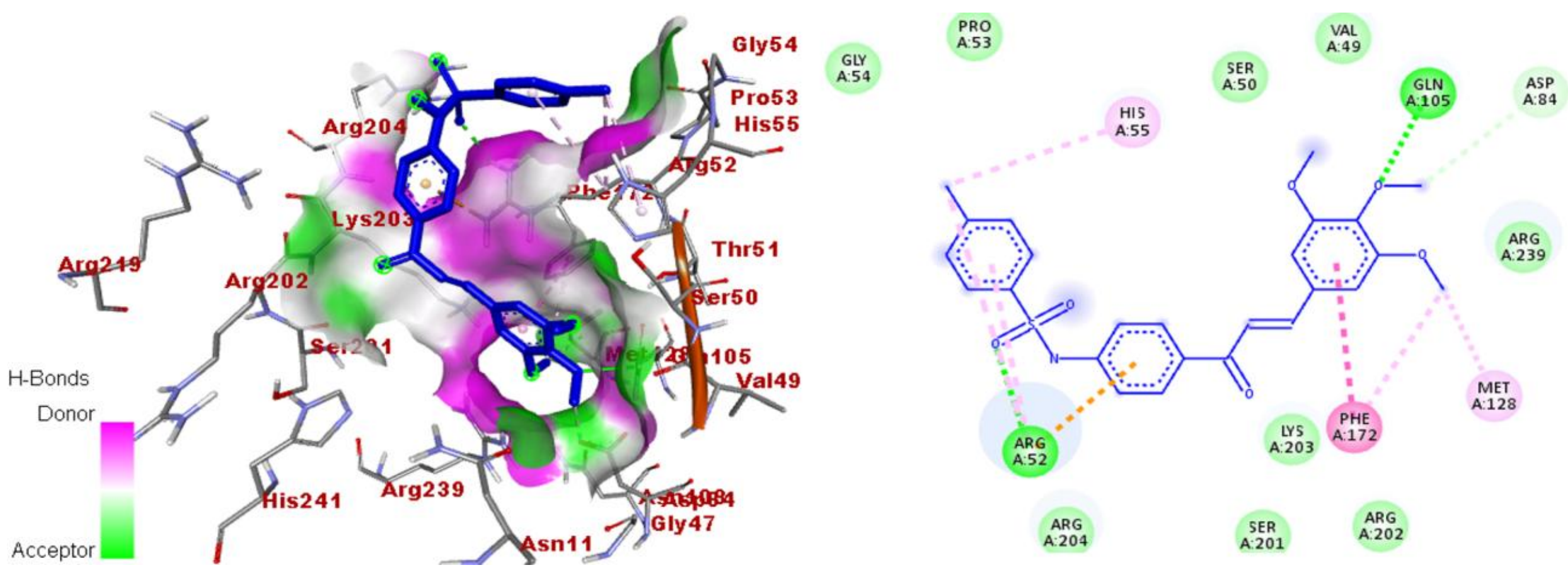

Fig. S42: 3D (left) and 2D (right) representations of the binding interactions of compound **12c** against *S. aureus* DHPS (PDB ID: 1AD4)

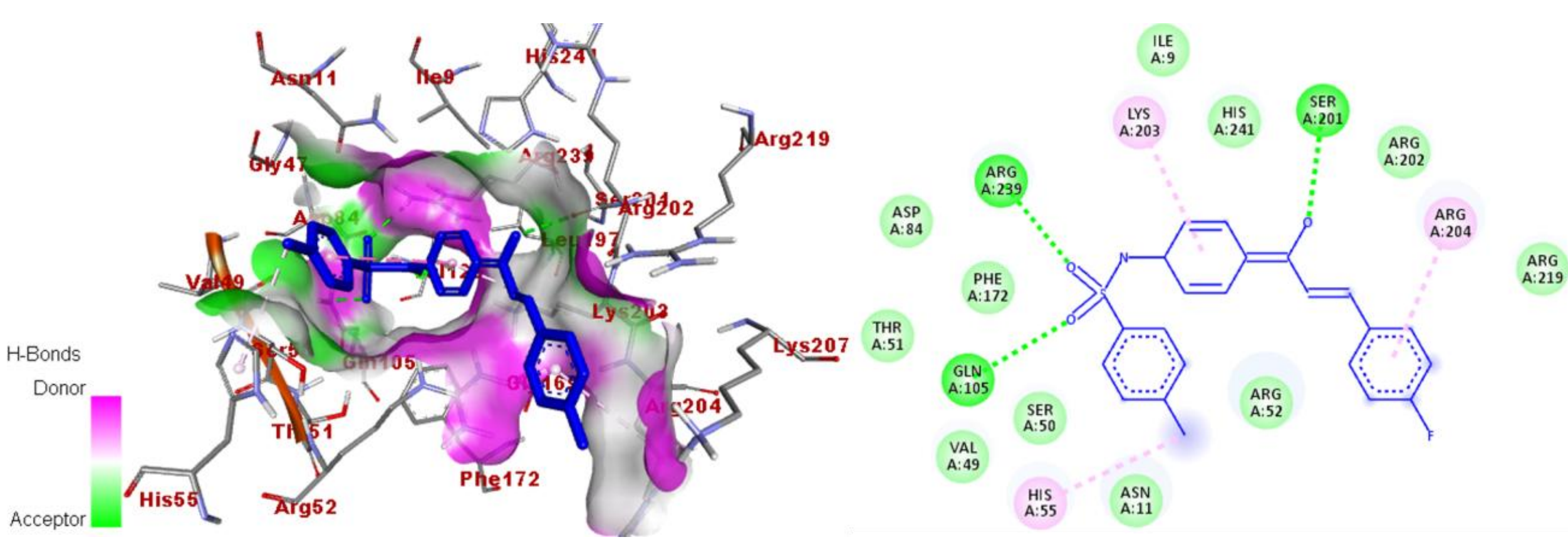

Fig. S43: 3D (left) and 2D (right) representations of the binding interactions of compound **12d** against *S. aureus* DHPS (PDB ID: 1AD4)

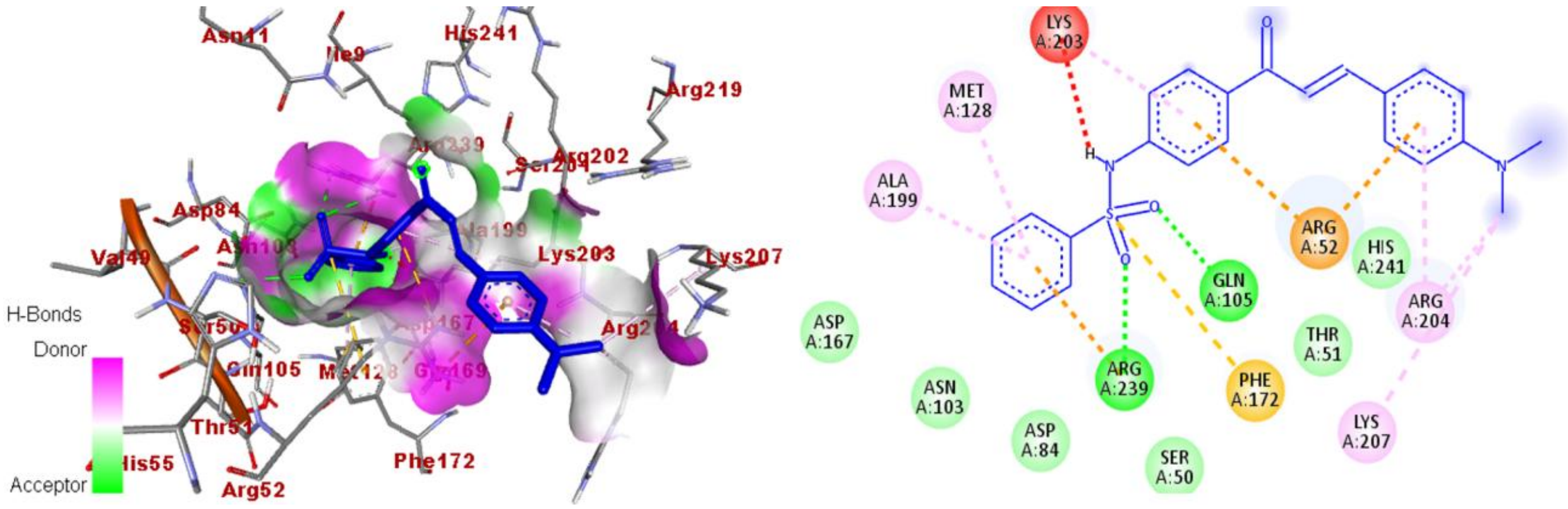

Fig. S44: 3D (left) and 2D (right) representations of the binding interactions of compound **12e** against *S. aureus* DHPS (PDB ID: 1AD4)

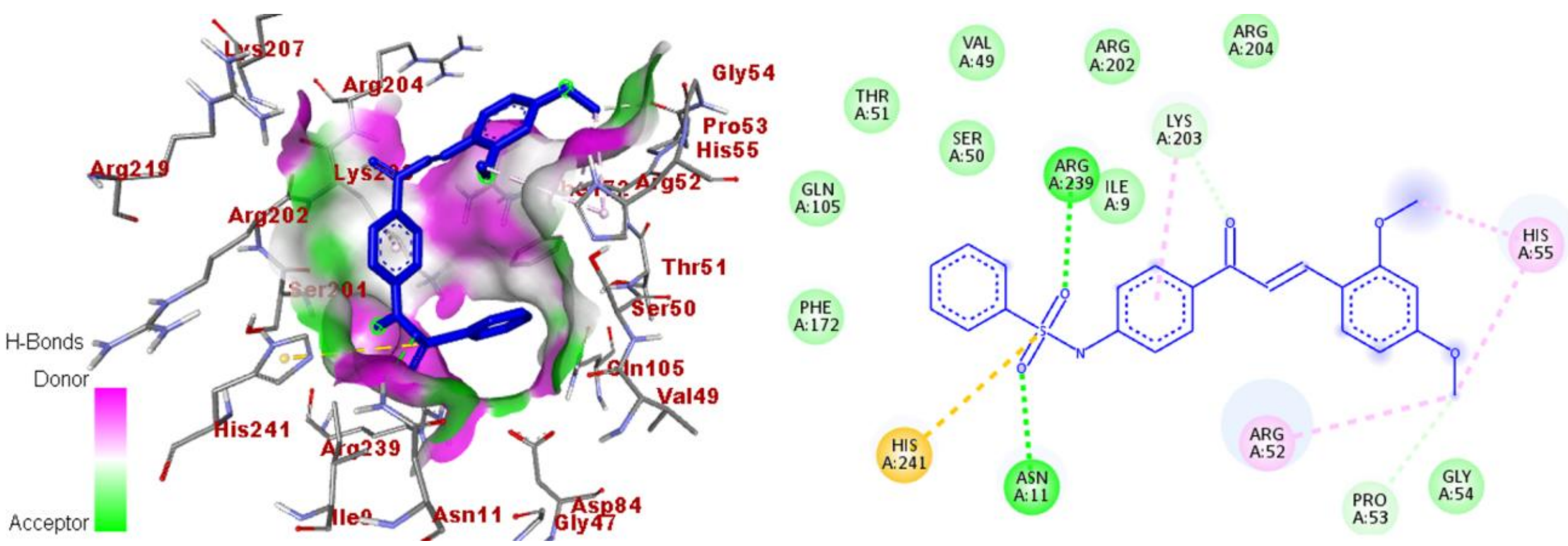

Fig. S45: 3D (left) and 2D (right) representations of the binding interactions of compound **12f** against *S. aureus* DHPS (PDB ID: 1AD4)

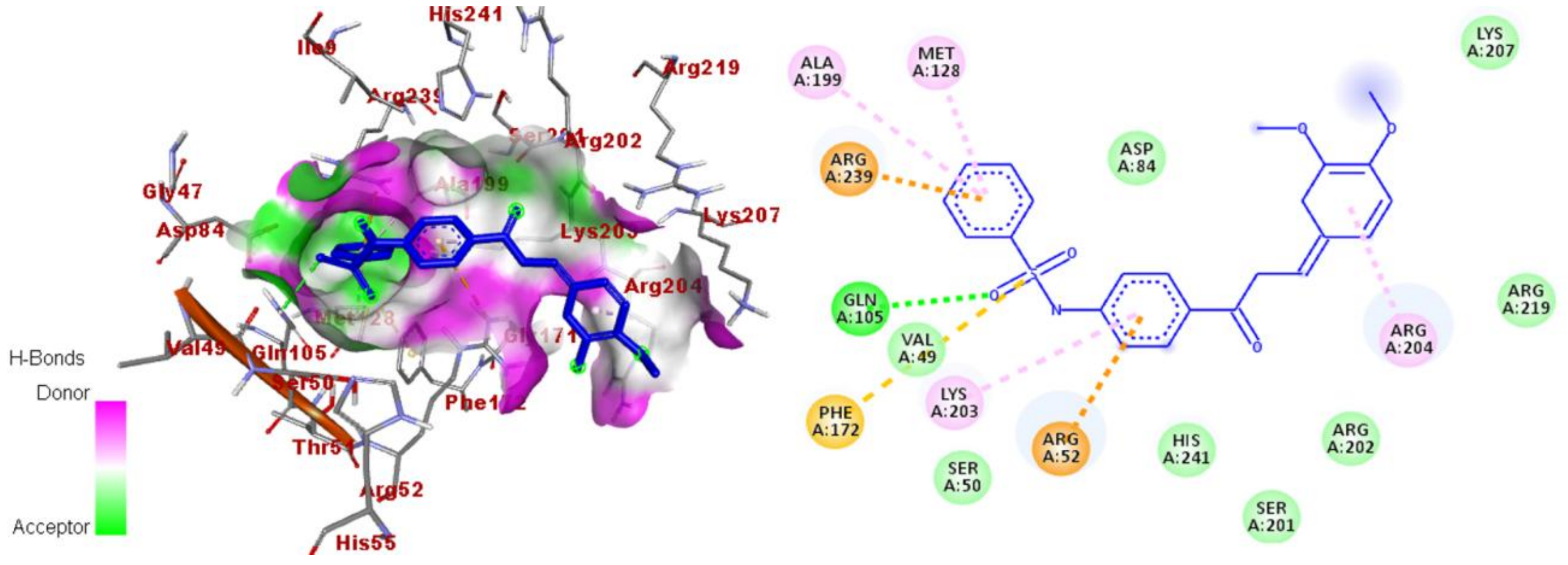

Fig. S46: 3D (left) and 2D (right) representations of the binding interactions of compound **12g** against *S. aureus* DHPS (PDB ID: 1AD4)

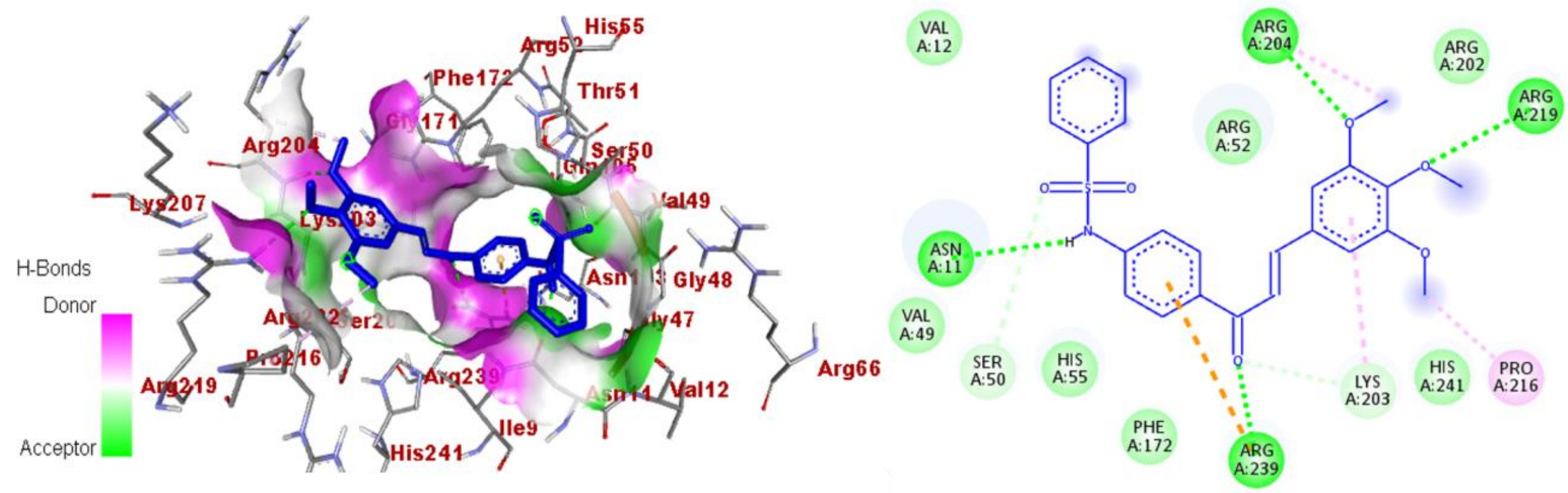

Fig. S47: 3D (left) and 2D (right) representations of the binding interactions of compound **12h** against *S. aureus* DHPS (PDB ID: 1AD4)

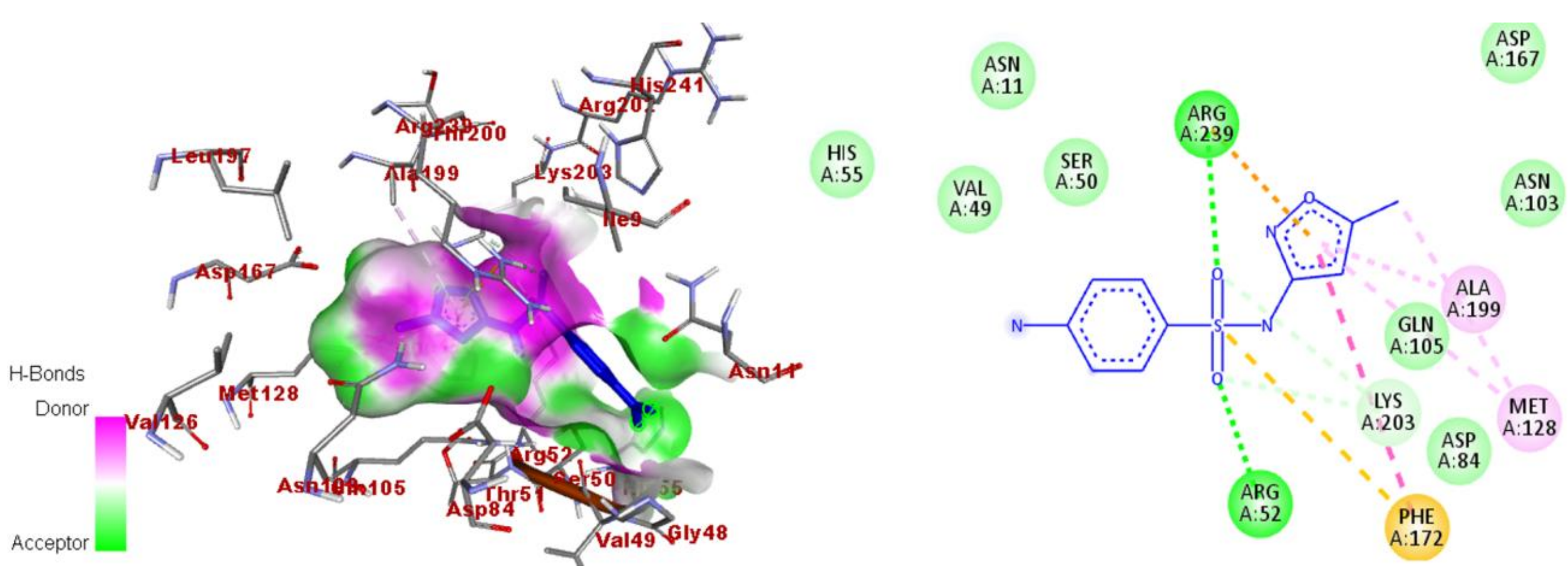

Fig. S48: 3D (left) and 2D (right) representations of the binding interactions of compound **SMX** against *S. aureus* DHPS (PDB ID: 1AD4)

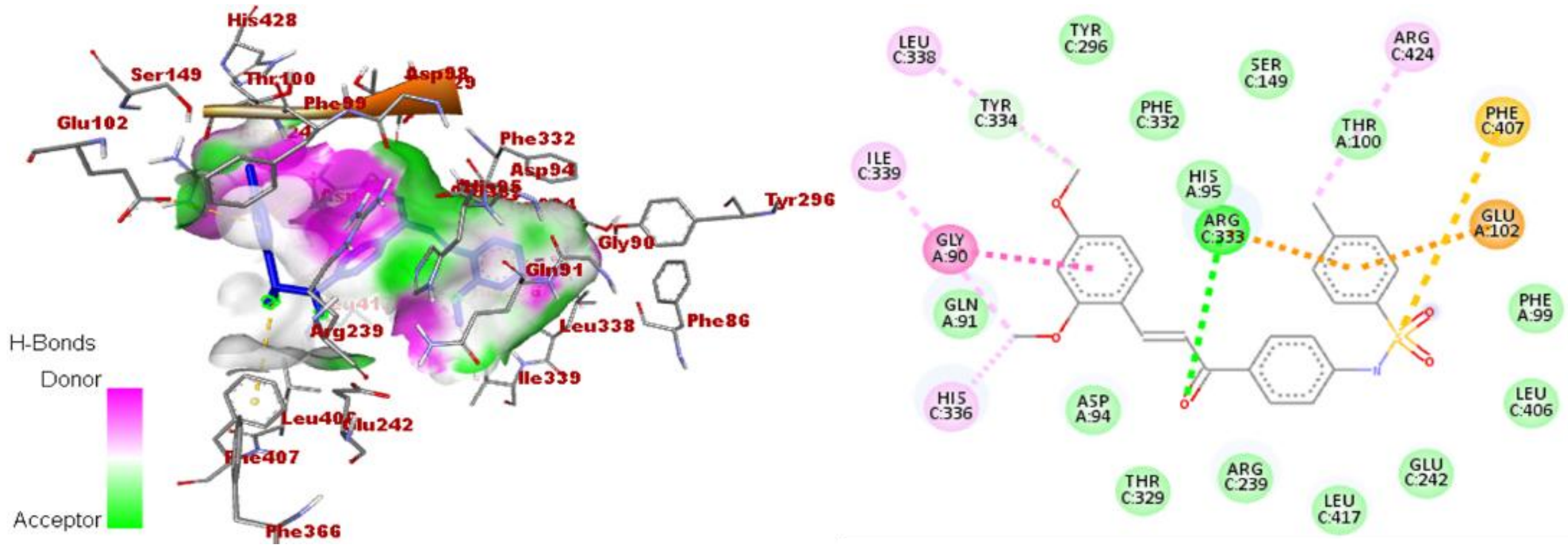

Fig. S49: 3D (left) and 2D (right) representations of the binding interactions of compound **12a** against human MPO (PDB ID: 1DNU)

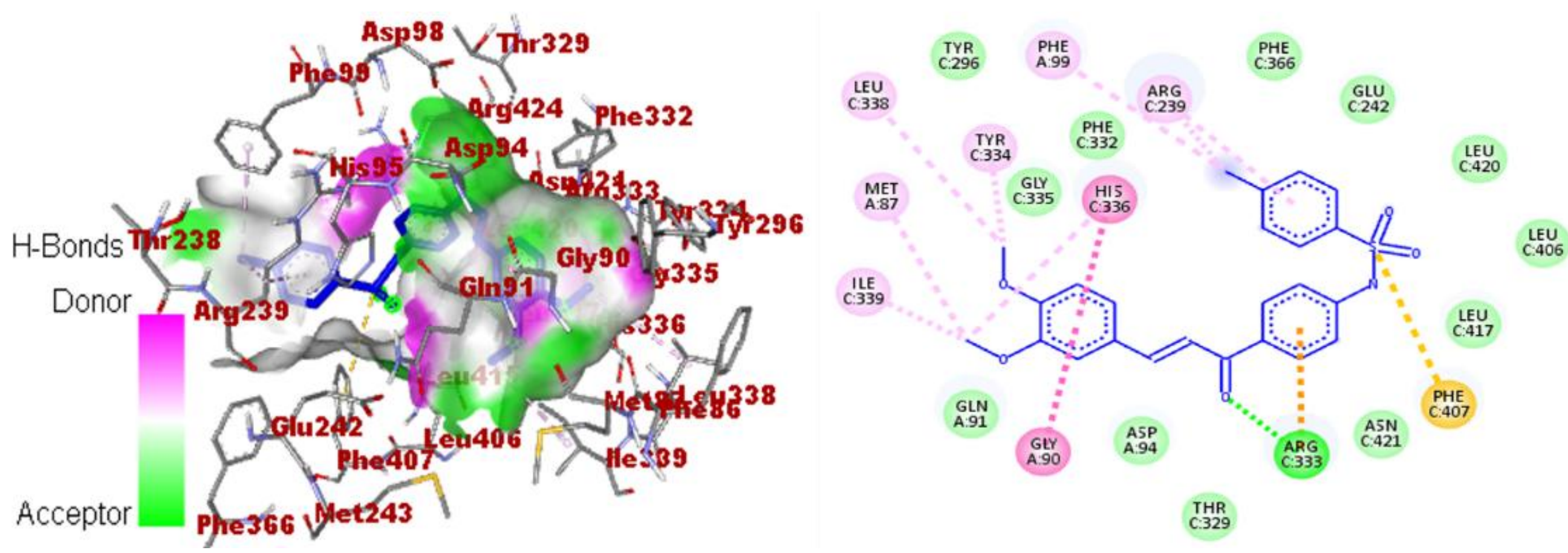

Fig. S50: 3D (left) and 2D (right) representations of the binding interactions of compound **12b** against human MPO (PDB ID: 1DNU)

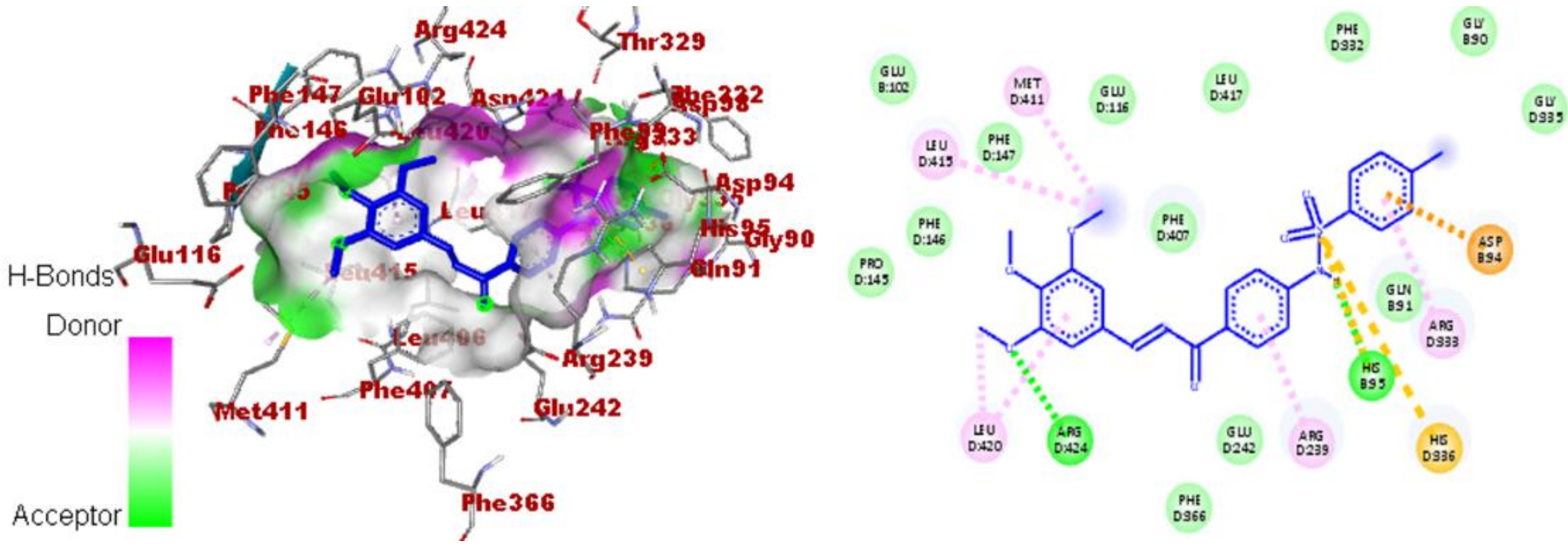

Fig. S51: 3D (left) and 2D (right) representations of the binding interactions of compound **12c** against human MPO (PDB ID: 1DNU)

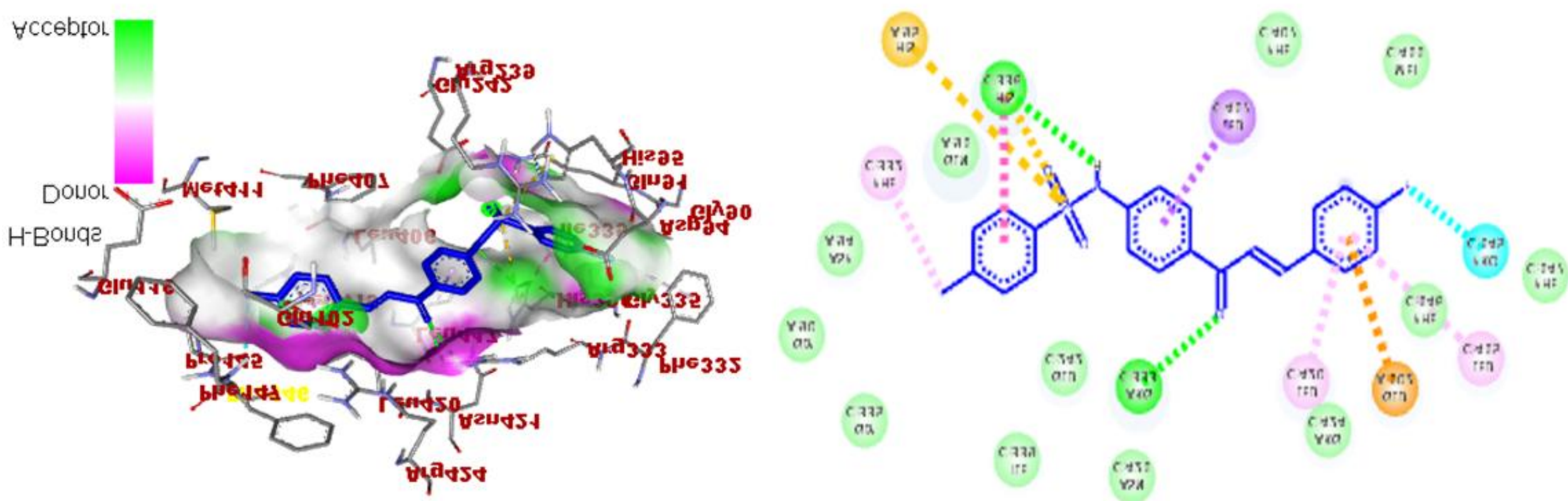

Fig. S52: 3D (left) and 2D (right) representations of the binding interactions of compound **12d** against human MPO (PDB ID: 1DNU)

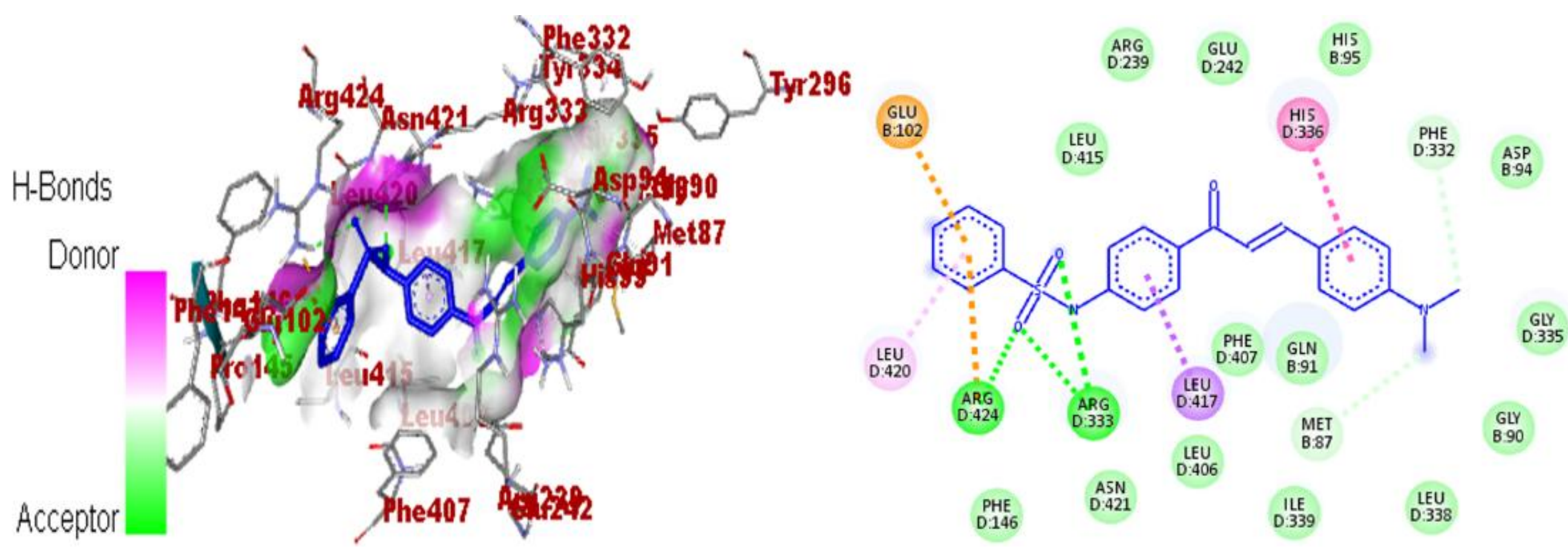

Fig. S53: 3D (left) and 2D (right) representations of the binding interactions of compound **12e** against human MPO (PDB ID: 1DNU)

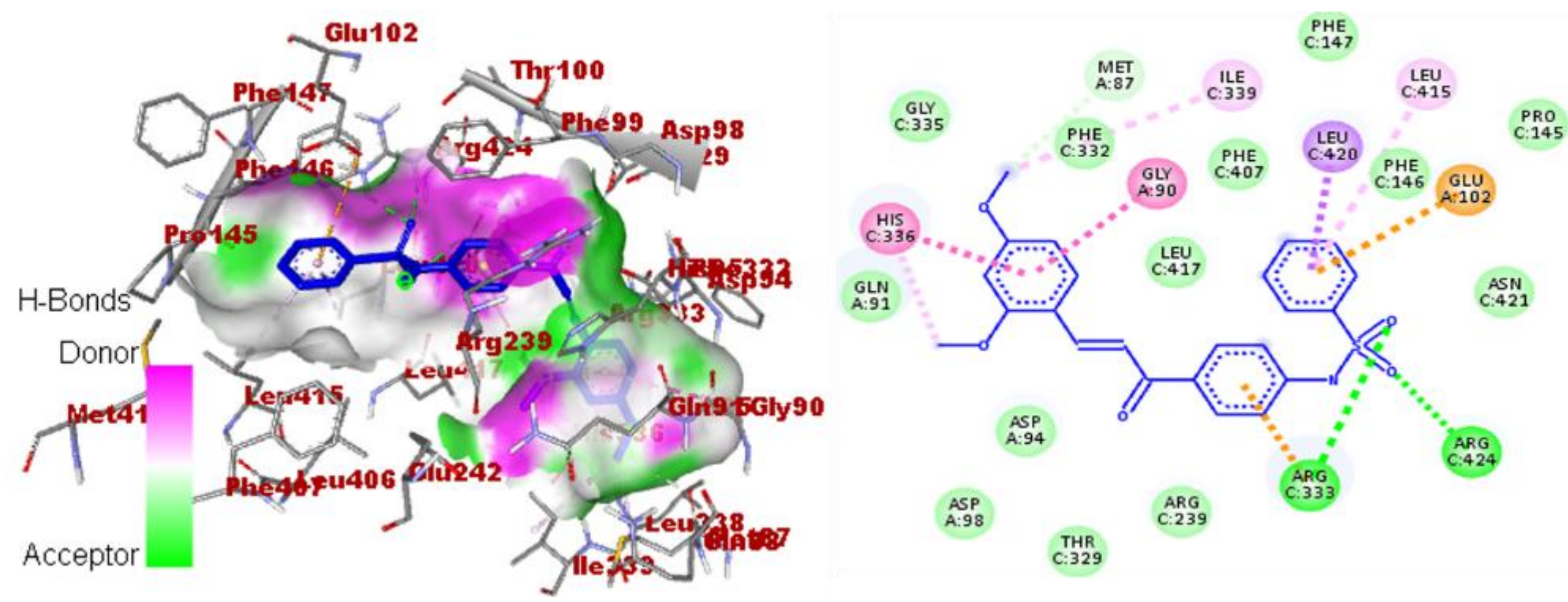

Fig. S54: 3D (left) and 2D (right) representations of the binding interactions of compound **12f** against human MPO (PDB ID: 1DNU)

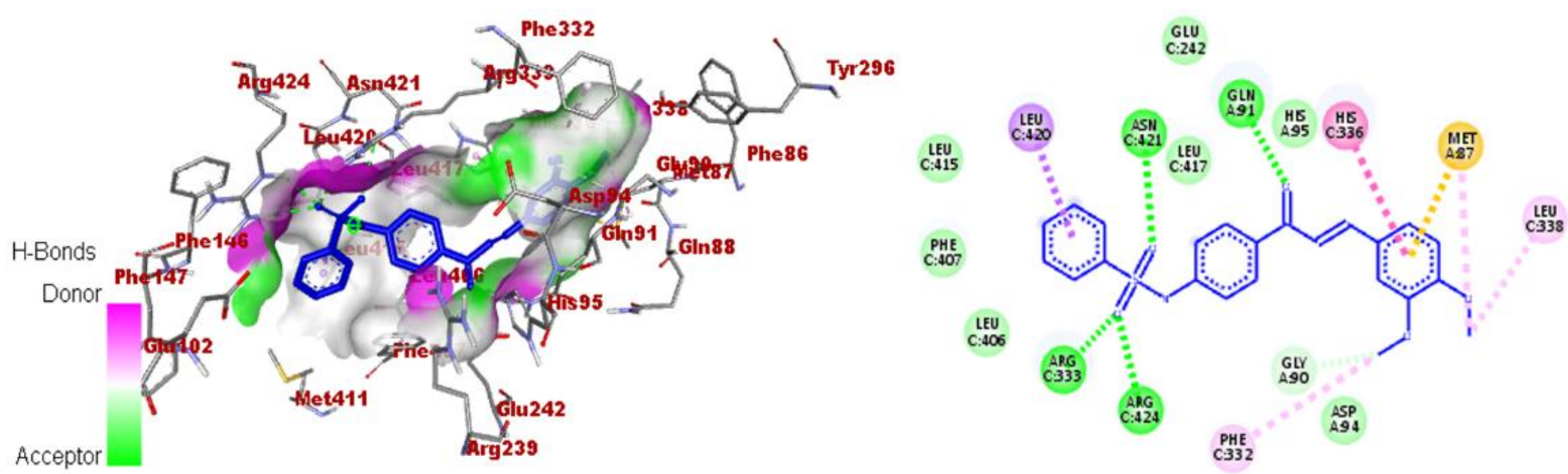

Fig. S55: 3D (left) and 2D (right) representations of the binding interactions of compound **12g** against human MPO (PDB ID: 1DNU)

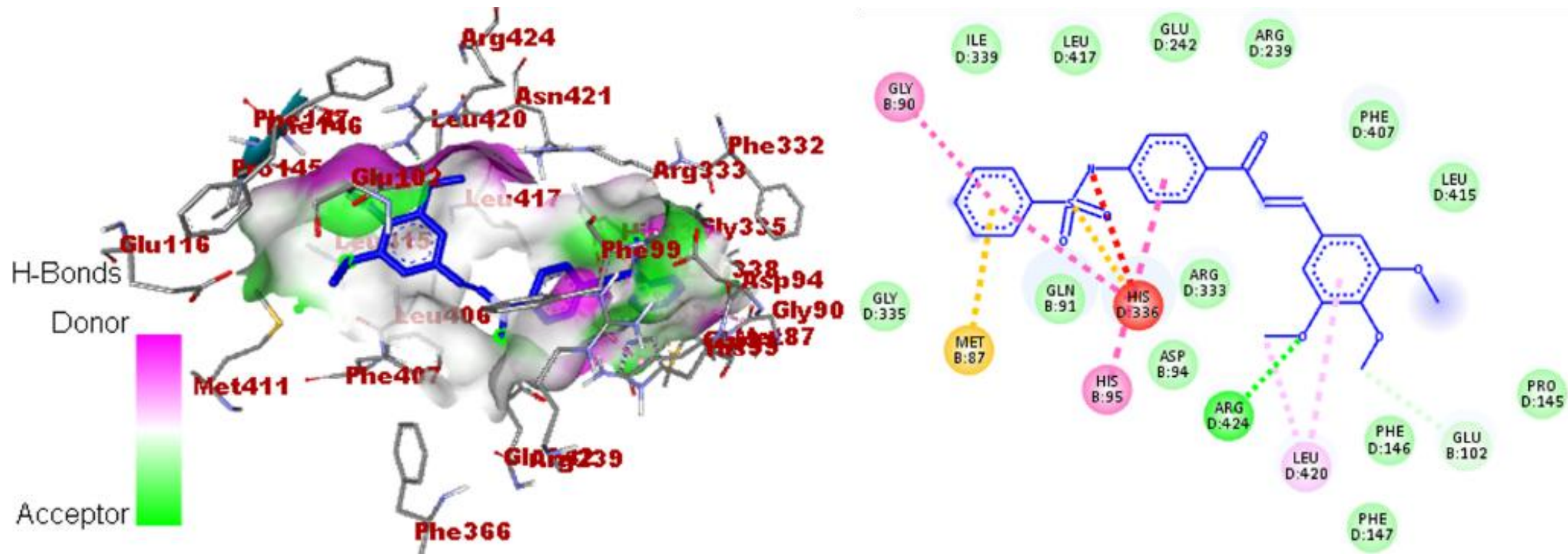

Fig. S56: 3D (left) and 2D (right) representations of the binding interactions of compound **12h** against human MPO (PDB ID: 1DNU)

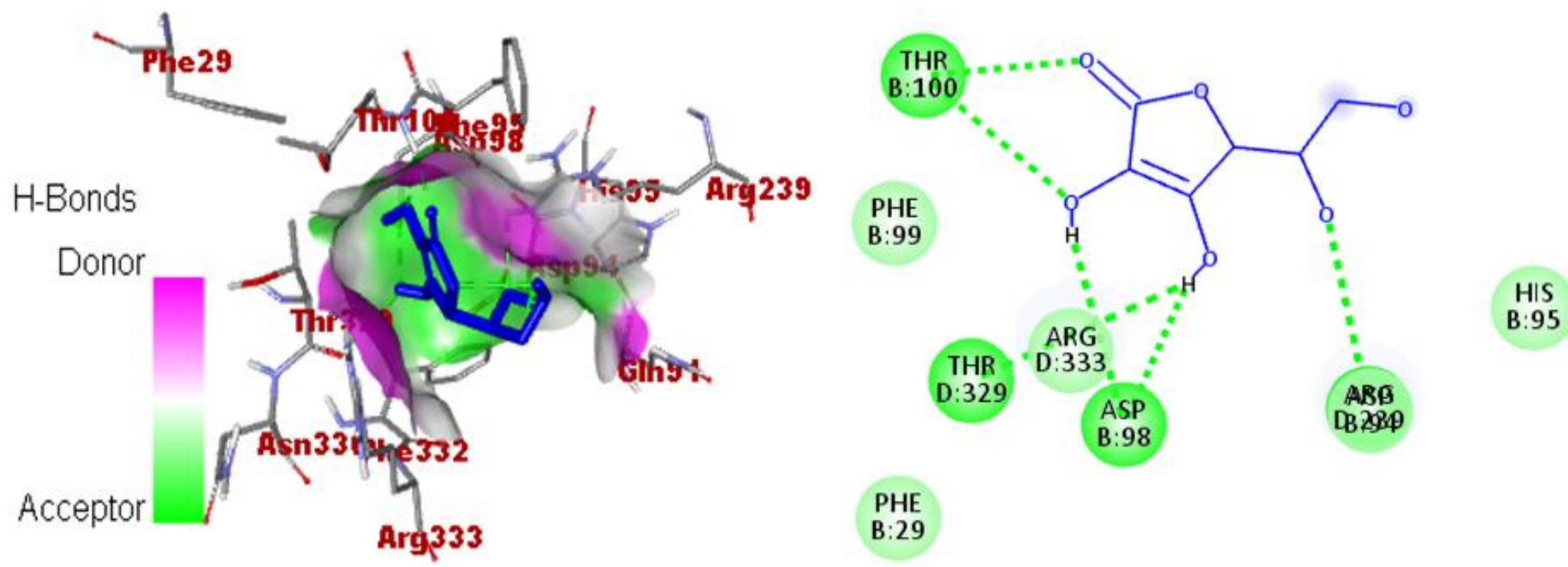

Fig. S57: 3D (left) and 2D (right) representations of the binding interactions of **ascorbic acid** against human MPO (PDB ID: 1DNU)

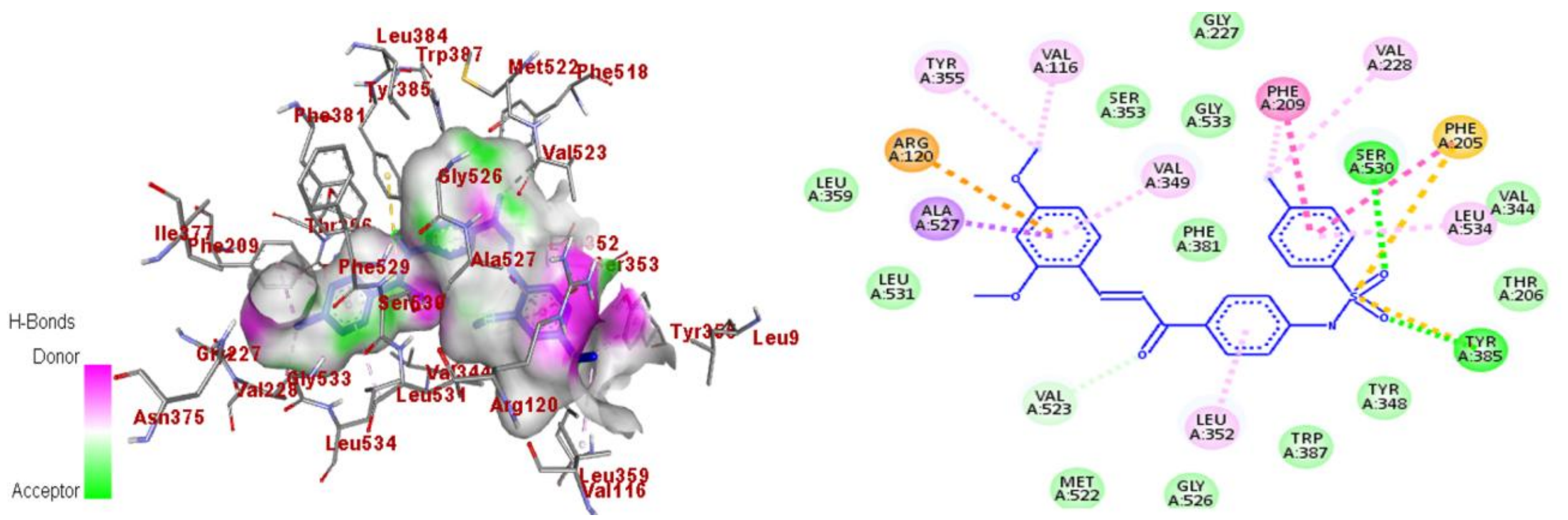

Fig. S58: 3D (left) and 2D (right) representations of the binding interactions of compound **12a** against COX-2 (PDB ID: 5IKR)

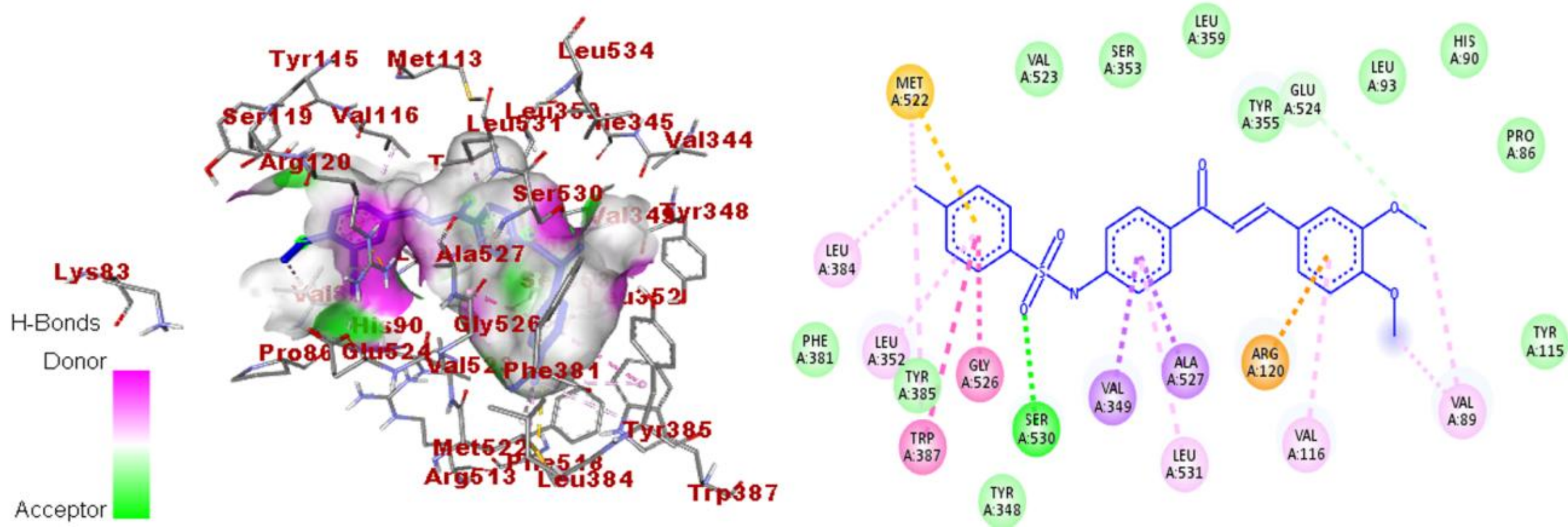

Fig. S59: 3D (left) and 2D (right) representations of the binding interactions of compound **12b** against COX-2 (PDB ID: 5IKR)

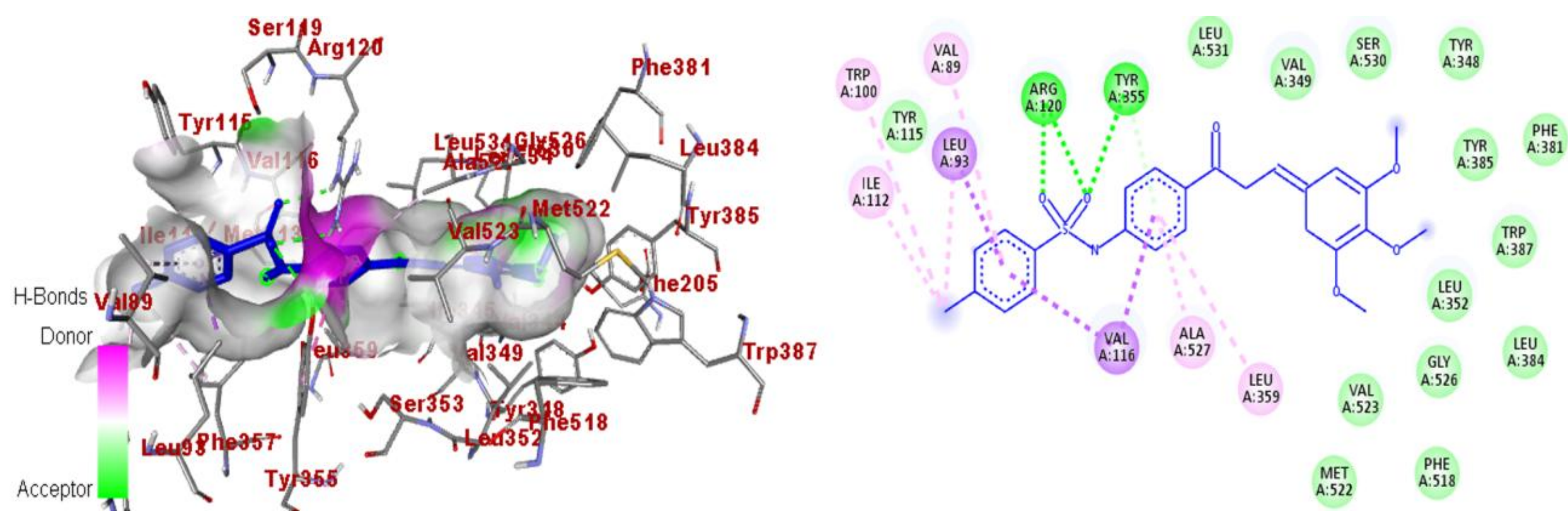

Fig. S60: 3D (left) and 2D (right) representations of the binding interactions of compound **12c** against COX-2 (PDB ID: 5IKR)

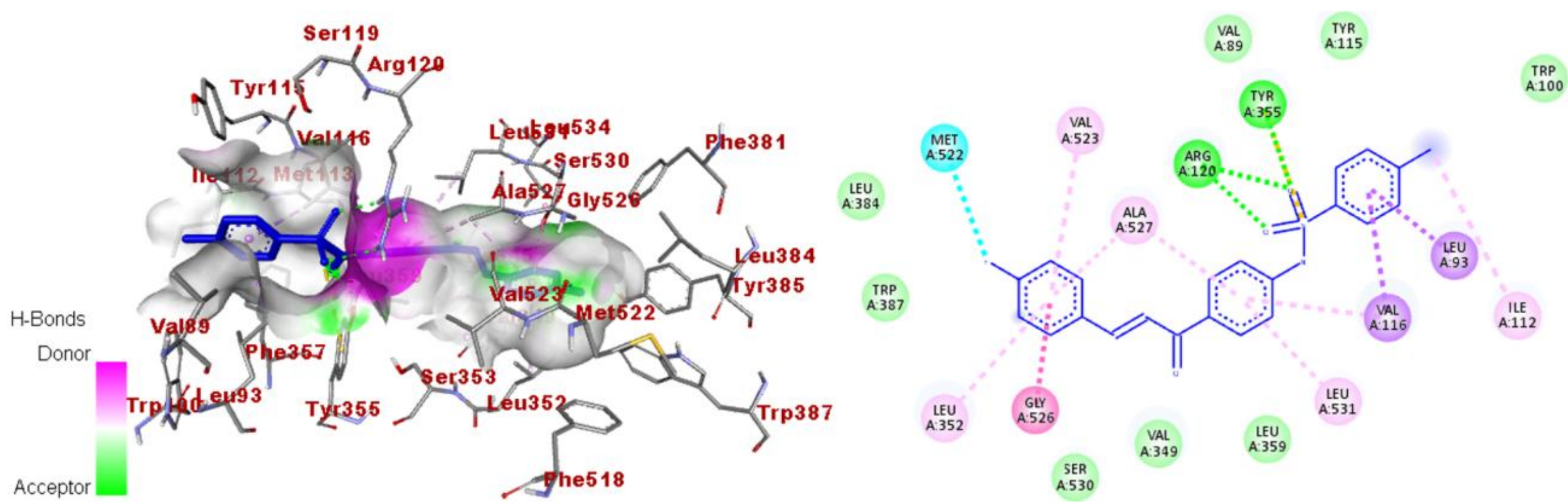

Fig. S61: 3D (left) and 2D (right) representations of the binding interactions of compound **12d** against COX-2 (PDB ID: 5IKR)

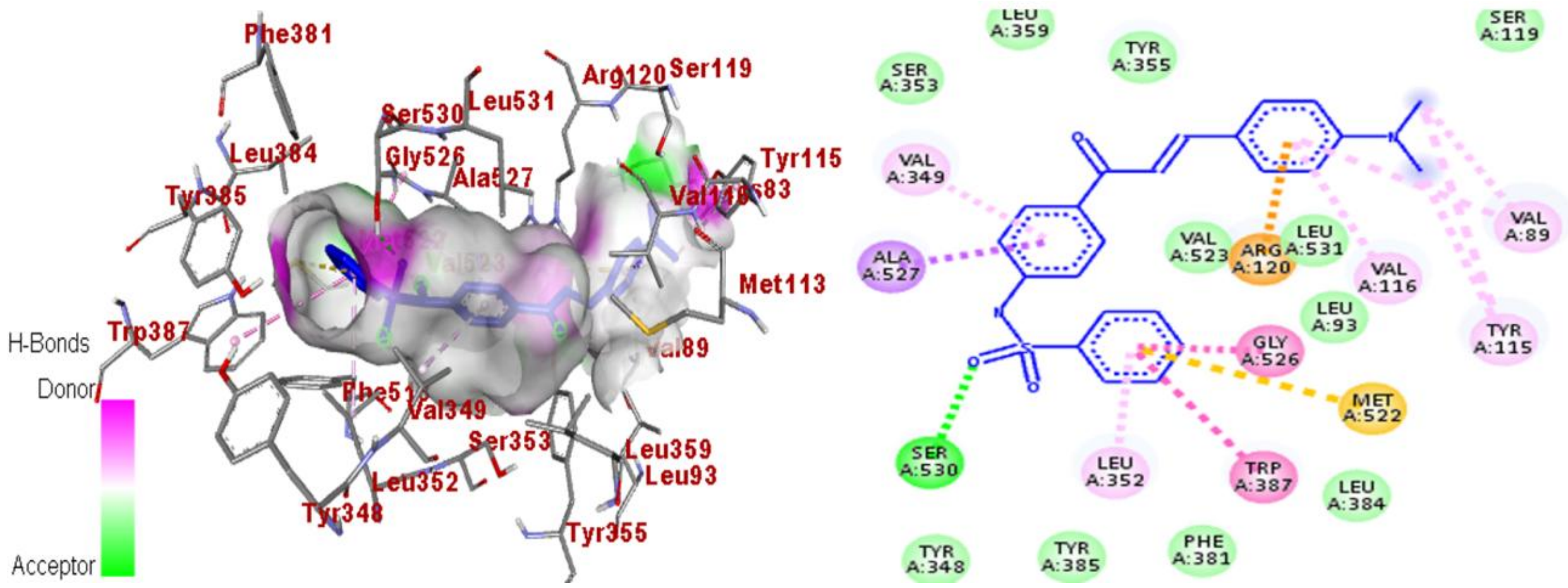

Fig. S62: 3D (left) and 2D (right) representations of the binding interactions of compound **12e** against COX-2 (PDB ID: 5IKR)

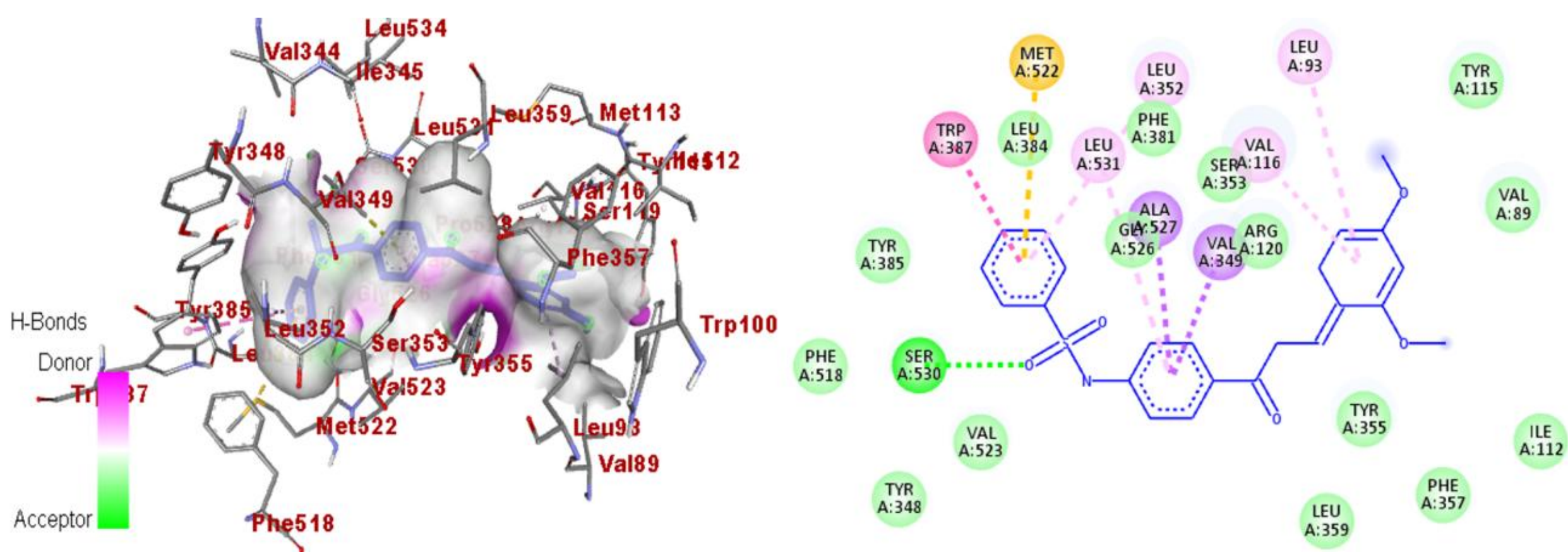

Fig. S63: 3D (left) and 2D (right) representations of the binding interactions of compound **12f** against COX-2 (PDB ID: 5IKR)

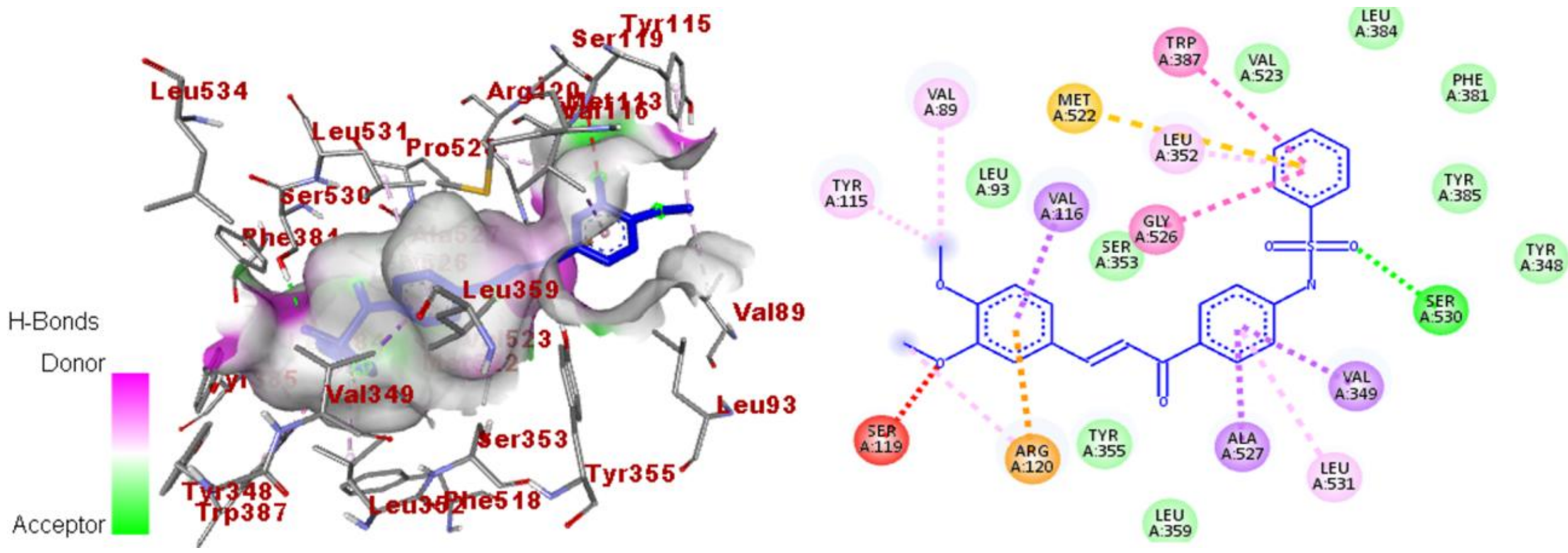

Fig. S64: 3D (left) and 2D (right) representations of the binding interactions of compound **12g** against COX-2 (PDB ID: 5IKR)

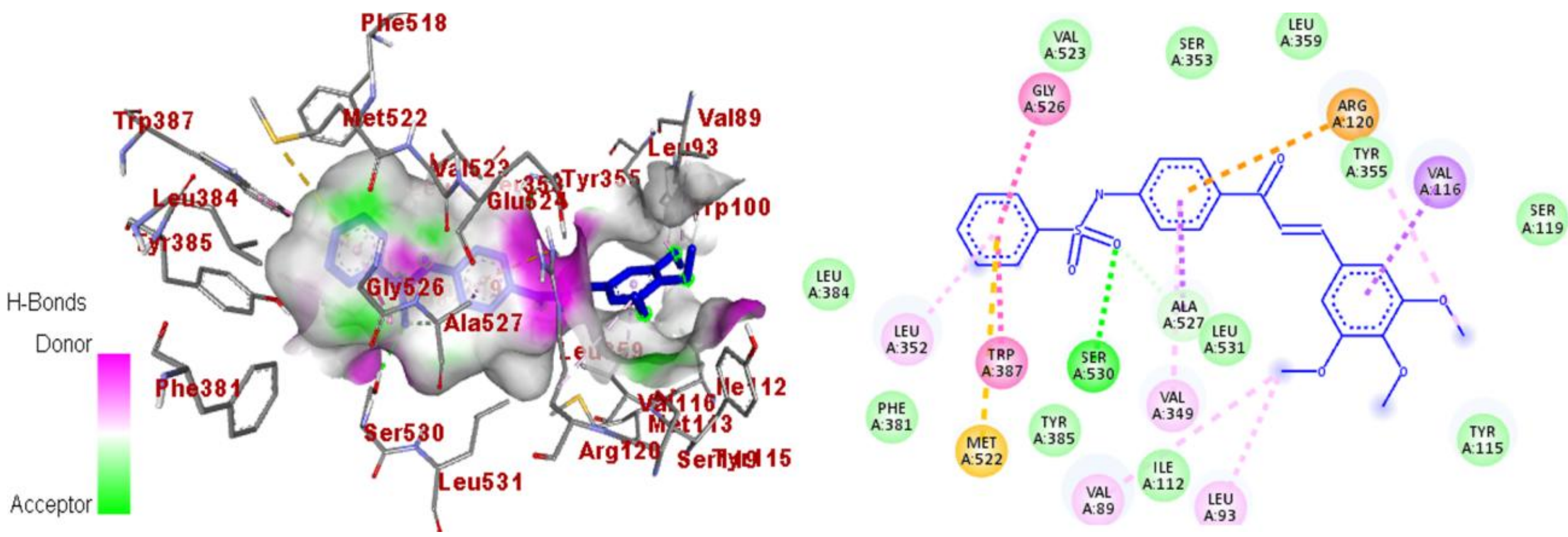

Fig. S65: 3D (left) and 2D (right) representations of the binding interactions of compound **12h** against COX-2 (PDB ID: 5IKR)

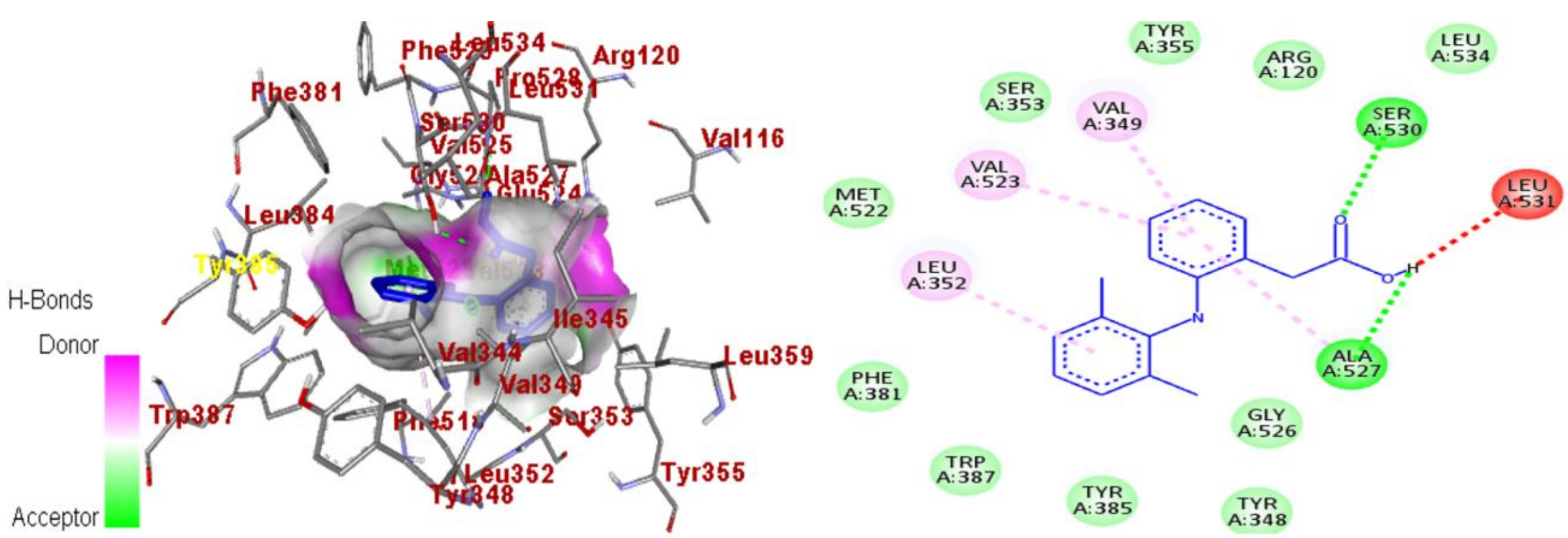

Fig. S66: 3D (left) and 2D (right) representations of the binding interactions of diclofenac against COX-2 (PDB ID: 5IKR)

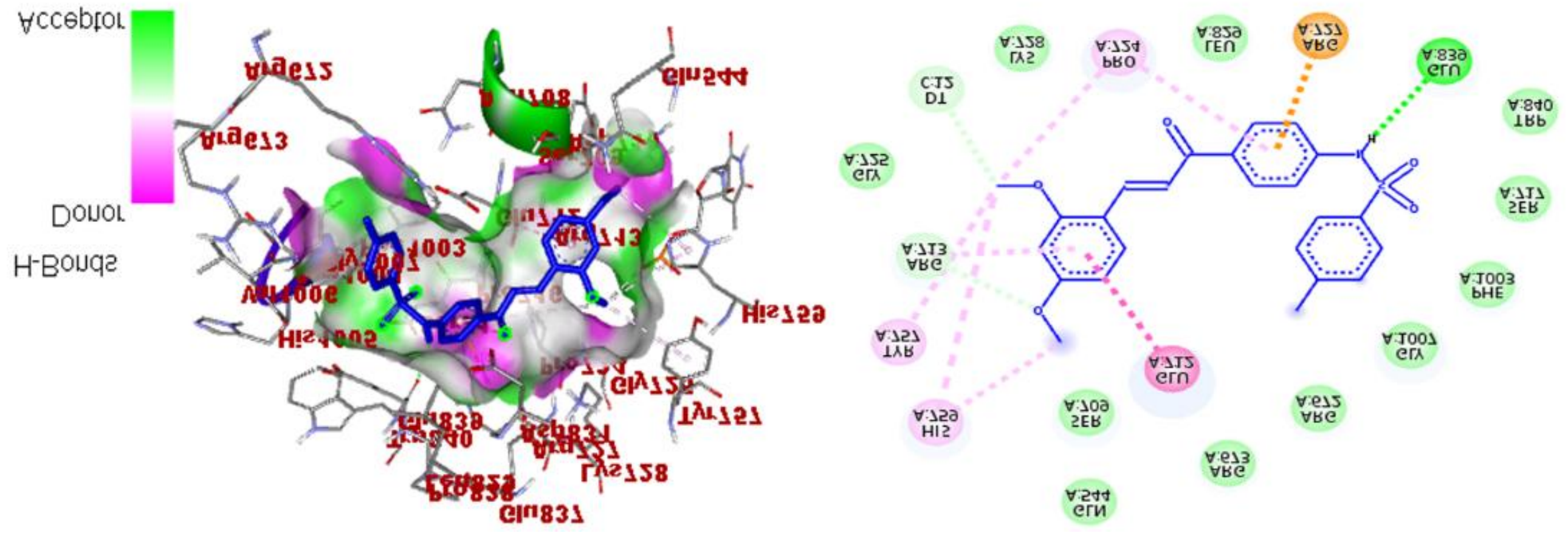

Fig. S67: 3D (left) and 2D (right) representations of the binding interactions of compound **12a** against topoisomerase *IIα* (PDB ID: 4FM96)

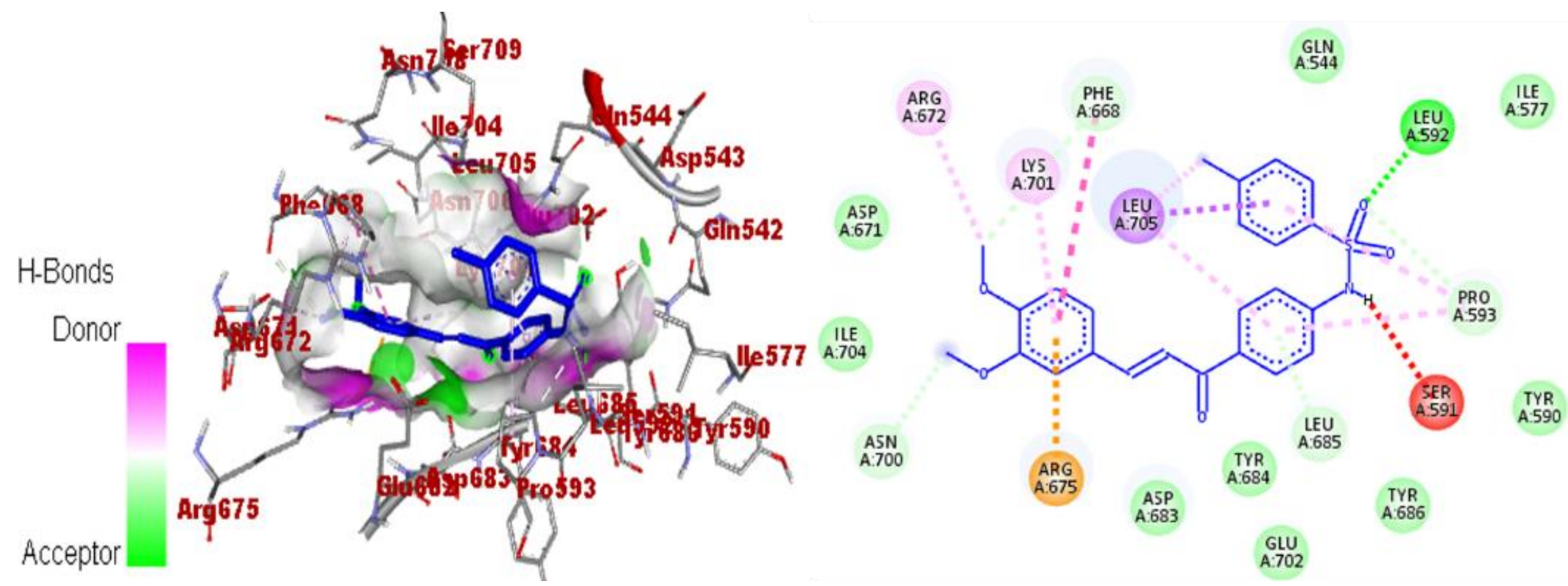

Fig. S68: 3D (left) and 2D (right) representations of the binding interactions of compound **12b** against topoisomerase *IIα* (PDB ID: 4FM96)

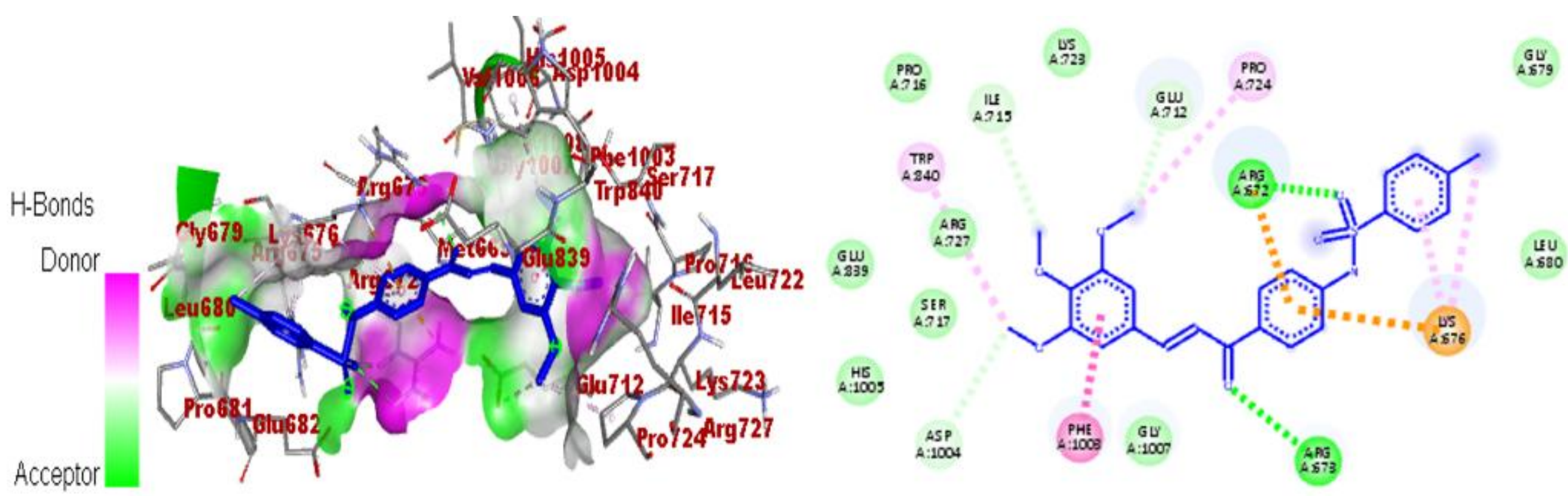

Fig. S69: 3D (left) and 2D (right) representations of the binding interactions of compound **12c** against topoisomerase *IIα* (PDB ID: 4FM96)

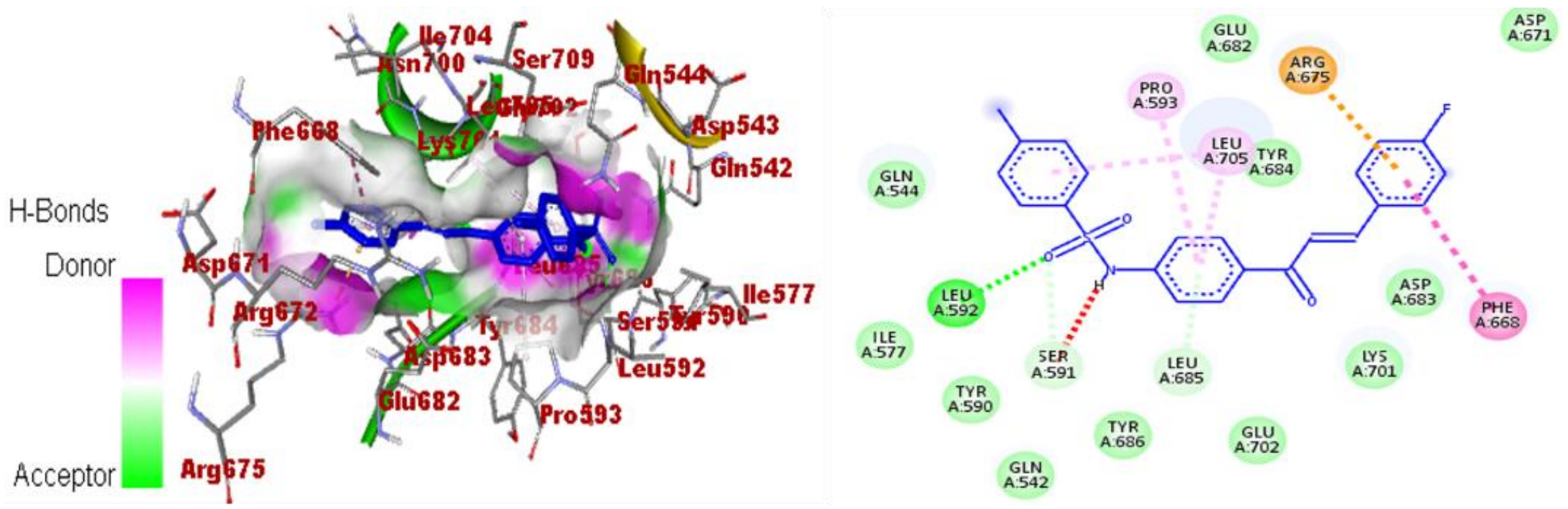

Fig. S70: 3D (left) and 2D (right) representations of the binding interactions of compound **12d** against topoisomerase *IIα* (PDB ID: 4FM96)

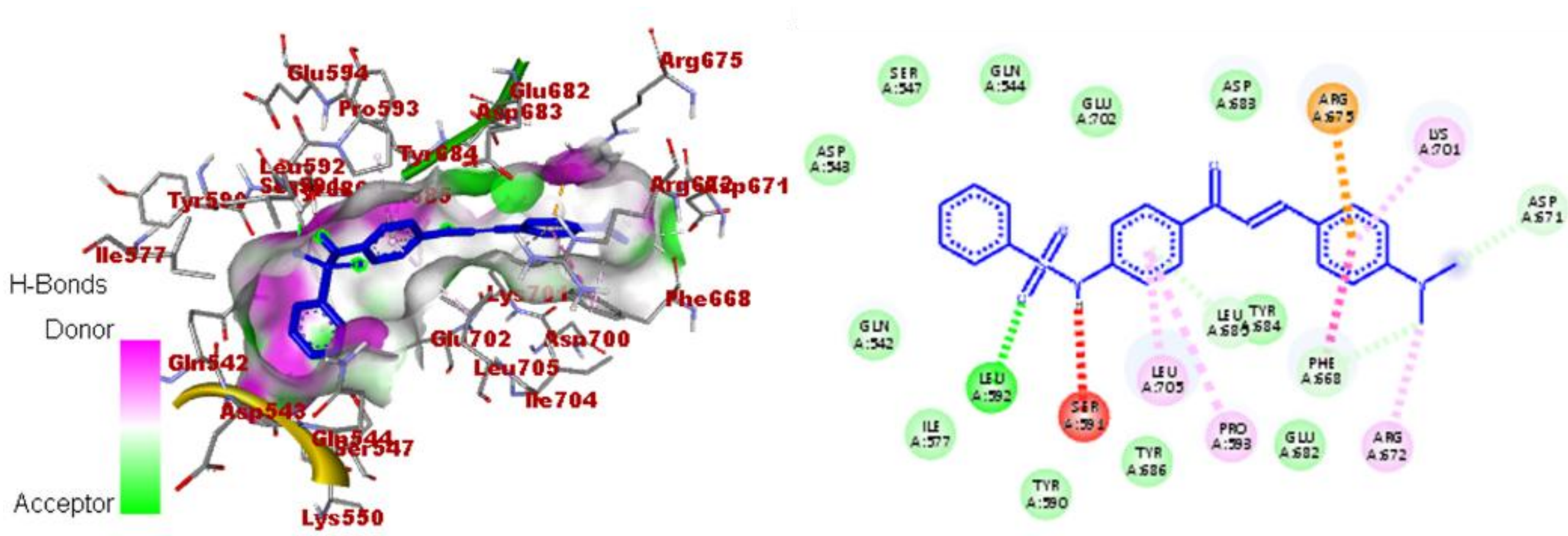

Fig. S71: 3D (left) and 2D (right) representations of the binding interactions of compound **12e** against topoisomerase *IIα* (PDB ID: 4FM96)

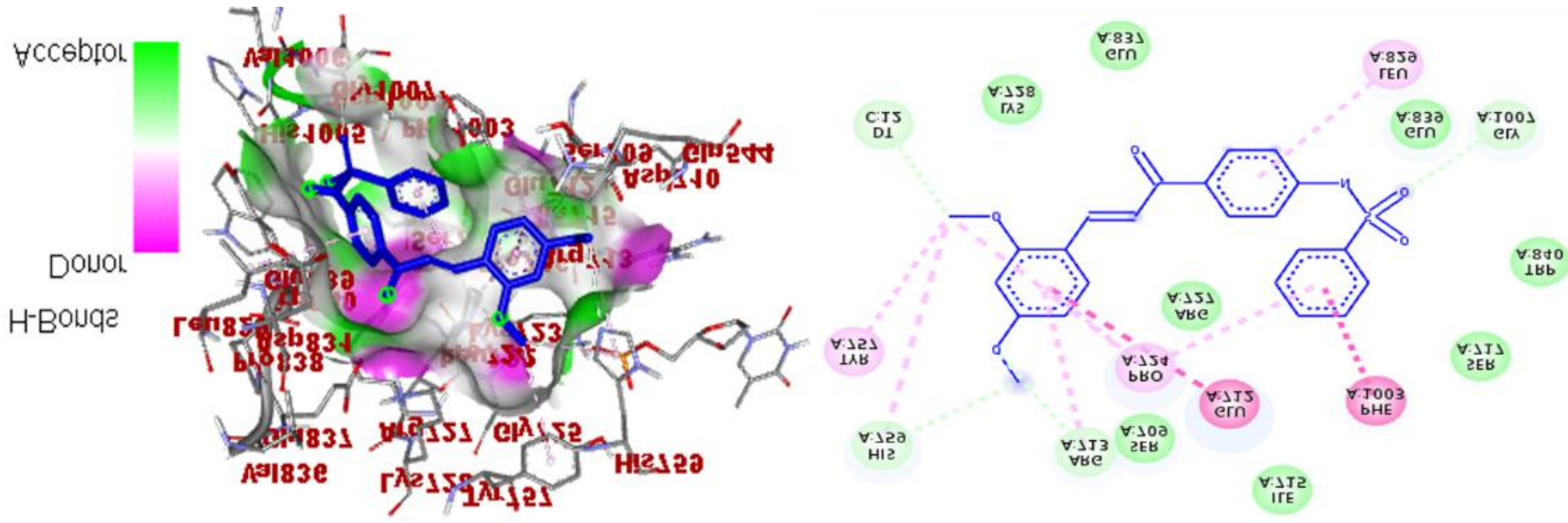

Fig. S72: 3D (left) and 2D (right) representations of the binding interactions of compound **12f** against topoisomerase *IIα* (PDB ID: 4FM96)

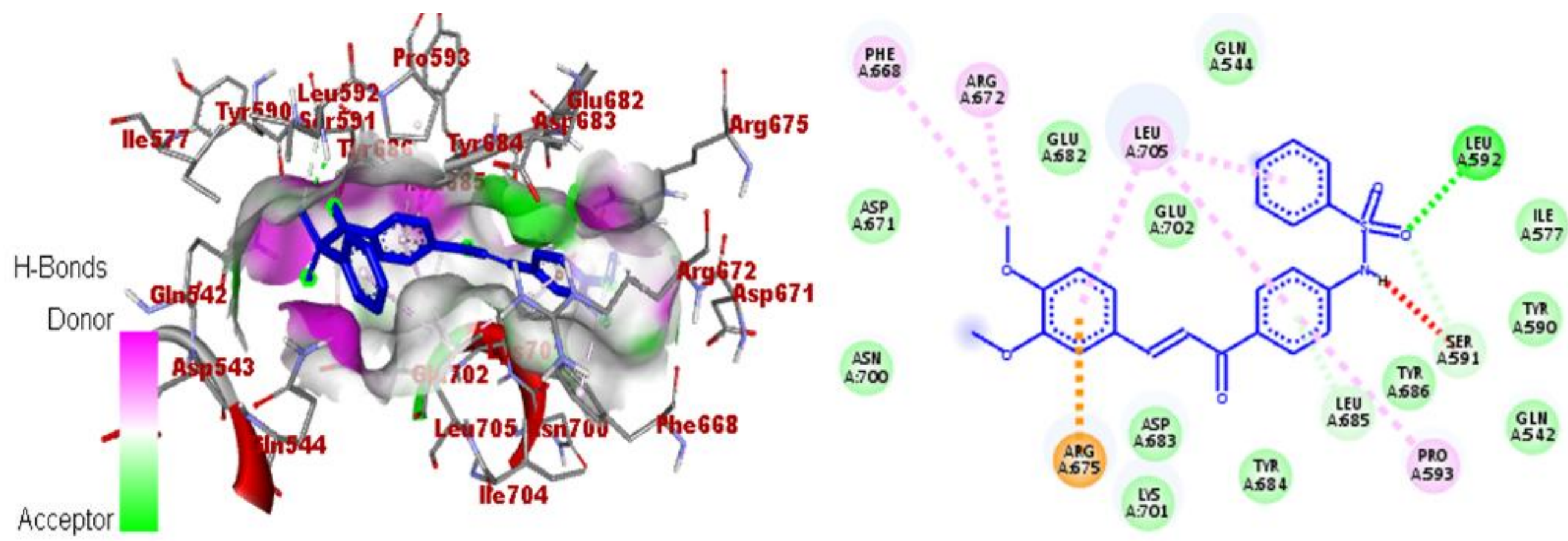

Fig. S73: 3D (left) and 2D (right) representations of the binding interactions of compound **12g** against topoisomerase *IIα* (PDB ID: 4FM96)

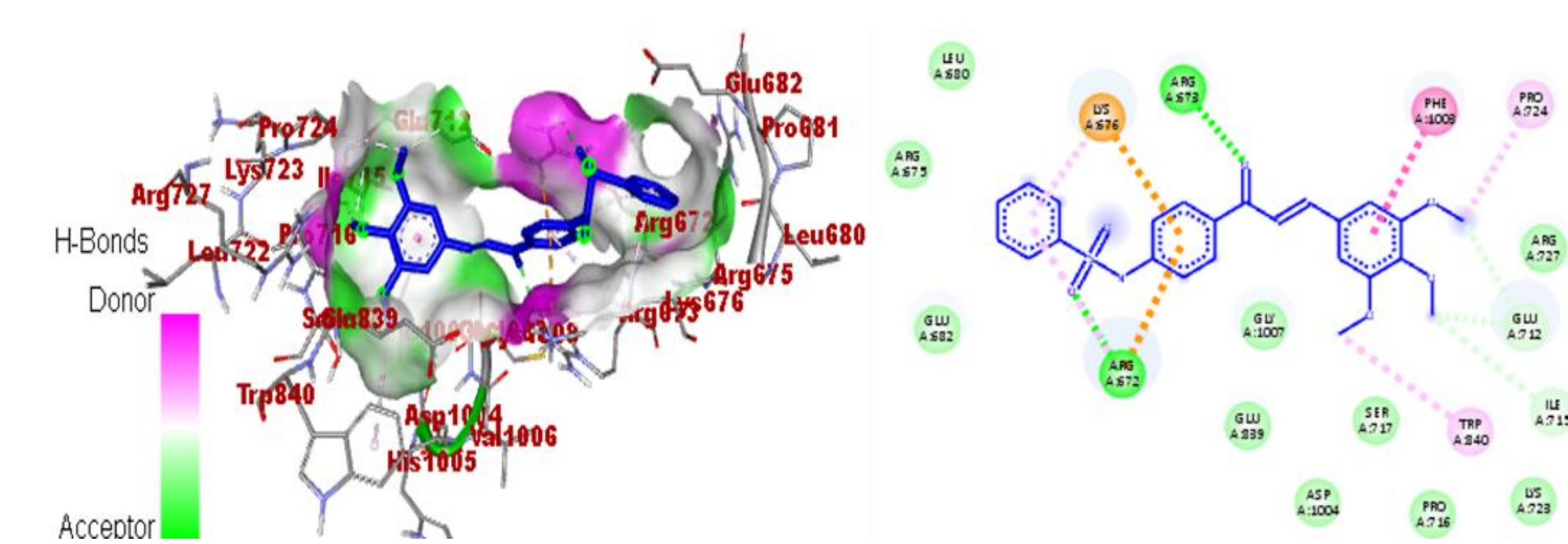

Fig. S74: 3D (left) and 2D (right) representations of the binding interactions of compound **12h** against topoisomerase *IIα* (PDB ID: 4FM96)

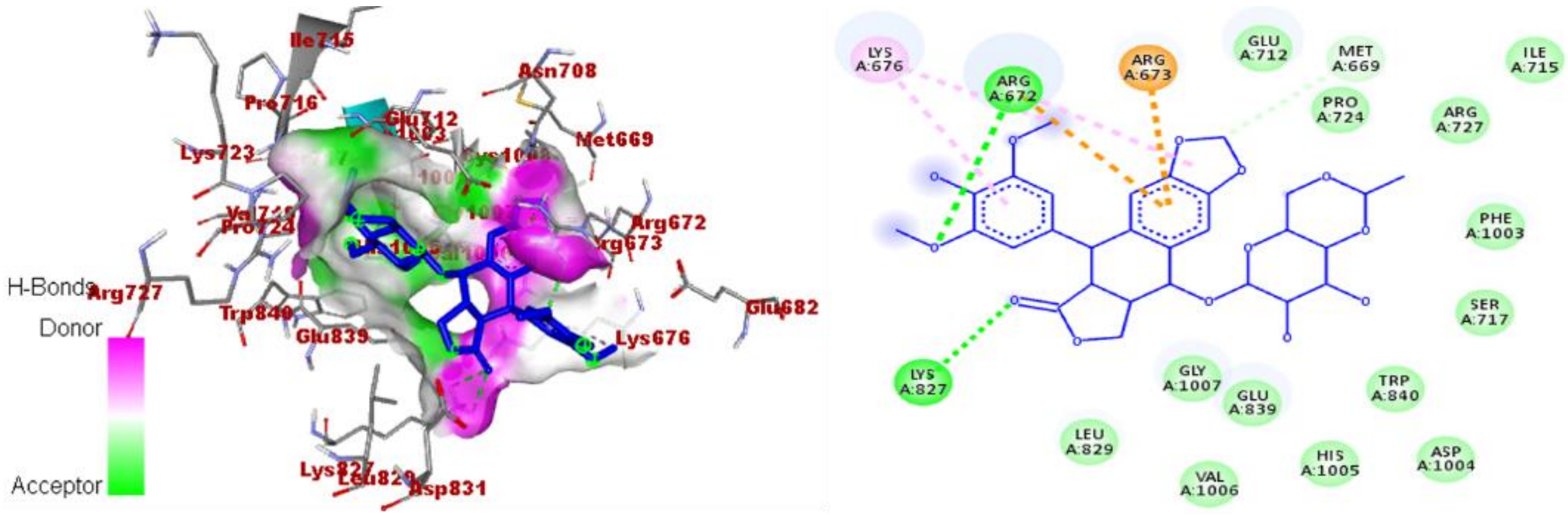

Fig. S75: 3D (left) and 2D (right) representations of the binding interactions of compound **etoposide** against topoisomerase *IIα* (PDB ID: 4FM96)

Table S6: Drug-likeness predictions of synthesized compounds, computed by SwissADME.

Compounds	Parameter							
	Formula	Molecular Weight (g/mole)	nHBD	nHBA	nRB	TPSA ($A^{\circ 2}$)	LogP (iLogP)	Lipinski's Rule Violation
**12a**	$C_{24}H_{23}NO_5S$	437.51	1	5	8	90.08	3.47	0
**12b**	$C_{24}H_{23}NO_5S$	437.51	1	5	8	90.08	3.42	0
**12c**	$C_{25}H_{25}NO_6S$	467.53	1	6	9	99.31	3.6	0
**12d**	$C_{22}H_{18}FNO_3S$	395.45	1	4	6	71.62	2.91	0
**12e**	$C_{23}H_{22}N_2O_3S$	406.5	1	3	7	74.86	2.95	0
**12f**	$C_{23}H_{21}NO_5S$	423.48	1	5	8	90.08	3.25	0
**12g**	$C_{23}H_{21}NO_5S$	423.48	1	5	8	90.08	3.17	0
**12h**	$C_{24}H_{23}NO_6S$	453.51	1	6	9	99.31	3.32	0
**SMZ**	$C_{10}H_{11}N_3O_3S$	253.28	2	4	3	106.60	1.03	0

Table S7: ADME Predictions of Synthesized Compounds Computed by SwissADME and PreADMET

Parameter	Synthesized Compounds								
	**12a**	**12b**	**12c**	**12d**	**12e**	**12f**	**12g**	**12h**	**SMZ**
Gastrointestinal Absorption	High	High	High	High	High	High	High	High	High
Blood Brain Barrier (BBB)	No	No	No	No	No	No	No	No	No
P-gp Substrate Inhibitor	No	No	No	No	No	No	No	No	No
CYP1A2 Inhibitor	No	No	No	Yes	Yes	No	No	No	No
CYP2C19 Inhibitor	Yes	Yes	Yes	Yes	Yes	Yes	Yes	Yes	No
CYP2C9 Inhibitor	Yes	Yes	Yes	Yes	Yes	Yes	Yes	Yes	No
CYP2D6 Inhibitor	No	No	No	No	No	No	No	No	No
CYP2A4 Inhibitor	Yes	Yes	Yes	Yes	Yes	Yes	Yes	Yes	No
LogKp (cm/s)	-5.76	-5.76	-5.96	-5.40	-5.93	-5.93	-5.93	-6.13	-7.21

Table S8: Toxicity prediction of synthesized compounds computed by ProTox-II

	Synthesized Compounds								
**Toxicity**	12a	12b	12c	12d	12e	12f	12g	12h	SMZ
Hepato	Mo	Mo	Mo	No	No	Mo	Mo	Mo	Mo
Carcino	No	No	No	No	No	No	No	No	Mo
Immuno	No	No	Mo	No	No	No	No	Mo	NO
Mutagen	No	No	No	No	No	No	No	No	No
Cyto	No	No	No	No	No	No	No	No	No
LD_{50} (mg/kg)	6000	6000	6000	6000	6000	6000	6000	6000	2300
Toxicity Class	6	6	6	6	6	6	6	6	5
